# Sulphopreussomerins: Sulfur-Containing Preussomerins Isolated from the Marine-Derived Fungus *Pyrenochaetopsis indica*

**DOI:** 10.1021/acs.jnatprod.6c00324

**Published:** 2026-05-15

**Authors:** Georgia Karyofyllidou, Rachel Serrano, Víctor González-Menéndez, Jesús Martín, Thomas A. Mackenzie, Maria C. Ramos, Juan Ortega-Vidal, Olivier P. Thomas, José R. Tormo, Olga Genilloud, Fernando Reyes

**Affiliations:** † 328560 Fundación MEDINA, Centro de Excelencia en Investigación de Medicamentos Innovadores en Andalucía, Avda. del Conocimiento 34, Parque Tecnológico de Ciencias de la Salud, 18016 Armilla, Granada, Spain; ‡ School of Biological and Chemical Sciences, Ryan Institute, 8799 University of Galway, H91TK33 Galway, Ireland

## Abstract

The organic extract of *Pyrenochaetopsis indica* CF-301929, a fungal strain collected from the Coral Sea in Australia, showed broad-spectrum activity against the fungal phytopathogens *Zymoseptoria tritici*, *Colletotrichum acutatum*, *Fusarium proliferatum*, and *Magnaporthe grisea*. After extensive purification, five compounds were isolated, including three new sulfur containing preussomerins **1**–**3** along with two previously described molecules, ascochytatin (**4**) and preussomerin G (**5**). The new compounds incorporate rare thioglycolic or 2-hydroxy-3-mercaptopropionic acid moieties in their structures, and they represent the first members of a new class of preussomerins that we have named sulphopreussomerins. Multiple spectroscopic analyses, including HRESIMS and 1D/2D NMR experiments were used to elucidate their structures while the proposed absolute configuration of compound **1** was assigned by comparison of the experimental and theoretical ECD spectra. When tested against a fungal phytopathogen panel, preussomerin G exhibited, as previously reported, strong antifungal activity and cytotoxicity, whereas sulphopreussomerin A and ascochytatin showed moderate antifungal activity against *Z. tritici* with no detectable cytotoxicity.

## Graphical abstract

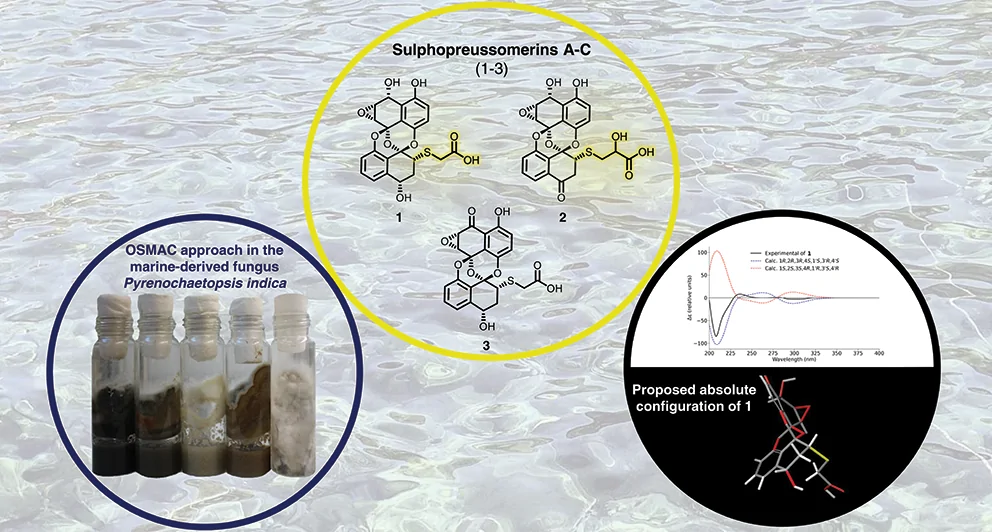

Amid the growing threat of antimicrobial resistance, further investigation of marine biodiversity as a source of new antibiotics is not just promising; it is essential.^1^ Marine natural products represent a promising and largely untapped reservoir of novel antibiotic candidates. Their unique chemical structures, products of ecological and evolutionary pressures of the marine environment, offer new scaffolds for drug development.^2^ This underscores the broader importance of natural products in drug discovery, highlighting the need for interdisciplinary approaches that combine marine biology, chemistry, and pharmacology to harness the therapeutic potential of the ocean.^2^

Fungal secondary metabolites are increasingly recognized as a rich source of structurally diverse natural products with important applications in medicine and biotechnology.^3^ These metabolites, including polyketides, terpenoids, alkaloids, and peptides, display a wide range of biological activities such as antimicrobial, anticancer, and anti-inflammatory effects, making them attractive candidates for drug discovery.^4,5^ Endophytic and marine-derived fungi have gained particular attention due to their capacity to produce novel bioactive compounds, while advances in genome mining, metabolomics, and epigenetic approaches continue to reveal previously inaccessible chemical diversity, highlighting the substantial unexplored potential of fungal natural products.^3,5,6^

In this study, a collection of marine-derived fungal strains from Fundación MEDINA was utilized.^7^ Screening of this collection led to the identification of a strain that produced compounds belonging to the class of spirodioxynaphthalenes. High-resolution TOF-MS analysis, including MS/MS spectra acquisition, revealed a family of sulfur-containing scaffolds that had not previously been reported in the literature.

Spirodioxynaphthalenes are a large group of fungal metabolites comprising two 1,8-dihydroxynaphthalene units connected via a spiroketal linkage.^8^ Spirodioxynaphthalenes are highly oxidized compounds with various hydroxylation and unsaturation patterns. Their structural complexity has attracted the attention of synthetic chemists, leading to the development of numerous innovative synthetic strategies and total syntheses.^9,10^ These natural products exhibit a diverse range of biological activities including cytotoxic, antibacterial, antifungal, antileishmanial and antiplasmodial effects underscoring their significance in medicinal chemistry.^11–15^

Spirodioxynaphthalenes have been classified into three distinct types based on the nature of the different linkages between the two naphthalene units: Type A (two oxygen bridges), Type B (three oxygen bridges), and Type C (two oxygen bridges and one carbon–carbon bridge) (Figure 1).^8^ Type A spirodioxynapthalenes are the most common and represent the majority within this class of compounds.^15^ The first spirodioxynapthalene to be isolated was MK 3018, a palmarumycin, obtained from the fungus *Tetraploa aristata* in 1989.^16^ A characteristic spectroscopic feature of this group is the presence in their ^13^C NMR spectra of a signal around 100 ppm, indicating a deshielded quaternary spiroketal carbon. Type B spirodioxynapthalenes are primarily preussomerins, with the first identified compound being preussomerin A, isolated from *Preussia isomera* in 1990.^17^ A defining characteristic in their ^13^C NMR spectra is the presence of two deshielded quaternary carbon signals, both appearing close to 95 ppm, corresponding to the two spiroketal carbons. The type C spirodioxynapthalenes have only few known members, collectively referred as spiroxins. The first spiroxin identified was spiroxin A, isolated from a marine-derived fungus in 1999.^18^ In ^13^C NMR, a signal around 105 ppm indicates a deshielded quaternary spiroketal carbon. Additionally, the correlation between H7 and C4′ in HMBC spectra establishes the bond between C4′ and C6, confirming that the ring B is directly connected to the ring D via a C–C bond.^18^

**Figure 1:**
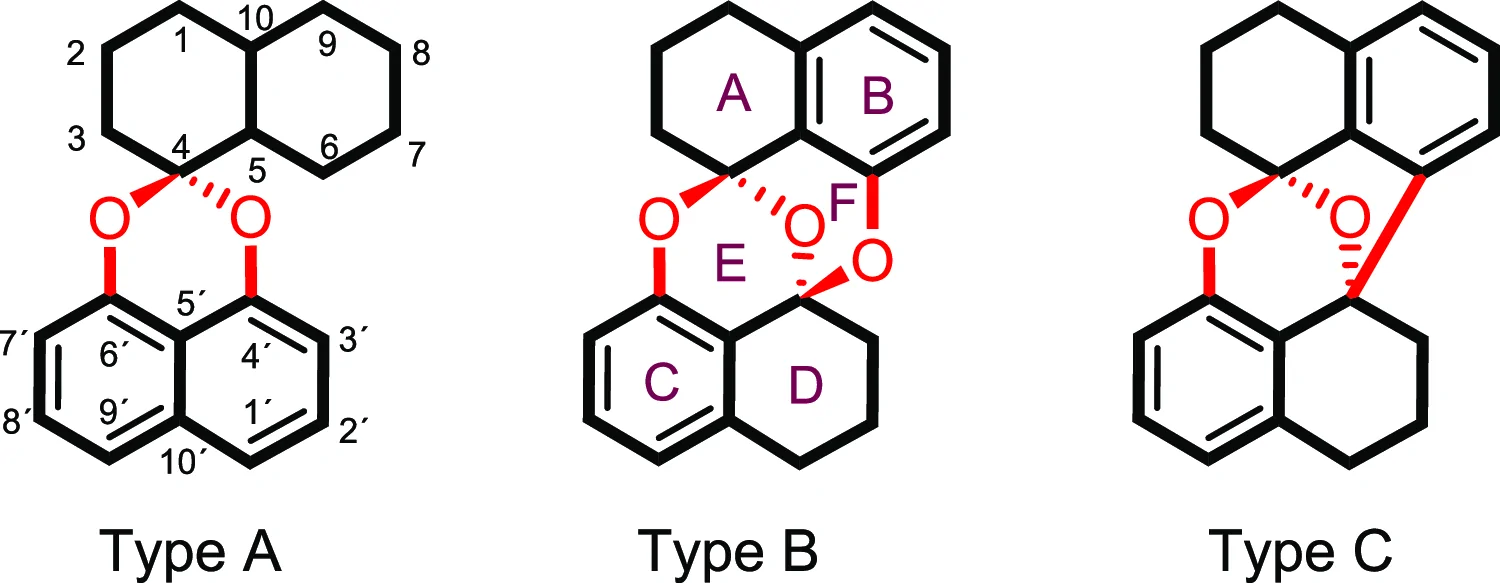
Skeleton structures of spirodioxynaphthalenes, with the region that differentiates each type highlighted in red.

To date, more than 190 spirodioxynaphthalene derivatives have been isolated and characterized, predominantly from fungi, although a few examples have also been reported in plants.^19,25^ It has been suggested that these spirodioxynaphthalenes were likely produced by the endophytic fungi present in the host plant, rather by the plant itself.^26^

Recent studies have focused on characterizing the *pal* biosynthetic gene cluster (BGC) responsible for palmarumycin (type A spirodioxynaphthalene) biosynthesis.^19^ For the biosynthesis of palmarumycins, 1,8-dihydroxynaphthalene (DHN) monomers are required for the dimerization step that leads to their formation.^19,20^ The DHN biosynthetic pathway is common and highly conserved in fungi.^21^ The precursor of DHN, T_4_HN (1,3,6,8-tetrahydroxynaphthalene), is synthesized in fungi by an iterative type I polyketide synthase (PKS).^22^ DHN-melanin biosynthesis is well studied in fungal species, including *Metarhizium robertsii*, *Colletotrichum lagenarium*, *Alternaria alternata*, *Magnaporthe oryzae*, *Botrytis cinerea*, *Aspergillus* spp., *Verticillium dahliae*, *Elsinoë fawcettii*, and *Cladosporium phlei*.^21,23,24^ To date, there is no publicly available genome assembly for *Pyrenochaetopsis indica* or any member of the genus. The way fungi produce the first intermediate, T_4_HN, can differ depending on the specific PKS involved.^21^ DHN BGCs have been reported to share PKS genes, a THN-reductase and a prefoldin-encoding gene, so it is also expected to be present in the genome of *P. indica*.^19,21^

The *pal* BGC is not clustering together with the PKS gene cluster. The pal BGC encodes two cytochrome P450 enzymes, PalA and PalB, along with a short-chain dehydrogenase/reductase, PalC, as key structural proteins.^19^ An additional oxidoreductase, encoded by *palD*, is located outside the cluster.^14^ PalA catalyzes the oxidative dimerization of DHN, forming both the spiroketal linkage and the 2,3-epoxy group. Despite that the preussomerin BGC pathway has not been described so far, it has been proposed that its biosynthesis involve an additional oxidative coupling between the upper and lower naphthalene units, and that a palmarumycin hydroxylated at C-6 may be the precursor of type II spirodioxinaphtalenes.^14^ A later study by Zhai et al. disrupted the *Egpks* gene using CRISPR-Cas9 gene editing, resulting in loss of preussomerin production and suggesting a biosynthetic link to the DHN pathway, confirming the hypothesis that preussomerins and palmarumycins share a common biosynthetic route.^15,27^

This study has evaluated the metabolite production of strain *P. indica* CF-301929 in five different media (DEX-SOY, LSFM, SMK-II, YES, BRFT) using a One Strain Many Compounds (OSMAC)^18^ approach to enhance metabolite production, and the resulting organic extracts were tested against a panel of fungal phytopathogens. Among the tested media, BRFT was identified as the most appropriate for large-scale cultivation (Figure S2), given its effective metabolite production (Figure S1) and broad-spectrum activity of the extract against fungal phytopathogens, including *Zymoseptoria tritici*, *Fusarium proliferatum*, *Colletotrichum acutatum*, and *Magnaporthe grisea* (Table S1).

LC/MS analysis revealed the presence of minor structurally related preussomerin analogues. Herein, we report the isolation, structure elucidation, and biological activities of three new sulfur containing preussomerin analogues (**1**–**3**) along with the spectroscopic characterization of the known compounds ascochytatin (**4**) and preussomerin G (**5**), all isolated from a methyl ethyl ketone extract of this strain’s culture broth. These new compounds incorporate rare thioglycolic or 2-hydroxymercaptopropionic acid moieties in their structures, which have been identified in only 17 natural products produced by fungal strains according to the Chapman and Hall Dictionary of Natural Products (accessed on 20th of January 2026), none of them belonging to the preussomerin family.^28^

## Results and Discussion

Sulphopreussomerin A (**1**) was isolated as yellow amorphous solid. The optically active compound showed low solubility in nonpolar (e.g., *n*-hexane, dichloromethane) and medium polarity (e.g., acetone) solvents. Its molecular formula was determined to be C_22_H_18_O_9_S (14 degrees of unsaturation) based on (+)-ESI-TOF measurements (*m*/*z* 476.1008 [M + NH_4_]^+^ calcd. for C_22_H_22_NO_9_S^+^ 476.1010) and the presence of 22 signals in its ^13^C NMR spectrum. The IR spectrum of **1** exhibited broad absorption bands at around 3285 cm^–1^ for hydroxy groups, at 1712 cm^–1^ for a carbonyl group, at 1611 cm^–1^ for aromatic ring and multiple absorption bands around 950–1150 cm^–1^ typically due to C–O–C or C–O stretching.
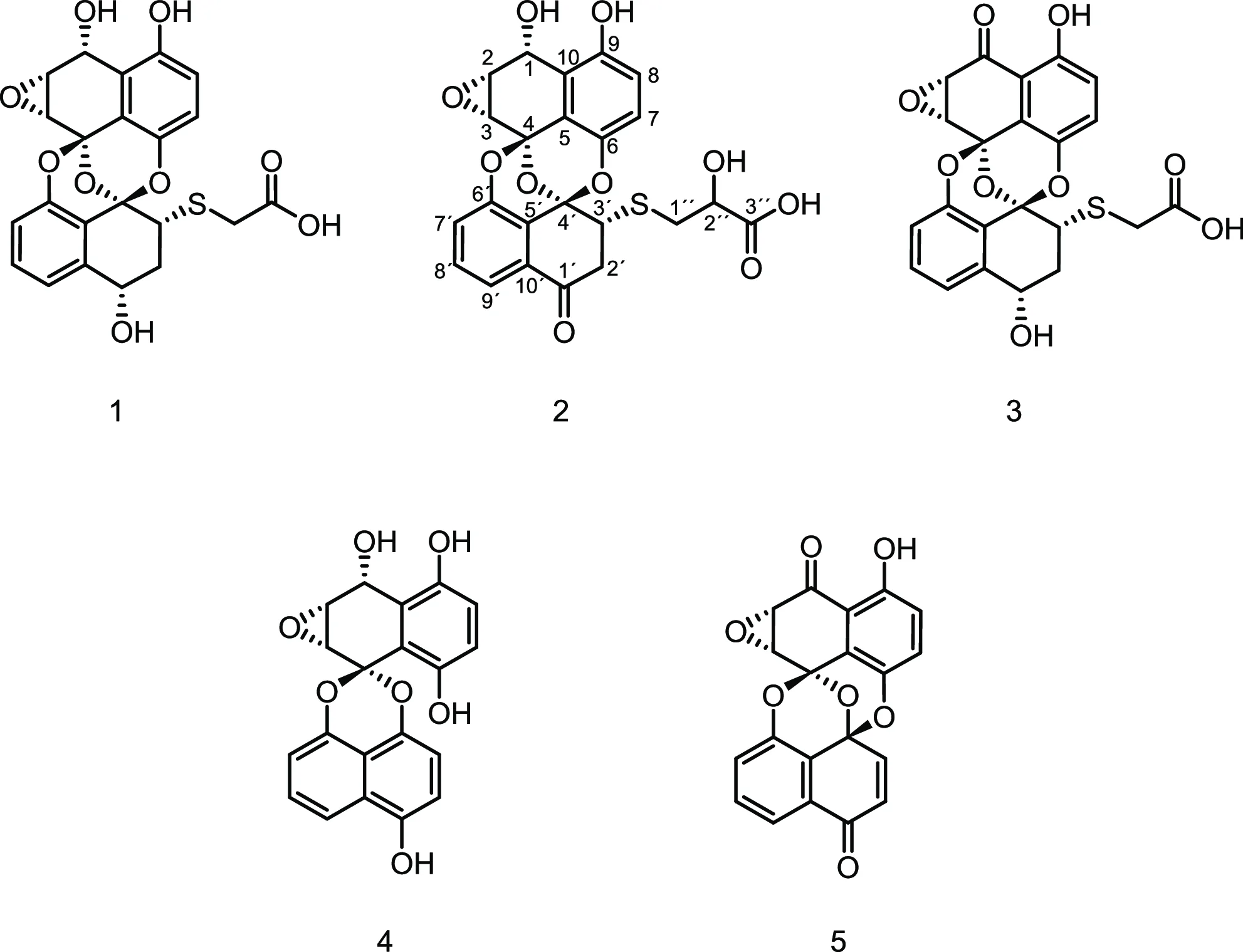


Signals observed in its ^1^H and ^13^C NMR data (Tables 1 and 2), as well as key correlations observed in the HSQC and HMBC spectra accounted for the presence of one 1,2,3-trisubstituted and one 1,2,3,4-tetrasubstituted aromatic ring, four oxygenated methines (δ_C_ 69.3, 64.9, 53.6, 53.4; δ_H_ 5.43, 4.90, 3.76, 3.98), one sulfur-bearing methine (δ_C_ 45.4; δ_H_ 3.89) and two deshielded diastereotopic methylenes (δ_C_ 36.6, 36.5; δ_H_ 3.66/3.39, 3.22/1.98). The ^13^C signals at δ_C_ 98.4, and δ_C_ 96.6 were characteristic of two ketal carbons. One signal at δ_C_ 174.2 was assigned to a carbonyl carboxylic acid group based on the molecular formula and the chemical shift. HSQC and ^13^C NMR spectra evidenced the presence of 2 methylene, 10 methine, and 10 quaternary carbons (Figures S6 and S8).

**Table 1:** ^1^H NMR Spectroscopic Data (500 MHz) for Compounds **1**–**3**

	**1** ^a^	**2** ^b^	**3** ^c^
Pos.	δ_H_, mult. (*J* in Hz)	δ_H_, mult. (*J* in Hz)	δ_H_, mult. (*J* in Hz)
1	5.43, s	5.51, s	
2	3.76, dd (4.6, 1.1)	3.87, dd (4.6, 1.1)	4.57, d (4.2)
3	3.98, d (4.6)	4.09, d (4.6)	4.04, d (4.2)
4			
5			
6			
7	6.61, d (8.9)	6.77, d (8.9)	7.05, d (9.2)
8	6.70, d (8.9)	6.81, d (8.9)	7.18, d (9.2)
9			
10			
1′	4.90^d^		4.94, dd (7.1, 3.7)
2′ a	3.22, dt (15.0, 7.1)	3.07, dd (18.5, 2.0)	3.26, dt (14.8, 7.1)
2′ b	1.98, dt (15.0, 3.7)	3.77, dd (18.5, 5.0)	2.03, dt (14.8, 3.7)
3′	3.89, dd (7.1, 3.7)	4.33, dd (5.0, 2.0)	4.05, dd (7.1, 3.7)
4′			
5′			
6′			
7′	6.71, d (7.8)	7.19, dd (7.8, 0.9)	6.87, d (7.9)
8′	7.26, t (7.8)	7.48, t (7.8)	7.41, t (7.9)
9′	7.13, d (7.8)	7.57, dd (7.8, 0.9)	7.26, d (7.9)
10′			
1″	a: 3.66, d (15.4)	a: 3.06, dd (13.8,6.2)	a: 3.71, d (15.3)
	b: 3.39, d (15.4)	b: 3.31, dd (13.8, 4.2)	b: 3.51, d (15.3)
2″		4.41, dd (6.2, 4.2)	

a CD_3_OD.

b CD_3_OD/DMSO-*d*_*6*_ (4:1).

c CD_3_OD/DMSO-*d*_*6*_ (3:2).

d The signal of H-1′ overlaps with the water signal.

**Table 2:** ^13^C NMR Spectroscopic Data (125 MHz) for **1**–**3**

	**1** ^a^	**2** ^b^	**3** ^c^
Pos.	δ_C_, type	δ_C_, type	δ_C_, type
1	69.3, CH	69.1, CH	196.0, C
2	53.6, CH	53.7, CH	54.4, CH
3	53.4, CH	53.3, CH	53.3, CH
4	96.6, C	96.9, C	95.2, C
5	115.4, C	115.0, C	112.6, C
6	145.0, C	144.3, C	145.1, C
7	117.3, CH	117.7, CH	122.3, CH
8	120.5, CH	121.0, CH	126.5, CH
9	152.1, C	152.5, C	155.2, C
10	117.8, C	118.0, C	118.0, C
1′	64.9, CH	195.0, C	64.2, CH
2′	36.5, CH_2_	42.9, CH_2_	36.5, CH_2_
3′	45.4, CH	48.9, CH	44.9, CH
4′	98.4, C	97.1, C	99.6, C
5′	117.1, C	122.1, C	116.8, C
6′	151.8, C	152.1, C	150.8, C
7′	115.3, CH	122.9, CH	115.3, CH
8′	131.7, CH	132.4, CH	132.2, CH
9′	120.4, CH	121.1, CH	121.4, CH
10′	141.0, C	131.4, C	141.9, C
1″	36.6, CH_2_	38.1, CH_2_	36.9, CH_2_
2″	174.2, C	72.1, CH	173.2, C
3″		175.7, C	

a CD_3_OD.

b CD_3_OD/DMSO-*d*_6_ (4:1).

c CD_3_OD/DMSO-*d*_6_ (3:2).

These NMR spectroscopic data indicated that compound **1** contains a spiroketal linkage. The ^13^C NMR signals at δ_C_ 98.4 and 96.6 are characteristic of ketal carbons, supporting spiroketal formation. As previously mentioned, four oxygen-bearing methines and two deshielded diastereotopic methylenes indicated an oxygen-rich environment consistent with a spiroketal system. COSY correlations established connectivity among H-1 (δ_H_ 5.43), H-2 (δ_H_ 3.76), and H-3 (δ_H_ 3.98), with H-3 showing HMBC correlation to the ketal carbon C-4 (δ_C_ 96.6). H-1 and H-3 also correlated to C-5 (δ_C_ 115.4), a quaternary aromatic carbon. The aromatic signals H-7 (δ_H_ 6.61, d, *J* = 8.9 Hz) and H-8 (δ_H_ 6.70, d, *J* = 9.0 Hz) showed an ortho coupling and an HMBC correlations to C-5, C-6 (δ_C_ 145.0), C-9 (δ_C_ 152.1), and C-10 (δ_C_ 117.8), consistent with a bicyclic naphthalene system. In the second unit, H-1′ (δ_H_ 4.90) correlated in COSY with methylene protons at C-2′ (δ_H_ 3.22/1.98), which showed HMBC correlation to C-4′ (δ_C_ 98.4). H-1′ also correlated to C-6′ (δ_C_ 151.8), and the aromatic protons H-7′ (δ_H_ 6.71), H-8′ (δ_H_ 7.26), and H-9′ (δ_H_ 7.13) displayed HMBC correlations to C-5′ (δ_C_ 117.1), C-6′, and C-10′ (δ_C_ 141.0), confirming a second bicyclic naphthalene system. Overall, these data, together with HRMS and literature comparison, supported a preussomerin-type core skeleton.^8,17^

A comparison of the NMR spectroscopic data of **1** with those of preussomerin A^17^ revealed that **1** differed from preussomerin A in the substitution at C-3′, where the double bond present in the latter compound was absent in **1**, and C-3′ was substituted with a thioglycolic acid unit. This location was confirmed since the signals for olefinic protons H-2′ and H-3′ in preussomerin A were missing in the ^1^H NMR spectrum (Table 1) of compound **1** and were replaced by a sp^3^ methine signal at δ_H_ 3.89 ppm and a sp^3^ methylene giving two signals at δ_H_ 3.22 and 1.98 ppm. Consequently, the ^13^C NMR spectrum (Table 2 and Figure S6) displayed two sp^3^ carbons with δ_C_ 45.4 ppm (CH) and δ_C_ 36.5 ppm (CH_2_) instead of two olefinic ones. Additional signals present in the ^1^H NMR of compound **1** were those corresponding to two protons of methylene C-1″ and in ^13^C NMR a carboxylic acid at C-2″ with δ_C_ 174.2 ppm that confirmed the existence of a thioglycolic acid moiety. This unit was attached to C-3′ through the sulfur atom based on key HMBC correlations observed between H-1″ and C-3′ and between H-3′ and C-1′′. An additional comprehensive analysis of UV, HRMS, 1D and 2D NMR data, including COSY and HMBC correlations (Figures 3 and S3–S11 and Table S2) allowed the full planar structure of **1** to be assigned, being the first member of the sulphopreussomerin-series.

The stereochemical analysis of sulphopreussomerin A focused on the configuration at the C-3′ stereocenter, as the stereochemistry of the molecular core had been previously established by crystallographic data for preussomerin A^17^ and was assumed to be the same in our molecule due to similar NMR chemical shifts and coupling constants. Consistent with literature data for *cis*-epoxides in naphthoquinone-derived systems, the relative configuration of the C-2/C-3 epoxide was assigned as *cis* based on the characteristic vicinal coupling constant (*J*_H‑2, H‑3_) of 4.6 Hz.^17^ The *cis*-relationship between the C-1 hydroxyl group and the epoxide was further established by a small coupling constant observed between H-1 and H-2 (1.1 Hz, seen in the H-2 signal), which results in H-1 appearing as a broad singlet (5.43 ppm). According to the Karplus relationship, this low *J*-value corresponds to a dihedral angle near 90°, a geometric hallmark of the *cis*-epoxy-alcohol moiety in the rigid preussomerin skeleton. This assignment is independently validated by NOESY correlations: H-1 exhibits a correlation to H-2 (3.76 ppm), which in turn shows a strong correlation to H-3 (3.98 ppm) (Figure S10). These correlations confirm that H-1, H-2, and H-3 all reside on the same face of the naphthalene unit, unambiguously defining the *cis* orientation of epoxide and alcohol.

The relative stereochemistry at C-3′ was first investigated by comparing experimental NMR data with two diastereomeric models, defined as epimer-a (3′*R*) and epimer-b (3′*S*) based on the previously established core.^17^ Initial structural evaluation was performed using molecular minimization with Maestro software (MacroModel, MMFFs force field), to generate these conformers (Figure S12). As discussed in the following sections, the 3′*R* assignment was subsequently confirmed as the absolute configuration by ECD analysis.

The experimentally observed vicinal coupling constant for the H2′a–H3′ spin system was 7.1 Hz. According to the Haasnoot-Altona relationship, this coupling constant corresponds to dihedral angles of approximately 42° or 133° (Figure S12).^29^ In agreement with the experinmental data, the minimized 3′*R* model of epimer-a exhibited a dihedral angle of 50°. In contrast, the minimized 3′*S* model of epimer-b displayed a dihedral angle of 175°, which would be expected to result in a significantly larger coupling constant (antiperiplanar relationship) and is therefore inconsistent with the observed *J*-value.

Additional support for the 3′*R* relative assignment was obtained from NOESY experiments. Stronger NOESY correlations were observed for H3′ and H1′ to H2′a compared to those involving H2′b, indicating that H1′ and H3′ share the same spatial orientation (Figure S13). This spatial arrangement is consistent with the 3′*R* minimized model and further excludes the 3′*S* configuration as the correct relative stereoisomer.

The absolute configuration of sulphopreussomerin A was subsequently assessed using Electronic Circular Dichroism (ECD) spectroscopy. The experimental ECD spectrum showed a negative Cotton effect at 208 nm (Δε = −85.0), followed by a positive Cotton effect with a maximum at 236 nm (Δε = +7.9). The experimental ECD spectrum of **1** (Figure 2) showed excellent agreement with the TD-DFT-calculated ECD spectrum of the (1*R*)-enantiomer. Finaly, taken together, previous crystal structure data of preussomerin A, the combined NMR spectroscopic analysis, conformational modeling, and ECD data allowed the proposal for the absolute configuration of sulphopreussomerin A to be assigned as 1*R*, 2*R*, 3*R*, 4*S*, 1′*S*, 3′*R*, 4′*S* (Figure S14).

**Figure 2:**
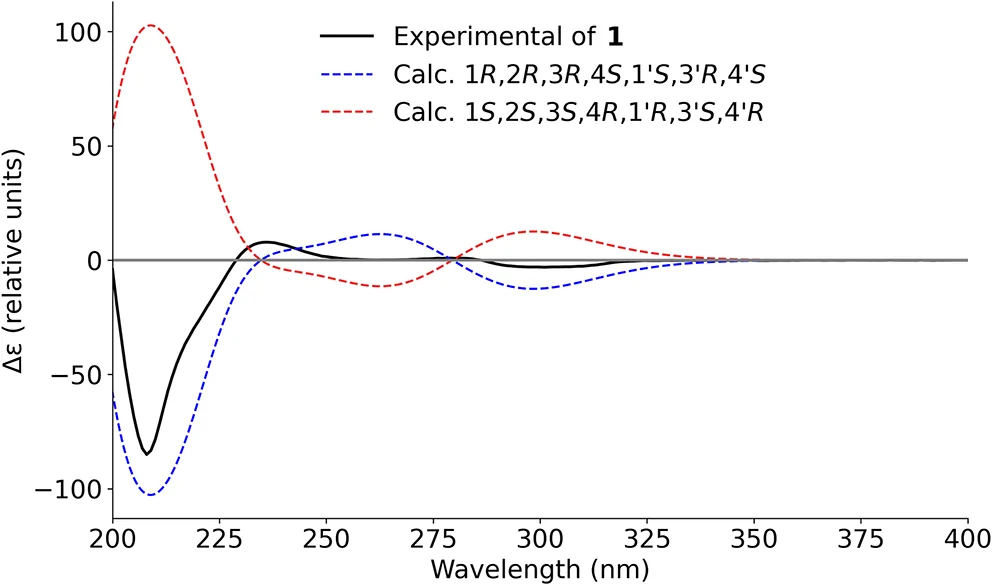
Comparative experimental and TD-DFT calculated ECD spectra for Sulphopreussomerin A (**1**).

A molecular formula of C_23_H_18_O_10_S was determined for sulphopreussomerin B (**2**), according to (+)-ESI-TOF MS (*m*/*z* 504.0954 [M + NH_4_]^+^ calcd. for C_23_H_22_NO_10_S^+^, 504.0959) and ^13^C NMR data. This formula requires 15 degrees of unsaturation, one more degree than in **1**. Sulphopreussomerin B was isolated as a light yellow amorphous solid. The molecule was found to be soluble in tetrahydrofuran, dimethyl sulfoxide, and dimethyl sulfoxide/methanol mixtures. A comparison of the NMR data of **2** (Tables 1 and 2) with those of **1** evidenced the presence of one additional oxygenated methine in the structure of **2** and the replacement of the oxygenated methine at C-1′ in **1** by a carbonyl group in **2**. The latter was evidenced in the ^13^C NMR spectrum of **2**, which displayed two carbonyl groups at δ_C_ 195.0 and 175.7, corresponding to a ketone and a carboxylic acid signal, respectively (Table 2). Key HMBC correlations from protons H-2′ and H-3′ to the carbon at δ_C_ 195.0 (Figures 3 and S22) confirmed the presence of a carbonyl group at C-1′ (Table 1). The ^1^H NMR signal of the proton in the additional oxygenated methine appears at δ_H_ 4.41 as a doublet of doublets. Correlated Spectroscopy (COSY) experiments showed a correlation between this proton with the methylene protons at C-1″ (δ_H_ 4.41 and 3.31/3.06) (Figures 3 and S20). This observation was confirmed by HMBC correlations from proton H-2″ to carbons C-3′′ and C-1′′ (δ_C_ 175.7 and 38.1) (Figure S22). Compound **2** has therefore a 2-hydroxy-3-mercaptopropionic acid unit. This unit was linked through the sulfur atom to C-3′ as confirmed by HMBC correlations between H-3′ and C-1″, and between H-1′′ and C-3′. Additional comprehensive analysis of UV, HRMS, 1D and 2D NMR data (Figures 3 and S15–S23 and Table S3) allowed the full planar structure of **2** to be assigned.

**Figure 3:**
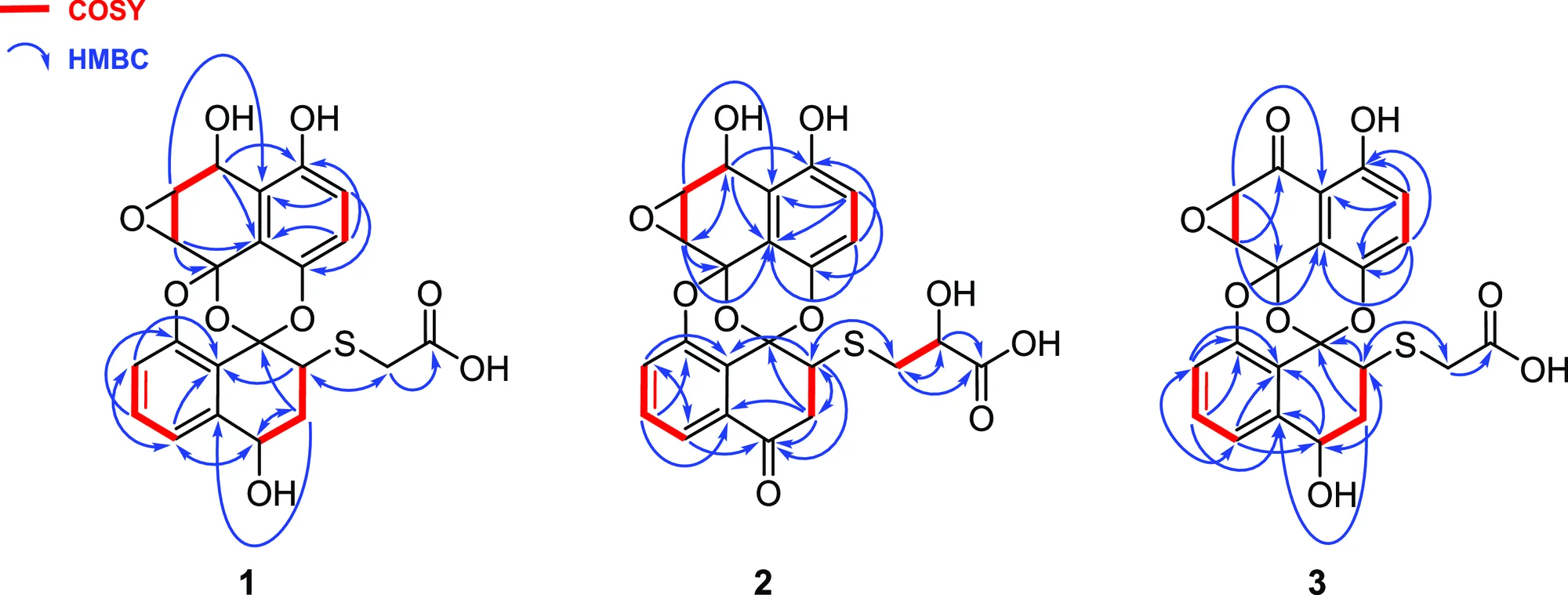
Key COSY and HMBC (H to C) correlations observed in the spectra of compounds **1**–**3**.

Compounds **1** and **2**, which possess similar C-3′ substitution patterns and exhibit closely related NMR spectroscopic characteristics, are proposed to share the same configuration at C-3′. Sulphopreussomerin B (**2**) was obtained as a single diastereomer based on NMR data. However, the absolute configuration at C-2″ remains unresolved due to scarcity of sample.

Sulphopreussomerin C (**3**) was isolated as an optically active, white amorphous solid. The assigned molecular formula was C_22_H_16_O_9_S based on the (+)-ESI-TOF spectra (*m*/*z* 474.0860 [M + NH_4_]^+^ calcd. for C_22_H_20_NO_9_S^+^, 474.0853) and 22 signals observed in ^13^C NMR data. This formula suggests that the structure has 15 double bond equivalents. A comparison of the molecular formula of **3** with that of **1** revealed the loss of two hydrogens, suggesting an oxidative transformation, likely involving the formation of a double bond or a carbonyl group. Analysis of ^13^C NMR data of **3** revealed the presence of two carbonyl groups with δ_C_ 196.0 and 173.2, which correspond to a carbonyl and a carboxylic acid signal (Table 2). COSY experiments showed that H-2 and H-3 correlated only with each other (δ_H_ 4.57 and 4.04) (Figures 3 and S28), while in the ^1^H NMR spectrum, the signal for H-1 in **1** was absent in **3** (Figure S26a). The HMBC experiment showed a correlation from H-3 to the carbon at δ_C_ 196.0, indicating that C-1 underwent an oxidation of the hydroxy group at C-1 in sulphopreussomerin A into a ketone in **3** (Figure S30). A comprehensive analysis of UV, HRMS, 1D and 2D NMR data of **3** confirmed that the rest of the structure remained the same as in compound **1** (Figure 3 and S24–S31 and Table S4). Based on their identical C-3′ substitution pattern and similar NMR chemical shifts and coupling constants, compound **3** is assumed to possess the same configuration at C-3′ as **1**.

Two previously described compounds, one of the palmarumycin type and one of the preussomerin type, were also isolated from *P. indica*. Their spectroscopic data, including ^1^H NMR and ^13^C NMR, as well as HRMS, were identical to those previously reported in the literature for ascochytatin (**4**)^30^ and preussomerin G (**5**)^31^ (Figures S32–S37).

The antifungal activities of the isolated compounds (**1**-**5**) were evaluated against four fungal phytopathogens that affect crops of economic value: *Z. tritici*, *F. proliferatum*, *C. acutatum*, and *M. grisea* (Table 3). Among the compounds tested, compound **5** exhibited significant activity, with EC_50_ values ranging from 0.31 to 0.96 μg/mL against all four fungal phytopathogens, showing activity comparable to that of the positive control, amphotericin B (AmB) (Figures S40–S41). Compounds **1** and **4** demonstrated activity specifically against *Z. tritici*: compound **1** had EC_50_ value of 20.0 μg/mL (Figure S38), whereas compound **4** showed EC_50_ value of 5.35 μg/mL in fluorescence-based assays (Figure S39). Although compound **1** displayed inhibitory activity, it did not reach maximal (100%) inhibition within the tested concentration range. Based on this information, we can infer that Sulphopreussomerin A exhibits fungistatic activity rather than fungicidal effects. No antifungal activity was observed for compounds **2** and **3** against any of the tested phytopathogens. All metabolites **1**–**5** were further evaluated for cytotoxicity using the Hep-G2 (human liver cancer) cell line, with methyl methanesulfonate (MMS) serving as the positive control (Table 3 and Figure S42). Compound **5** exhibited significant cytotoxicity against Hep-G2 cells, with a ED_50_ value of 0.68 μg/mL, whereas compounds **1**–**4** displayed weak or no cytotoxicity.

**Table 3:** Antifungal and Cytotoxic Activities (EC_50_/ED_50_, μg/mL) of Pure Compounds (**1**–**5**)­^a^

	*Z. tritici*		*F. proliferatum*		*C. acutatum*		*M. grisea*		Hep-G2
Compound name	Abs.	Fluo.	Abs.	Fluo.	Abs.	Fluo.	Abs.	Fluo.	ED_50_
Sulphopreussomerin A (**1**)	>160	**20.0**	>160	>160	>160	>160	>160	>160	>40
Sulphopreussomerin B (**2**)	>160	136.7	>160	>160	>160	>160	>160	>160	28.6 (58.8 μM)
		(127.3–146.8)							(26.3–31.1)
Sulphopreussomerin C (**3**)	>160	>160	>160	>160	>160	>160	>160	>160	**15.1** (33.1 μM)
									(14.4–15.9)
Ascochytatin (**4**)	**12.02**	**5.35**	39.12	55.31	30.0	>160	37.75	35.74	>40
	(10.63–13.59)	(4.93–5.80)	(32.21–47.51)	(50–61.19)	(21.99–40.94)		(32.25–44.19)	(32.85–38.9)	
Preussomerin G (**5**)	**0.34**	**<0.31**	**<0.31**	**<0.31**	**<0.321**	**0.96**	**0.39**	**0.33**	**0.68 (1.88 μM)**
	(0.32–0.35)	(0.29–0.32)	(0.15–0.19)	(0.18–0.21)	(0.23–0.27)	(0.88–1.05)	(0.37–0.42)	(0.32–0.33)	(0.61–0.75)
AmB	**0.21**	**0.16**	**5.43**	**5.75**	**1.35**	**0.84**	**3.17**	**3.81**	-
	(0.18–0.26)	(0.14–0.19)	(5.10–5.79)	(5.36–6.16)	(1.00–1.84)	(0.74–0.95)	(2.81–3.58)	(3.49–4.15)	

a Absorbance (Abs.; mycelial growth) and fluorescence (Fluo.; cell viability) in vitro assays. Positive controls: AmB and MMS for antifungal and cytotoxic activity, respectively. 95% confidence intervals are reported in parentheses. Bold entries indicate higher potency.

Spirodioxynaphthalenes are most frequently reported to be produced by coprophilous fungal genera, such as *Preussia, Sporormiella*, and *Chaetopreussia*.^14,32^ However, these metabolites have also been isolated from marine-derived fungi and from fungi associated with plants.^33–35^ Their occurrence across diverse environments suggests that spirodioxynaphthalene biosynthesis is not restricted to strains associated with a particular ecological niche. Instead, their distribution shows a strong association to species within the order Pleosporales; the same order to which *P. indica* belongs.^14,20,36^ In their study, Zhao et al. performed a BlastP analysis of PalA against the UniProtKB database, identifying 10 homologues exclusively within the subclass Pleosporomycetidae.^19^ The highest sequence conservation (up to 88.6%) was localized within the order Pleosporales.^19^ This strong evolutionary conservation of PalA within the Pleosporales lineage is in agreement with the documented production of spirodioxynaphthalenes across this order.

Among the palmarumycins and preussomerins isolated so far, only a few chlorinated members have been reported in both families, occurring at C-6 in palmarumycins and C-2 in both families.^11,37,38^ To date, only two palmarumycins are known to bear a sulfate group at C-9.^37^ In contrast, no sulfur-containing members of the preussomerin family have been reported so far. Herein, we present the first sulfurated members of the preussomerin family. Structure–activity relationship (SAR) analysis from this study suggests that the palmarumycin skeleton (Type A) is less potent than the preussomerin skeleton (Type B) and is also safer, as ascochytatin showed no cytotoxicity against Hep-G2 cells. As expected, Preussomerin G demonstrated broad-spectrum antifungal activity across various phytopathogens and significant cytotoxicity within similar concentration ranges. Structural analysis indicates that the C2′/C3′ double bond in preussomerin G contributes to its broad-spectrum bioactivity and high potency, suggesting that Michael addition might be involved in the mechanism of action of this molecule.^39,40^ Our data are consistent with previous findings that modifications at C-3′ in preussomerin-type compounds affect bioactivity.^23,40^ Introduction of a thioglycolic acid moiety while retaining the preussomerin core appears to reduce bioactivity. Sulphopreussomerin A showed moderate activity against *Z. tritici*, with the hydroxyl group at C-1 enhancing this effect, whereas sulphopreussomerin C, lacking this group, was completely inactive. In sulphopreussomerin B, the attachment of a 2-hydroxy-3-mercaptopropionic acid moiety to the preussomerin core does not appear to enhance antifungal activity. Importantly, despite their moderate activity, sulphopreussomerin A and ascochytatin displayed no cytotoxicity against Hep-G2 cells.

Thioglycolic and 2-hydroxymercaptopropionic acid are rare moieties in fungi, occurring in only 17 natural products.^41–43^ Compounds **1–3** could possibly be produced via Michael addition of cysteine or its thioglycolic/2-hydroxymercaptopropionic acid derivatives to the C2′/C3′ double bond of preussomerin core and thereby this resembles the glutathione-mediated detoxification pathway observed in plant and animal metabolism.^44,45^ The incorporation of thioglycolic or 2-hydroxymercaptopropionic acid moieties in compounds **1–3** results in moderate to negligible biological activity, significantly lower than that of the parent scaffold (**5**), highlighting the critical role of the C2′/C3′ double bond in mediating bioactivity. Even though this cysteine-derived pathway for **1–3** is a plausible biosynthetic hypothesis based on current knowledge, experimental validation remains to be established.

## Conclusion

Three novel spirodioxynaphthalenes, sulphopreussomerins A–C (**1**–**3**), along with the known compounds ascochytatin (**4**) and preussomerin G (**5**), were isolated from the marine-derived fungus *P. indica*. Compounds **1**–**3** represent new members of the spirodioxynaphthalene class of sulphopreussomerins; first members within preussomerin family to bear a sulfur group. Compounds **1** and **3** contain a thioglycolic acid moiety, whereas compound **2** contains a 2-hydroxy-3-mercaptopropionic acid moiety. Combined NMR analysis, including NOESY cross-peaks and vicinal coupling constants, together with electronic circular dichroism (ECD) data, allowed the assignment for the absolute stereochemistry of sulphopreussomerin A as 1*R*, 2*R*, 3*R*, 4*S*, 1′*S*, 3′*R*, 4′*S*. Among the isolated compounds, preussomerin G (**5**) exhibited a broad-spectrum antifungal activity, but it was also cytotoxic, whereas sulphopreussomerin A (**1**) and ascochytatin (**4**) showed a moderate antifungal activity against *Z. tritici* without detectable cytotoxicity. Further investigations should be conducted to elucidate the biosynthetic pathways of these compounds, specifically the enzymatic mechanisms responsible for sulfur incorporation, and to explore synthetic strategies for developing optimized scaffolds.

## Experimental Section

### General Experimental Procedures

Optical rotations were measured with a Jasco P-2000 polarimeter (JASCO Corp., Tokyo, Japan). ECD spectra were measured in acetonitrile (CH_3_CN) on a Chirascan V100 spectrophotometer (Applied Photophysics, Leatherhead, UK). IR data were obtained with a JASCO FT/IR-4100 spectrometer equipped with a PIKE MIRacle single reflection ATR accessory (JASCO Corp., Tokyo, Japan). NMR spectra were recorded at 297 K on a Bruker Avance III spectrometer (500 and 125 MHz for ^1^H and ^13^C, respectively) equipped with a 1.7 mm TCI MicroCryoProbe (Bruker Biospin, Fällanden, Switzerland). The compounds were previously dissolved in CD_3_OD, CD_3_OD/DMSO-*d*_6_ mixtures, (CD_3_)_2_CO or CDCl_3_, and their NMR chemical are reported in ppm using the signals of the residual solvents as internal reference (δ_H_ 3.31 and δ_C_ 49.15 for CD_3_OD, δ_H_ 2.50 and δ_C_ 39.52 for DMSO-*d*_6_, δ_H_ 2.05 and δ_C_ 29.84 for (CD_3_)_2_CO, δ_H_ 7.26 and δ_C_ 77.16 for CDCl_3_). Molecular models were generated using Chem & Bio Draw 12.0 (CambridgeSoft, PerkinElmer Informatics, Waltham, MA, USA).

LC-UV-MS analysis was performed on an Agilent 1260 Infinity II (Agilent Technologies, Santa Clara, CA, USA) single quadrupole LC-MS system as previously described.^46^ (+)-ESI-TOF spectra were acquired using a Bruker maXis QTOF mass spectrometer (Bruker Daltonik GmbH, Bremen, Germany) coupled to an Agilent 1200 Rapid Resolution HPLC (Agilent Technologies, Waldbronn, Germany). Medium-pressure liquid chromatography (MPLC) was performed on semiautomatic flash chromatography (CombiFlash Teledyne ISCO Rf400×) with a reversed-phase column. Semipreparative HPLC separation was performed on Gilson GX-281 322H2 (Gilson Technologies, USA) with a semipreparative reversed-phase column (Zorbax SB-C18, 9.4 mm × 250 mm, 5 μm). Preparative HPLC separation was performed on Gilson GX-281 322H2 (Gilson Technologies, USA) with reversed-phase columns (Zorbax SB-C18, 21.2 mm × 250 mm, 7 μm; X-Bridge Phenyl OBD, 19 mm × 150 mm, 5 μm; Zorbax SB-C8, 21.2 mm × 250 mm, 7 μm). Methyl ethyl ketone used for the extraction was analytical grade while all solvents employed for the isolation of the compounds were HPLC grade. In-house mixtures were used as mobile phases for chromatographic analysis, referred to hereafter as A1 and B1. Both mobile phases contain a stock modifier solution of 0.13 M trifluoroacetic acid–ammonium formate (TFA–NH_4_ formate) (10 mL TFA, 8.19 g NHA-Formate, 1 L H_2_O). The A1 mobile phase consists of 10 mL of stock modifier, 890 mL of H_2_O, and 100 mL of CH_3_CN, while the B1 mobile phase consists of 10 mL of stock modifier, 90 mL of H_2_O, and 900 mL of CH_3_CN.

### Taxonomic Identification of the Producing Microorganism

The producer strain CF-301929 was isolated from a sediment collected in the Coral Sea (Australia) following a previously described dilution to extinction method.^47^ The axenic strain was preserved as frozen suspensions of septate mycelium and conidia in 10% glycerol at −80 °C. This strain is currently maintained in the Fungal Culture Collection of Fundación MEDINA (http://www.medinadiscovery.com).

DNA extraction, PCR amplification and DNA sequencing were performed as previously described.^36^ Sequences of the complete ITS_1_-5.8S-ITS_2_ and initial 28S region or independent ITS and partial 28S rDNA were compared with those deposited at GenBank and the Westerdijk database by using the BLAST application (Table S5).^48,49^ Database matching with the ITS rDNA sequence (https://wi.knaw.nl/Pairwise_alignment or https://blast.ncbi.nlm.nih.gov/Blast.cgi) yielded a complete sequence similarity (100%) to the Ex-type strain of *P. indica* CBS124454 (GenBank Accession No. NR_160058.1) and also partial 28s yield 100% (GenBank Accession No. GQ387626.1) thus indicating that strain CF-301929 was genetically similar to *P. indica*, and conspecific.

High similar scores to other authentic fungal strains of this species, e.g., *P. indica* strain MFLUCC 24–0037 (GenBank Accession No. PQ376611.1, 100% sequence similarity),^50^ *P. indica* strain MFLUCC 24–0036 (GenBank Accession No. PQ376610.1, 99.8% sequence similarity),^50^ or *P. indica* strain MFLUCC 24–0227 (GenBank Accession No. DQ219433, 99.8% sequence similarity), indicated that CF-301929 can be classified as *P. indica*.^51^ The alignment of the complete ITS_1_-5.8S-ITS_2_ region sequences of the producer strain CF-301929, the Ex-type strain of *P. indica* CBS124454, *P. indica* strain MFLUCC 24–0037, *P. indica* strain MFLUCC 24–0036, and *P. indica* strain MFLUCC 24–0227 was performed using GeneDoc software (https://nrbsc.org/gfx/genedoc/).^52^ The alignment results are presented in Figure S43. The ITS region sequence of *P. indica* CF-301929 has been deposited in GenBank under accession number PZ210027.

### OSMAC Approach

Frozen cryotubes containing mycelia agar plugs were cultured on YM agar plates supplemented with sea salts (3%) as previously described.^53^ After 7–14 days, eight mycelial agar plugs were transferred to 16 mL of SMYA + sea salts (3%) (in g/L: Bacto neopeptone 10 g, maltose 40 g, Bacto yeast extract 10 g, agar 3.5 g) and homogenized to generate a uniform inoculum. Cultures were incubated for 7 days at 22 °C and 70% relative humidity (RH) on an orbital shaker.^53^ Production cultures were initiated with EPA vials containing four liquid media (DEX-SOY, LSFM, SMK-II, and YES supplemented with sea salts) by inoculating 0.4 mL of inoculum and were incubated for 14 days at 22 °C and 70% RH. In addition, one solid-state condition was included, a rice-based media (BRFT), where 1 mL of inoculum was added per vial and were incubated for 21 days, statically.

DEX-SOY medium contained dextrin from corn (40 g/L), glucose (10 g/L), bacto malt extract (5 g/L), polypeptone peptone (5 g/L), soybean flour (5 g/L), dipotassium phosphate (1 g/L), and bacto yeast extract (2 g/L). LSFM medium consisted of glycerol (18.7 g/L), glucose (40 g/L), tomato paste (5 g/L), yeast autolysate (5 g/L), soybean flour (5 g/L), sodium citrate (2 g/L), and ammonium sulfate (2 g/L), adjusted to pH 7.0. SMK-II medium comprised of soybean flour (1 g/L), soluble starch (40 g/L), maltose (40 g/L), Murashige and Skoog salts (4.3 g/L), and l-proline (3 g/L). YES medium contained bacto yeast extract (20 g/L), sucrose (150 g/L), magnesium sulfate heptahydrate (0.5 g/L), and 1 mL of trace elements solution (zinc sulfate heptahydrate 10 g/L and copper sulfate pentahydrate 5 g/L). All liquid media were supplemented with sea salts (3%). BRFT medium consisted of 2 g of brown rice supplemented with 11 mL of a liquid base (bacto yeast extract 1 g/L, sodium tartrate 0.5 g/L, and dipotassium phosphate 0.5 g/L) per EPA vial.

Following incubation, liquid cultures were extracted with an equal volume of acetone, by shaking at 220 rpm for 1 h. The extracts were filtered, and the organic solvent was evaporated under a nitrogen stream. The resulting filtrates were loaded onto solid phase extraction (SPE) cartridges containing 1 g of HP-20 adsorption resin, washed extensively with water, and eluted with 10 mL of acetone. The eluates were dried under a nitrogen stream. For solid-state fermentation, methyl ethyl ketone (MEK) was used as the extraction solvent and was shaken at 220 rpm for 1 h, as previously described.^54^ Extracts were concentrated under nitrogen to afford a concentration of 2 whole broth equivalents (WBE) in 20% DMSO for bioactivity screening. Media-only controls were included in each fermentation batch.

### Scale Up of Fungal Solid-State Fermentation (SSF)

A 1 L fermentation of strain CF-301929 was generated by inoculating 12 mycelial agar plugs into SMYA + sea salts (3%) in two 250 mL Erlenmeyer flasks containing 50 mL of medium. The flasks were incubated on a rotary shaker at 220 rpm, at 22 °C and 70% relative humidity. After 7 days of growth, aliquots of 8 mL of this seed culture were used to inoculate 10 × 500 mL Erlenmeyer flasks containing the production solid rice-based medium (BRFT). BRFT medium consists of 20 g of brown rice and 55 mL of a liquid base, prepared as described above, per flask. The 10 flasks were incubated under static conditions at 22 °C and 70% relative humidity for 21 days.

### Extraction and Purification of Compounds

Ten flasks that contained the solid-state fermentation of strain CF-301929 were extracted by adding methyl ethyl ketone (MEK) (10 × 100 mL) and 20 mL of HPLC grade water to each flask. Then, the flasks were shaken in a Kühner at 200 rpm for 16 h at 25 °C. After that, the extracts were centrifuged (15 min, 8000 rpm) and filtered under Büchner funnel. All solutions were pooled into a separation funnel for liquid–liquid extraction, and the organic phase was evaporated to obtain 9.4 g of a crude extract.

This crude extract was loaded onto a C-18 silica column (35 mm × 100 mm) that was eluted with a stepped H_2_O–CH_3_CN gradient (18 mL/min; 10–100% CH_3_CN in 50 min; UV detection at 210 and 280 nm) to obtain 53 fractions. Resulting fractions were tested against the fungal phytopathogen *Z. tritici* to confirm the activity, and LC/HRMS analysis led to the identification of the molecules of interest that were selected for further purification (**1**–**5**).

Fr.24 (58 mg) was further fractionated by preparative RP-HPLC (Zorbax SB-C18, 21.2 mm × 250 mm, 7 μm) applying gradient elution of H2O 0.1% TFA-CH_3_CN 0.1% TFA as mobile phase (17 mL/min; 20–30% CH_3_CN 0.1% TFA in 40 min; UV detection at 210 and 280 nm). Fractions Fr.24.42 and Fr.24.65 contained compounds **1** and **2** eluting at 25–27 min and 38–39 min, respectively. The organic solvent was evaporated under a N_2_ stream, and the resulting aqueous solution was freeze-dried. Sulphopreussomerin A (**1**) and sulphopreussomerin B (**2**) were thus obtained as yellow amorphous solids (12.5 mg and 1.7 mg, respectively).

Fr.27 (70 mg) was further fractionated by preparative RP-HPLC (X-Bridge Phenyl OBD, 19 mm × 150 mm, 5 μm) applying gradient elution of A1–B1 as mobile phase (17 mL/min; 5–100% B1 in 35 min; UV detection at 210 and 280 nm). The major peak was fraction Fr.27.26, that contained compound **4** eluting at 14.5–15.0 min. The organic solvent was evaporated under a N_2_ stream, and the resulting aqueous solution was freeze-dried. Ascochytatin (**4**) was thus obtained as a white powder (47.4 mg). Fr.27.32 (4.6 mg) was further fractionated for better separation of minor peaks by semipreparative RP-HPLC (Zorbax SB-C18, 9.4 mm × 250 mm, 5 μm) applying gradient elution of A1–B1 as mobile phase (4.2 mL/min; 30–40% B1 in 30 min; UV detection at 210 and 280 nm). Fraction Fr.27.32.24 contained compound **3** and was eluted at 13–14 min. The organic solvent was evaporated under a N_2_ stream, and the resulting aqueous solution was freeze-dried. Sulphopreussomerin C (**3**) was obtained as white amorphous solid (0.5 mg).

Fr.37 (60 mg) was further fractionated by preparative RP-HPLC (Zorbax SB-C8, 21.2 mm × 250 mm, 7 μm) applying gradient elution of H2O–CH_3_CN as mobile phase (20 mL/min; 50–100% CH_3_CN in 30 min; UV detection at 210 and 280 nm). Fraction Fr.37.19 contained compound **5** eluting at 10.5–11.5 min. The organic solvent was evaporated under a N_2_ stream, and the resulting aqueous solution was freeze-dried. Preussomerin G (**5**) was obtained as a yellow solid (6 mg).

### Characterization Data

#### Sulphopreussomerin A (**1**)

Yellow amorphous solid; [α]_D_^25^ −123.3 (*c* 0.28, MeOH); UV (DAD) v_max_ 216, 283, 300 nm; ECD (*c* 5.45 × 10^–5^ M, CH_3_CN) λ_max_ (Δε) 208 (− 85.0), 236 (+7.9) nm; IR (ATR) ν cm^–1^ 3285, 2945, 2840, 2671, 1712, 1673, 1611, 1593, 1476, 1434, 1322, 1299, 1200, 1145, 1088, 1053, 1027, 1008, 977, 952, 904, 879, 821, 798, 751. (+)-ESI-TOF *m*/*z* 934.1674 [2M + NH_4_]^+^ (calcd. for C_44_H_40_NO_18_S_2_^+^, 934.1681); *m*/*z* 459.076 [M + H]^+^ (calcd. for C_22_H_19_O_9_S^+^, 459.0744); *m*/*z* 476.1008 [M + NH_4_]^+^ (calcd. for C_22_H_22_NO_9_S^+^ 476.1010); *m*/*z* 441.0635 [M + H – H_2_0]^+^ (calcd. for C_22_H_17_O_8_S^+^, 441.0639); for ^1^H and ^13^C NMR data see Tables 1 and 2.

#### Sulphopreussomerin B (**2**)

Light yellow amorphous solid; [α]_D_^25^ −56.5 (*c* 0.04, THF); UV (DAD) v_max_ 219, 257, 301 nm; IR (ATR) ν cm^–1^ 3369, 2952, 2882, 1716, 1693, 1592, 1483, 1442, 1334, 1286, 1264, 1243, 1184, 1116, 1057, 1036, 981, 925, 849, 563, 541. (+)-ESI-TOF *m*/*z* 990.1568 [2M + NH_4_]^+^ (calcd. for C_46_H_40_NO_20_S_2_^+^, 990.1580); *m*/*z* 504.0954 [M + NH_4_]^+^ (calcd. for C_23_H_22_NO_10_S^+^, 504.0959); *m*/*z* 487.0686 [M + H]^+^ (calcd. for C_23_H_19_O_10_S^+^, 487.0693); for ^1^H and ^13^C NMR data see Tables 1 and 2.

#### Sulphopreussomerin C (**3**)

White amorphous solid; [α]_D_^25^ −105.6 (*c* 0.02, MeOH); UV (DAD) v_max_ 265, 364 nm; IR (ATR) ν cm^–1^ 3364, 2921, 2848, 1692, 1672, 1594, 1527, 1469, 1311, 1267, 1239, 1201, 1183, 1138, 1093, 1021, 951, 903, 834, 800. (+)-ESI-TOF *m*/*z* 935.0913 [2M + Na]^+^ (calcd. for C_44_H_32_NaO_18_S_2_^+^, 935.0922); *m*/*z* 930.1361 [2M + NH_4_]^+^ (calcd. for C_44_H_36_NO_18_S_2_^+^, 930.1368); *m*/*z* 474.086 [M + NH_4_]^+^ (calcd. for C_22_H_20_NO_9_S^+^, 474.0853); *m*/*z* 457.0596 [M + H]^+^ (calcd. for C_22_H_17_O_9_S^+^, 457.0588); *m*/*z* 439.0484 [M + H – H_2_0]^+^ (calcd. for C_22_H_15_O_8_S_,_^+^ 439.0482); for ^1^H and ^13^C NMR data see Tables 1 and 2.

### Computational Methods

Molecular models of compound **1** were generated using Maestro (Schrödinger, LLC), and energy minimizations were carried out with the MacroModel module using the MMFFs force field. Conformational analysis was performed in Schrodinger MacroModel (v 11.8) using a Monte Carlo Minimum Method (MCMM) and the molecular mechanics OPLS_2005 force field with an energy cut off of 5 kcal/mol and constraining the conformations using key NOESY correlations. Subsequently, conformers were fully optimized in Gaussian 16 ^55^ with a functional/basic set combination of B3LYP/6–31+G­(d,p). Then, the time-dependent density functional (TD-DFT) methodology was used to determine values of energies, oscillator strengths, and rotational strengths associated with 50 electronic excitations at the B3LYP/6–311+G­(2d,p) level. The influence of the solvent (acetonitrile) was considered within the calculations, incorporating the polarizable continuum model (PCM)^56^ with the implementation of the implicit solvation energy (IEF) approach. The resulting ECD spectra were obtained after Boltzmann averaging using Specdis 1.7.^57^ Finally, to mimic the ECD spectrum of the conformer a Gaussian function was used featuring a half-bandwidth of 0.33 eV.

### Antifungal Assays

Compounds **1**-**5** were tested for their ability to inhibit the growth of the fungal phytopathogens *Z. tritici* CBS 115943, *C. acutatum* CF-137177, *F. proliferatum* CBS 115.97 and *M. grisea* CF-105765, following previously described methodologies.^58^ Each compound was serially diluted in DMSO with a dilution factor of 2 to provide ten concentrations starting at 160 μg/mL and was dispensed in triplicate in different assay plates for all the assays. Suspensions of target microorganism conidia (1 × 10^6^ conidia/mL) were incubated with the pure compounds in RPMI-1640 medium for 24–120 h at 25 °C and 70% relative humidity (RH). The activity was assessed using resazurin, a redox indicator of cell viability, along with absorbance measurements at 600 nm, comparing initial and final incubation values to evaluate mycelial growth. EC_50_ values, representing the concentration required to achieve a half-maximal response, were determined from dose–response curves generated from both readouts. Positive control compound in the assays was amphotericin B. The Genedata Screener software (Genedata, Inc., Basel, Switzerland) was used to process and analyze the data and to calculate the RZ′ factor, which predicts the robustness of an assay.^59^ In all experiments performed in this work, the RZ′ factor obtained was between 0.87 and 0.98.

In addition, the five OSMAC-derived extracts were screened against the same panel of fungal phytopathogens using the identical assay conditions. Extracts were tested at a concentration of 2 whole broth equivalents (WBE) in 20% DMSO, and antifungal activity was expressed as percentage inhibition.

### Cytotoxicity Assays

The cytotoxicity of compounds (**1–5**) against the human liver adenocarcinoma cell line (Hep-G2, ATCC: HB-8065, RRID: CVCL_0027) was evaluated, where the in vitro cell viability was studied based on the MTT (3-(4,5-dimethylthiazol-2-yl)-2,5-diphenyltetrazolium bromide) colorimetric assay.^60^ The Hep-G2 cell line was obtained from the American Type Culture Collection (ATCC, Manassas, VA, USA). All assays were performed in triplicate, following previously described methodologies.^61^ For the preparation of the samples, each compound was serially diluted in dimethyl sulfoxide (DMSO) with a dilution factor of 2 to provide 10 concentrations starting at 80 μg/mL. Briefly, cells were seeded at 10,000 cells/well in a 96-well plate (Corning 96 Well TC-Treated Microplates). After 24 h, cells were treated with compounds for 72 h. MMS (methylmethanesulfonate, Sigma-Aldrich) 2 mM was used as positive control of cell death. Then, MTT dye (Thiazolyl blue tetrazolium bromide, ACROS Organics) was added for 2 h and then absorbance was measured at 570 nm. Data obtained were analyzed using Genedata Screener Software.

## Supplementary Material



## Data Availability

The NMR data for sulphopreussomerins A-C (**1–3**) have been deposited in the Natural Products Magnetic Resonance Database (NPMRD; www.npmrd.org). The accession numbers are NP0353882 for sulphopreussomerin A (**1**), NP0353883 for sulphopreussomerin B (**2**), and NP0353884 for sulphopreussomerin C (**3**). The ITS region sequence of *P. indica* CF-301929 is available at GenBank (Accession No: PZ210027).

## Notes

Additional information on strain CF-301929 cultivation, bioactivity, ITS region, UV, HRESIMS, 1D and 2D NMR spectra of the isolated compounds, and bioactivity dose–response curves (PDF)
