# Supplementary material for: Sulphopreussomerins: Sulfur-Containing Preussomerins Isolated from the Marine-Derived Fungus Pyrenochaetopsis indica
Source: J Nat Prod. 2026 May 15;89(8):2253–64. doi: 10.1021/acs.jnatprod.6c00324 (PMC13532173; doi:10.1021/acs.jnatprod.6c00324)
Supplement: Supplementary file 1 [file np6c00324_si_001.pdf]

SUPPORTING INFORMATION

Sulphopreussomerins: Sulfur-Containing Preussomerins Isolated from the Marine-Derived Fungus *Pyrenochaetopsis indica*

Georgia Karyofyllidou[1]*, Rachel Serrano[1], Víctor González-Menéndez[1], Jesús Martín[1], Thomas A. Mackenzie[1], Maria C. Ramos[1], Juan Ortega-Vidal[2], Olivier P. Thomas[2], José R. Tormo[1], Olga Genilloud[1], Fernando Reyes[1]*

[1]*Fundación MEDINA, Centro de Excelencia en Investigación de Medicamentos Innovadores en Andalucía, Avda. del Conocimiento 34, Parque Tecnológico de Ciencias de la Salud, 18016, Armilla, Granada, Spain*

[2] *School of Biological and Chemical Sciences, Ryan Institute, University of Galway, H91TK33, Galway, Ireland*

Corresponding Authors

*G. Karyofyllidou, Tel.: +30 6981798788, E-mail: georgia.karyofyllidou@medinaandalucia.es

*F. Reyes, Tel.: +34 677092805, E-mail: fernando.reyes.benitez@gmail.com

Table of Contents

Figure S1. Stacked HPLC chromatograms (fingerprints) of strain CF-301929 extracts cultivated across five different media, detected at a UV wavelength of 210 nm (samples diluted in 20% DMSO)........ 5

Table S1. Antimicrobial activity (% inhibition) of extracts of strain CF-301929 across five different media against *Z. tritici, F. proliferatum, C. acutatum* and *M. grisea* based on absorbance (mycelia development) and fluorescence (cell viability) in vitro assays with Amphotericin B as positive control........ 6

Figure S2. Strain CF-301929 cultivated in a solid-state rice-based medium (BRFT) in an Erlenmeyer flask for 21 days. 7

Figure S3. DAD UV-vis spectrum of sulphopreussomerin A (**1**) in acetonitrile (ACN). 8

Figure S4a. HR ESI-TOF MS spectrum of sulphopreussomerin A (**1**)........ 9

Figure S4b. Expansion of HR ESI-TOF MS spectrum of sulphopreussomerin A (**1**) 9

Table S2. NMR spectroscopic data of sulphopreussomerin A (**1**)........ 10

Figure S5a. ^{1}H NMR spectrum of sulphopreussomerin A (**1**) (CD_3OD, 500 MHz)........11

Figure S5b. Expansion of ^{1}H NMR spectrum of sulphopreussomerin A (**1**) (CD_3OD, 500 MHz) 12

Figure S5c. Expansion of ^{1}H NMR spectrum of sulphopreussomerin A (**1**) (CD_3OD, 500 MHz) 12

Figure S6. ^{13}C NMR spectrum of sulphopreussomerin A (**1**) (CD_3OD, 125 MHz)........ 13

Figure S7. COSY spectrum of sulphopreussomerin A (**1**) 14

Figure S8. HSQC spectrum of sulphopreussomerin A (**1**)........ 15

Figure S9. HMBC spectrum of sulphopreussomerin A (**1**)........ 16

Figure S10. NOESY spectrum of sulphopreussomerin A (**1**)........ 17

Figure S11. TOCSY spectrum of sulphopreussomerin A (**1**) 18

Figure S12. Maestro-minimized structures of epimer-b (3′*S*, top left) and epimer-a (3′*R*, top right) sulphopreussomerin A (**1**) using MacroModel and the MMFFs force field. (Bottom) Comparison table of calculated dihedral angles versus experimental coupling constants (*J* in Hz). 19

Figure S13. Expansion of the NOESY spectrum (left) of sulphopreussomerin A (**1**), showing the same pattern between H3′-H2′a-H2′b and H1′-H2′a-H2′b, with a more intense signal for H2′a (3.22 ppm) and a less intense signal for H2′b (1.98 ppm), supporting a common orientation of H1′ and H3′. This observation is consistent with the Maestro-minimized 3′*R* structure (epimer-a) of sulphopreussomerin A (**1**) (right)........ 20

Figure S14. Overlaid 3D structures for 1*R*, 2*R*, 3*R*, 4*S*, 1′*S*, 3′*R*, 4′*S*-Sulphopreussomerin A conformers. 21

Figure S15. DAD UV-vis spectrum of sulphopreussomerin B (**2**) in methanol (MeOH)............ 22
Figure S16. HR ESI-TOF MS spectrum of sulphopreussomerin B (**2**) 22
Table S3. NMR spectroscopic data of sulphopreussomerin B (**2**) 23
Figure S17a. ^{1}H NMR spectrum of sulphopreussomerin B (**2**) (CD_3OD/DMSO-d_6, 4:1, 500 MHz).. 24
Figure S17b. Expansion of ^{1}H NMR spectrum of sulphopreussomerin B (**2**) (CD_3OD/DMSO-d_6, 4:1, 500 MHz).. 25
Figure S17c. Expansion of ^{1}H NMR spectrum of sulphopreussomerin B (**2**) (CD_3OD/DMSO-d_6, 4:1, 500 MHz).. 25
Figure S18. *J*-Resolved spectrum of sulphopreussomerin B (**2**) (CD_3OD/DMSO-d_6, 4:1, 500 MHz).. 26
Figure S19. ^{13}C NMR spectrum of sulphopreussomerin B (**2**) (CD_3OD/DMSO-d_6, 4:1, 125 MHz).. 27
Figure S20. COSY spectrum of sulphopreussomerin B (**2**) 28
Figure S21. HSQC spectrum of sulphopreussomerin B (**2**)... 29
Figure S22. HMBC spectrum of sulphopreussomerin B (**2**).. 30
Figure S23. NOESY spectrum sulphopreussomerin B (**2**)... 31
Figure S24. DAD UV-vis spectrum of sulphopreussomerin C (**3**) in methanol (MeOH)............ 32
Figure S25a. HR ESI-TOF MS spectrum of sulphopreussomerin C (**3**) 33
Figure S25b. Expansion of HR ESI-TOF MS spectrum of sulphopreussomerin C (**3**)............... 33
Table S4. NMR spectroscopic data of sulphopreussomerin C (**3**) 34
Figure S26a. ^{1}H NMR spectrum of sulphopreussomerin C (**3**) **(**CD_3OD/DMSO-d_6, 3:2, 500 MHz).. 35
Figure S26b. Expansion of ^{1}H NMR spectrum of sulphopreussomerin C (**3**) **(**CD_3OD/DMSO-d_6, 3:2, 500 MHz).. 36
Figure S26c. Expansion of ^{1}H NMR spectrum of sulphopreussomerin C (**3**) **(**CD_3OD/DMSO-d_6, 3:2, 500 MHz 36
Figure S27. ^{13}C NMR spectrum of sulphopreussomerin C (**3**) (CD_3OD/DMSO-d_6, 3:2, 125 MHz).. 37
Figure S28. COSY spectrum of sulphopreussomerin C (**3**) 38
Figure S29. HSQC spectrum of sulphopreussomerin C (**3**)... 39
Figure S30. HMBC spectrum of sulphopreussomerin C (**3**).. 40
Figure S31. NOESY spectrum of sulphopreussomerin C (**3**) 41
Figure S32. ^{1}H NMR spectrum of ascochytatin (**4**) ($(CD_3)_2CO$, 500 MHz)................................ 42
Figure S33. ^{13}C NMR spectrum of ascochytatin (**4**) ($(CD_3)_2CO$, 125 MHz)............................... 42

Figure S34. HR ESI-TOF MS spectrum of ascochytatin (**4**) 43

Figure S35. ^{1}H NMR spectrum of preussomerin G (**5**) ($CDCl_3$, 500 MHz) 43

Figure S36. ^{13}C NMR spectrum of preussomerin G (**5**) ($CDCl_3$, 125 MHz) 44

Figure S37. HR ESI-TOF MS spectrum of preussomerin G (**5**) 44

Figure S38. Dose-response curves of sulphopreussomerin A (**1**) against *Z. tritici* based on absorbance and fluorescence in vitro assays 45

Figure S39. Dose-response curve of ascochytatin (**4**) against *Z. tritici* based on absorbance and fluorescence in vitro assays 46

Figure S40. Dose-response curves of preussomerin G (**5**) against a) *Z. tritici* and b) *F. proliferatum* based on absorbance and fluorescence in vitro assays 47

Figure S41. Dose-response curves of preussomerin G (**5**) against a) *C. acutatum* and b) *M. grisea* based on absorbance and fluorescence in vitro assays.. 48

Figure S42. Dose-response curves of compounds **1**-**5** against the Hep-G2 human cell line in vitro assays.. 49

Table S5. Sequence of the ITS region of the fungus *Pyrenochaetopsis indica*........................... 50

Figure S43. Alignment of the complete ITS_1-5.8S-ITS_2 region sequences of the producer strain CF-301929, the Ex-type strain of *Pyrenochaetopsis indica* CBS124454*, P. indica* strain MFLUCC 24-0037, *P. indica* strain MFLUCC 24-0036, and *P. indica* strain MFLUCC 24-0227 performed by GeneDoc software 51

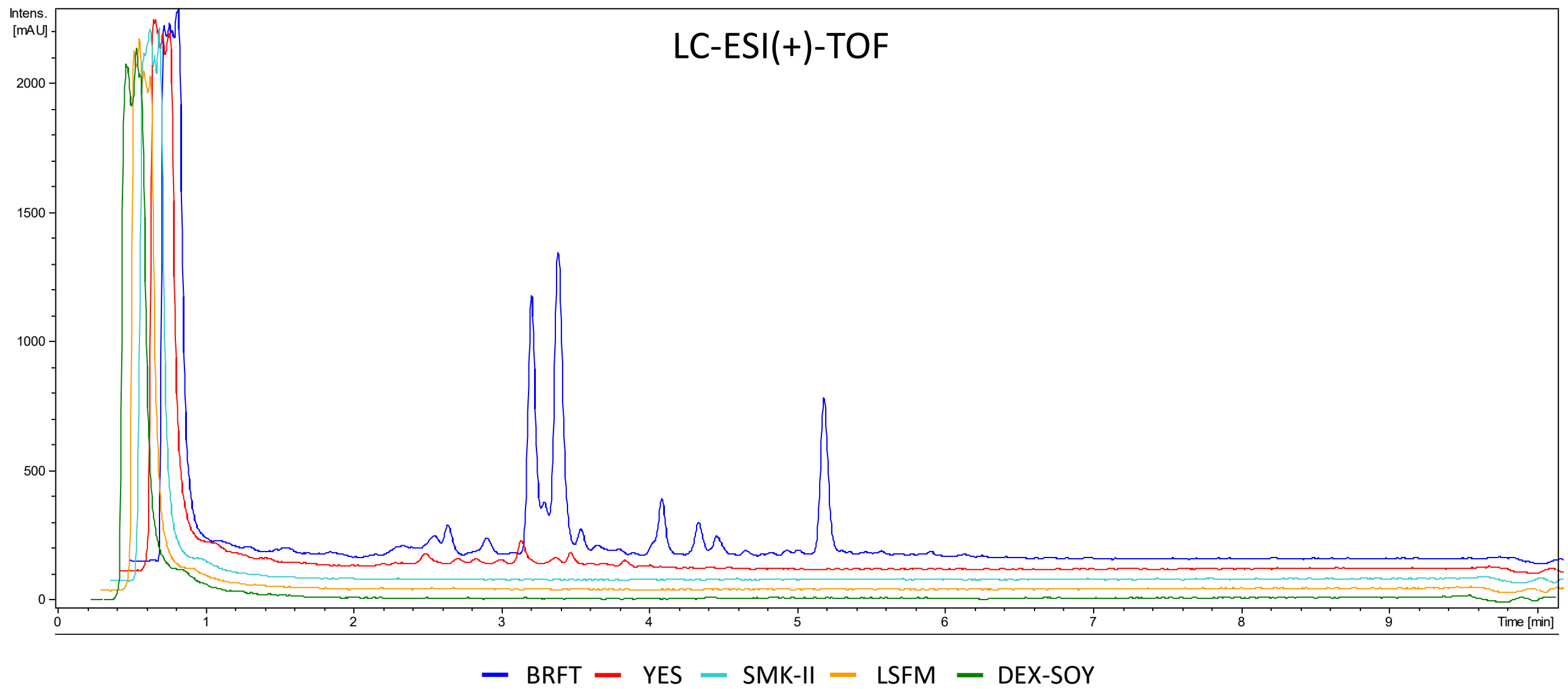

Figure S1. Stacked HPLC chromatograms (fingerprints) of strain CF-301929 extracts cultivated across five different media, detected at a UV wavelength of 210 nm (samples diluted in 20% DMSO)

		Z. tritici		*C. acutatum*		*F. proliferatum*		*M. grisea*	
		**% INH Absorbance**	**% INH Fluorescence**	**% INH Absorbance**	**% INH Fluorescence**	**% INH Absorbance**	**% INH Fluorescence**	**% INH Absorbance**	**% INH Fluorescence**
**CF-301929**	**DEX-SOY**	0	54	0	40	0	0	0	0
	**LSFM**	14	0	0	0	0	0	0	0
	**SMK-II**	36	0	0	0	0	0	0	0
	**YES**	22	0	0	0	0	0	0	1
	**BRFT**	98	105	72	104	75	101	76	102
**Amphotericine B**		100	99	101	98	105	100	94	100

Table S1. Antimicrobial activity (% inhibition) of extracts of strain CF-301929 across five different media against *Z. tritici, F. proliferatum, C. acutatum* and *M. grisea* based on absorbance (mycelia development) and fluorescence (cell viability) in vitro assays with Amphotericin B as positive control

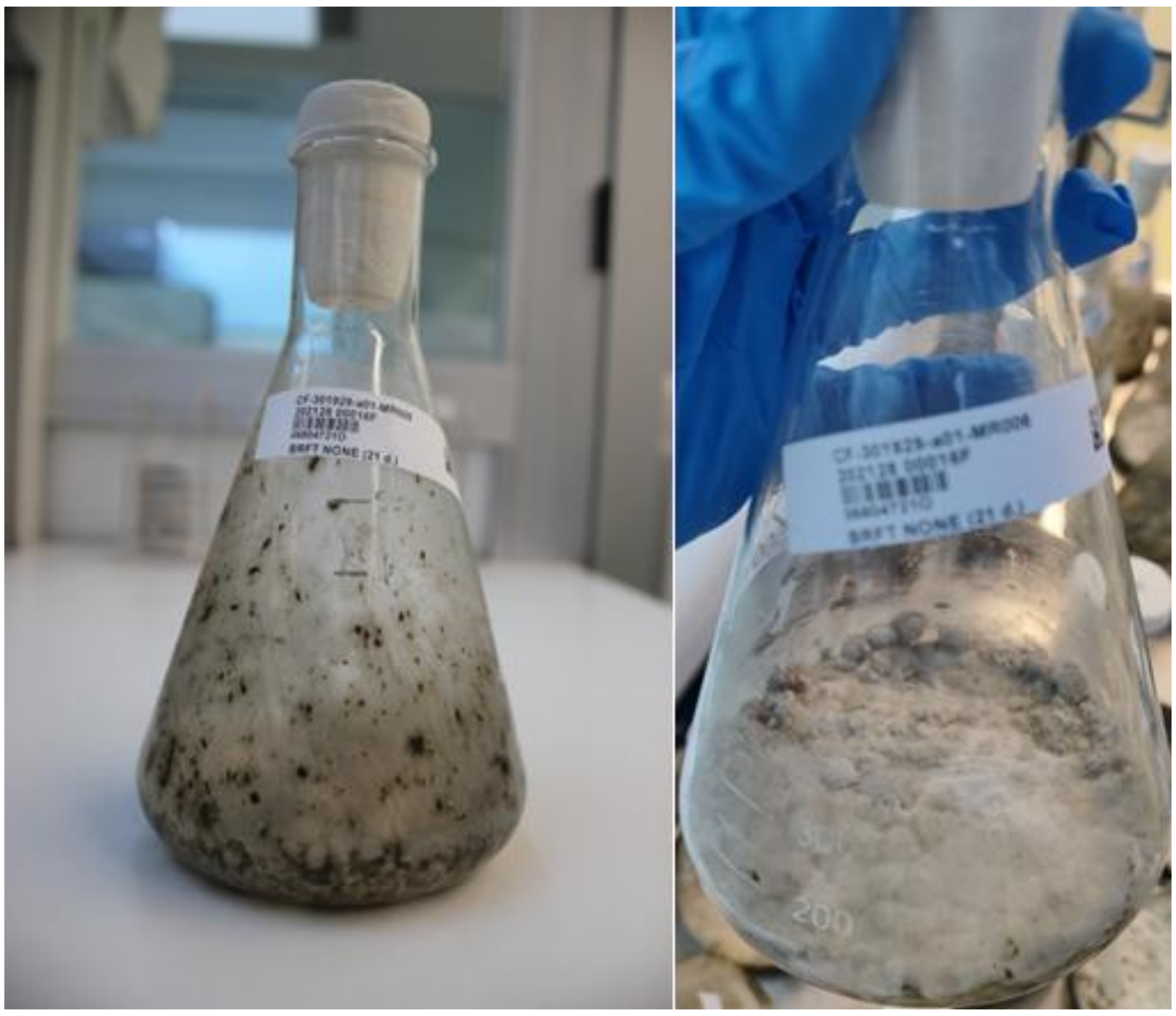

Figure S2. Strain CF-301929 cultivated in a solid-state rice-based medium (BRFT) in an Erlenmeyer flask for 21 days.

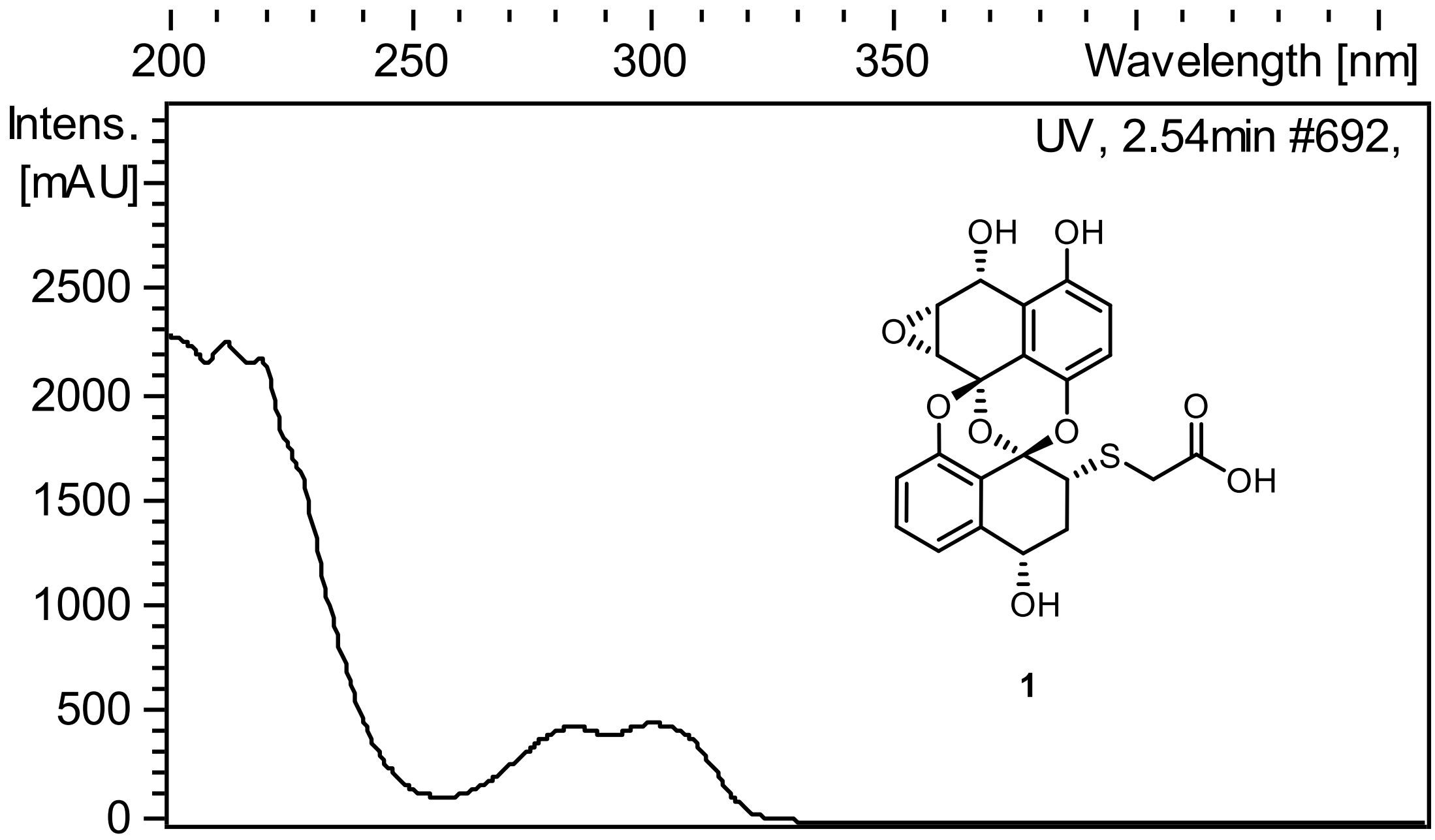

Figure S3. DAD UV-vis spectrum of sulphopreussomerin A (**1**) in acetonitrile (ACN).

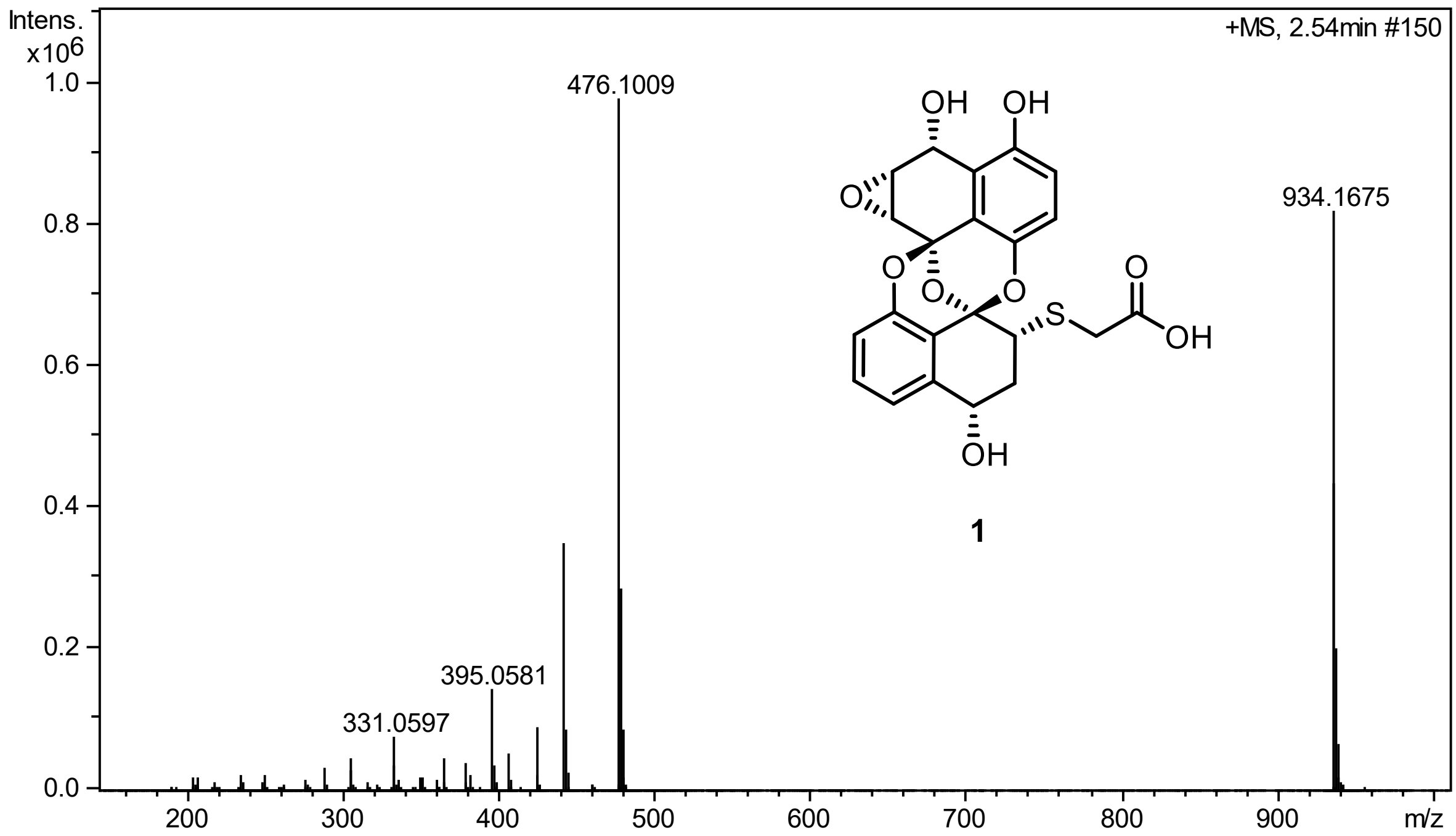

Figure S4a. HR ESI-TOF MS spectrum of sulphopreussomerin A (**1**)

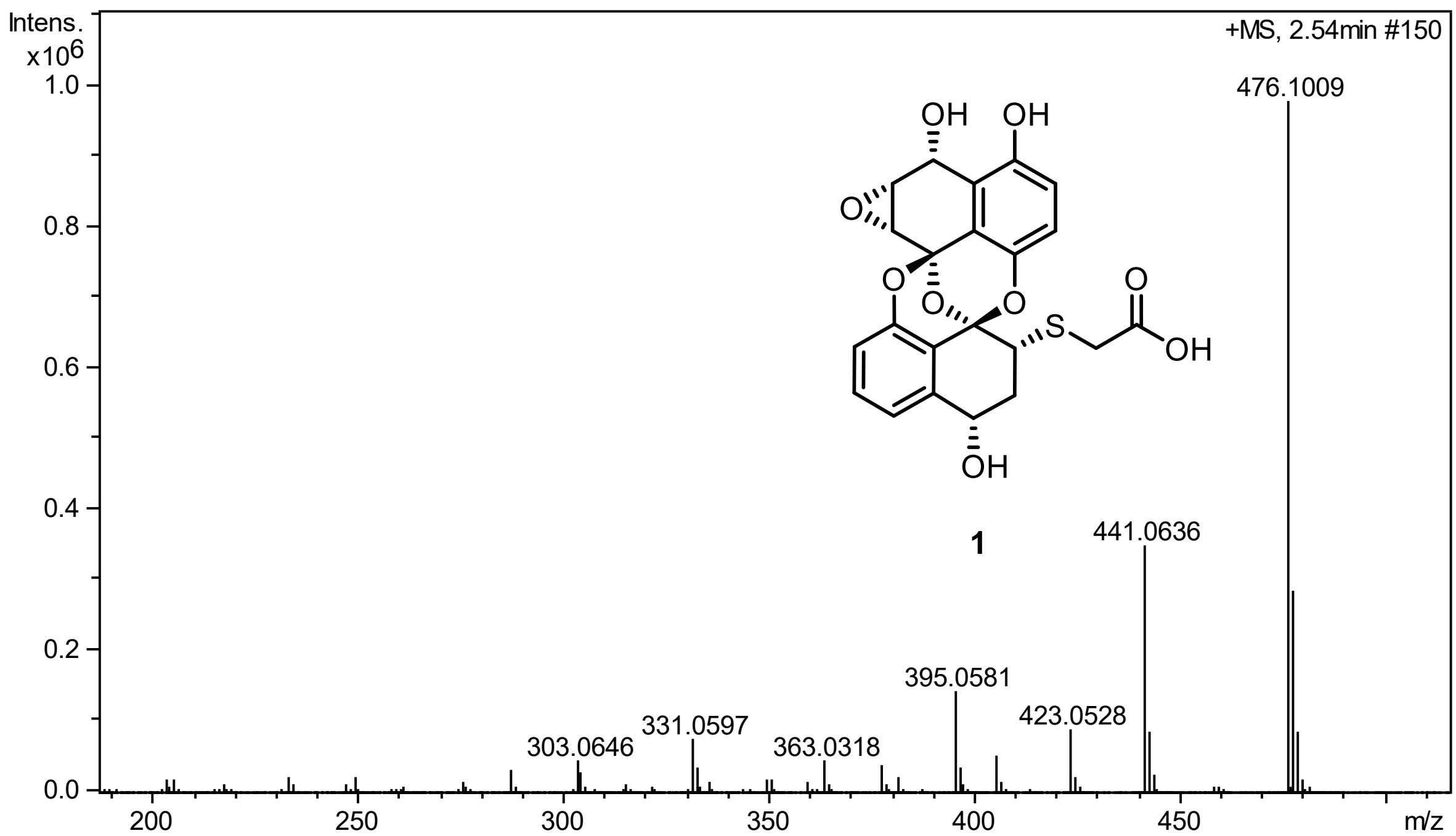

Figure S4b. Expansion of HR ESI-TOF MS spectrum of sulphopreussomerin A (**1**)

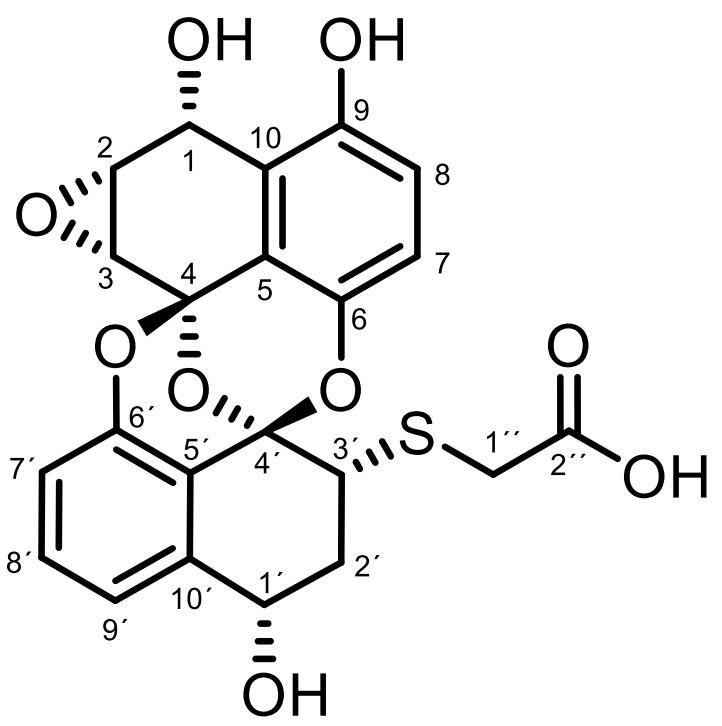

Table S2. NMR spectroscopic data of sulphopreussomerin A (**1**)

Pos.	δ_C, type	δ_H, mult. (J in Hz)	COSY	HMBC (H to C)	TOCSY	NOESY
1	69.3, CH	5.43, s	2	5, 9, 10	2, 3	2
2	53.6, CH	3.76, dd (4.6, 1.1)	1, 3	1, 3, 9, 10	1, 3	1,3
3	53.4, CH	3.98, d (4.6)	2	2, 4, 5	1, 2	2
4	96.6, C					
5	115.4, C					
6	145.0, C					
7	117.3, CH	6.61, d (8.9)	8	5, 6, 9	8	
8	120.5, CH	6.70, d (8.9)	7	6, 9, 10	7	
9	152.1, C					
10	117.8, C					
1′	64.9, CH	4.90*	2′	2′, 3′, 5′, 9′, 10′	2′, 3′	2′, 9′
2′	36.5, CH_2	a: 3.22, dt (15.0, 7.1)	1′, 2′ b, 3′	1′, 3′, 4′, 10′	1′, 2′b, 3′	3′, 1′, 2′ b
		b: 1.98, dt (15.0, 3.7)	1′, 2′ a, 3′		1′, 2′a, 3′	3′, 1′, 2′ a
3′	45.4, CH	3.89, dd (7.1, 3.7)		1′, 4′, 5′, 1″		2′, 1″
4′	98.4, C					
5′	117.1, C					
6′	151.8, C					
7′	115.3, CH	6.71, d (7.8)	8′	5′, 9′	8′, 9′	8′
8′	131.7, CH	7.26, t (7.8)	7′, 9′	6′, 10′	9′	7′, 9′
9′	120.4, CH	7.13, d (7.8)	8′	1′, 5′, 7′	8′	1′, 8′
10′	141.0, C					
1″	36.6, CH_2	a: 3.66, d (15.4)	1″b	3′, 2″	1″b	1″b
		b: 3.39, d (15.4)	1″a		1″a	1″a
2″	174.2, C					

* The signal of H-1′ overlaps with the water signal.

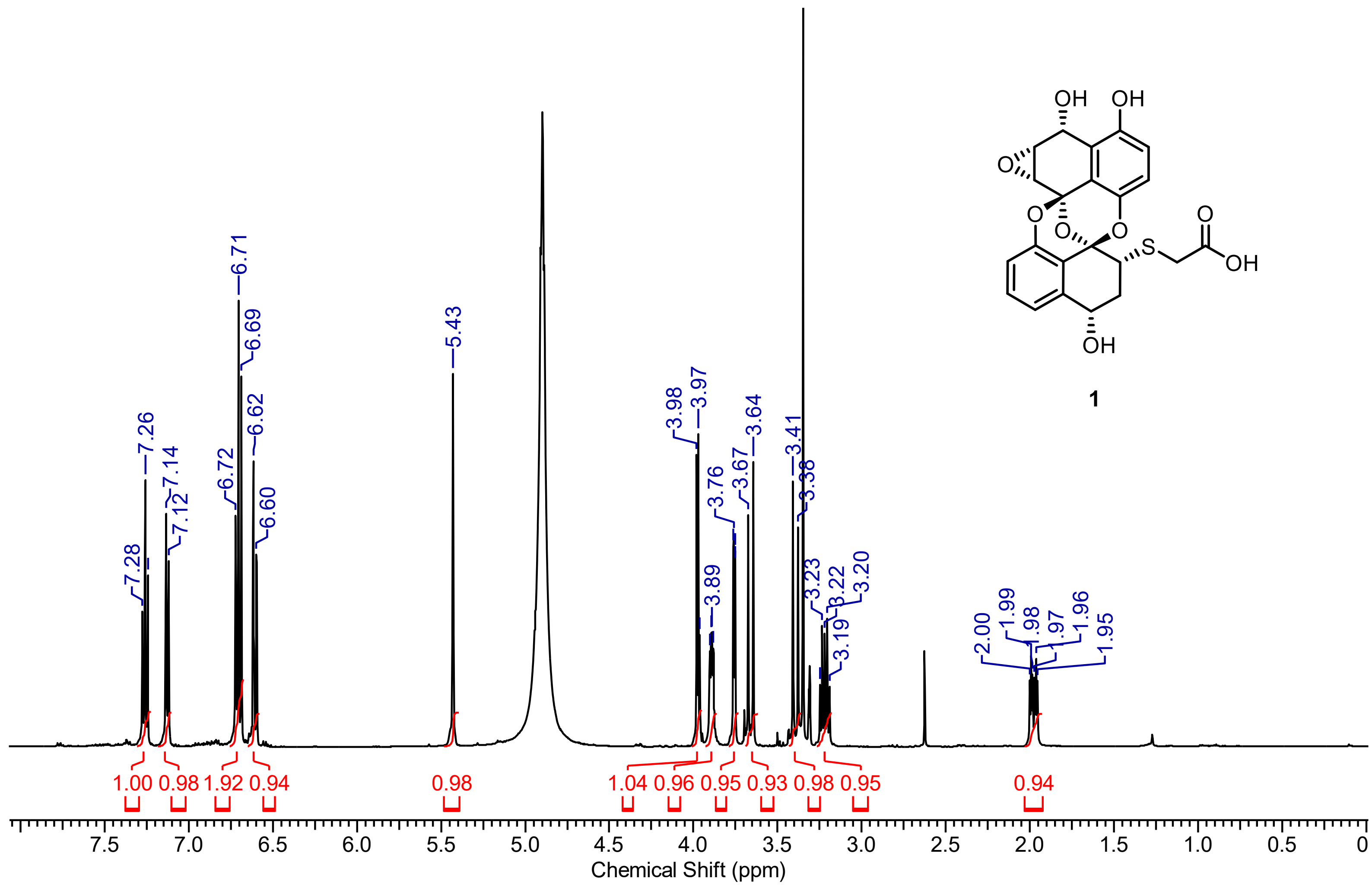

Figure S5a. ^{1}H NMR spectrum of sulphopreussomerin A (**1**) (CD_3OD, 500 MHz)

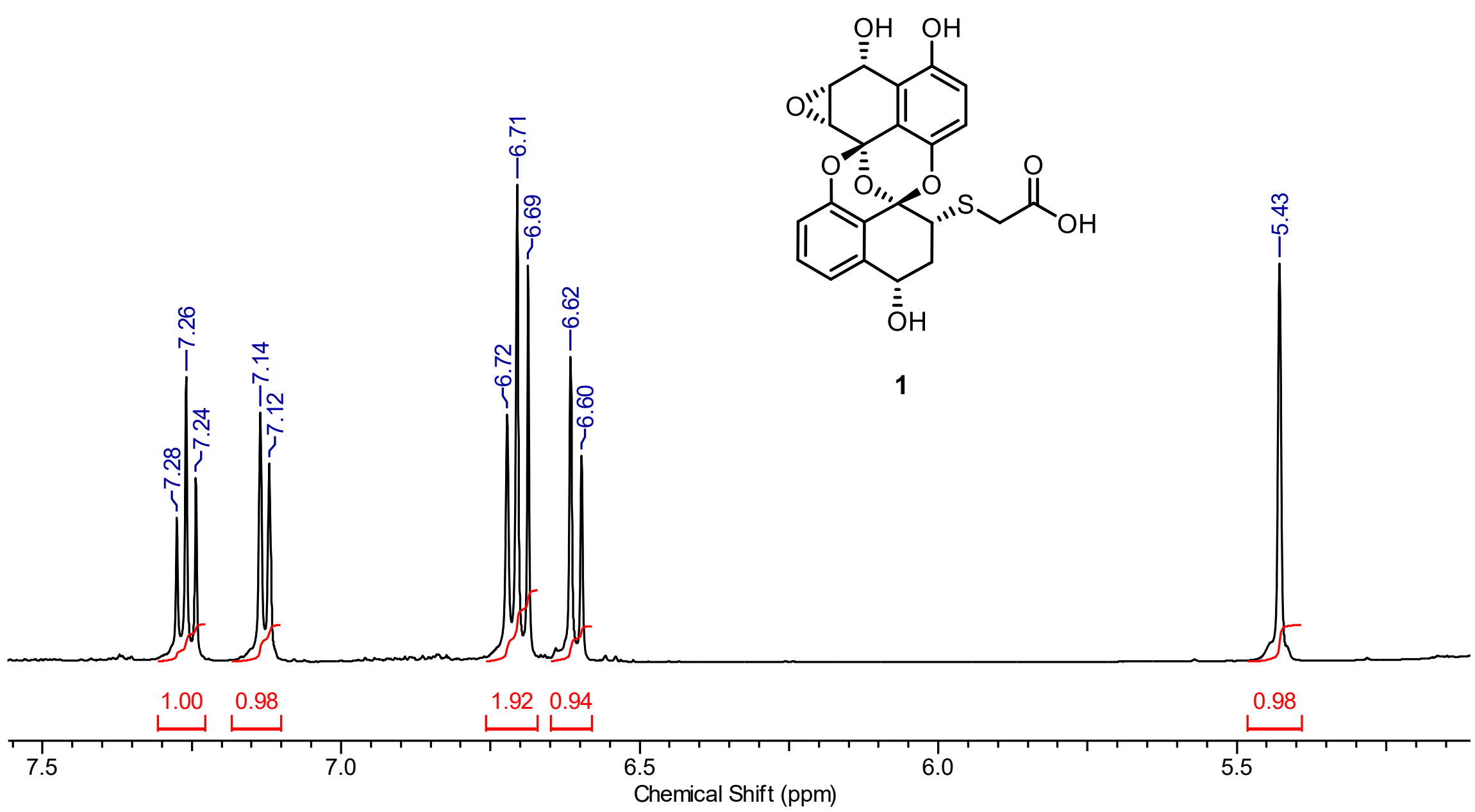

Figure S5b. Expansion of ^{1}H NMR spectrum of sulphopreussomerin A (**1**) (CD_3OD, 500 MHz)

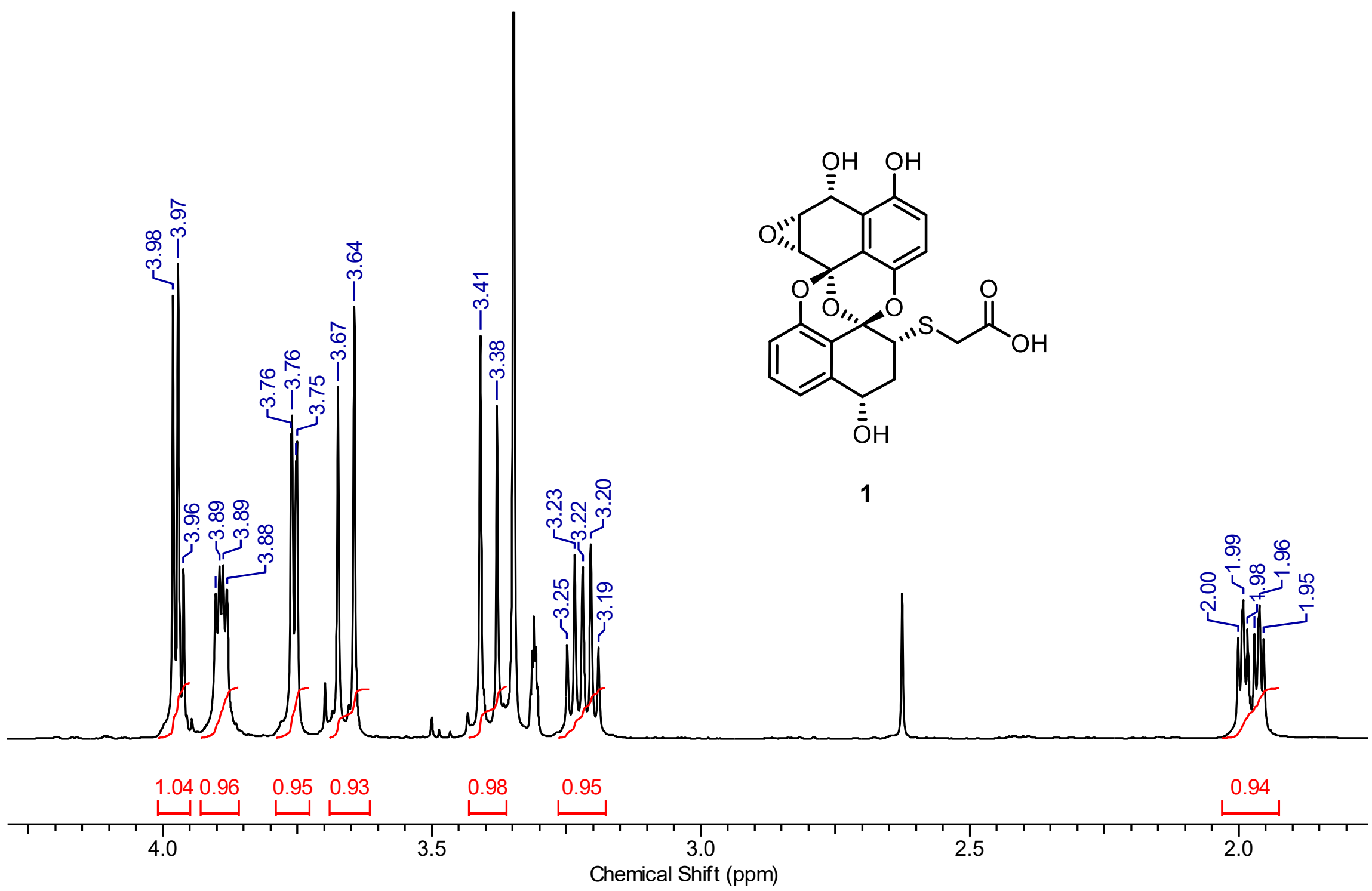

Figure S5c. Expansion of ^{1}H NMR spectrum of sulphopreussomerin A (**1**) (CD_3OD, 500 MHz)

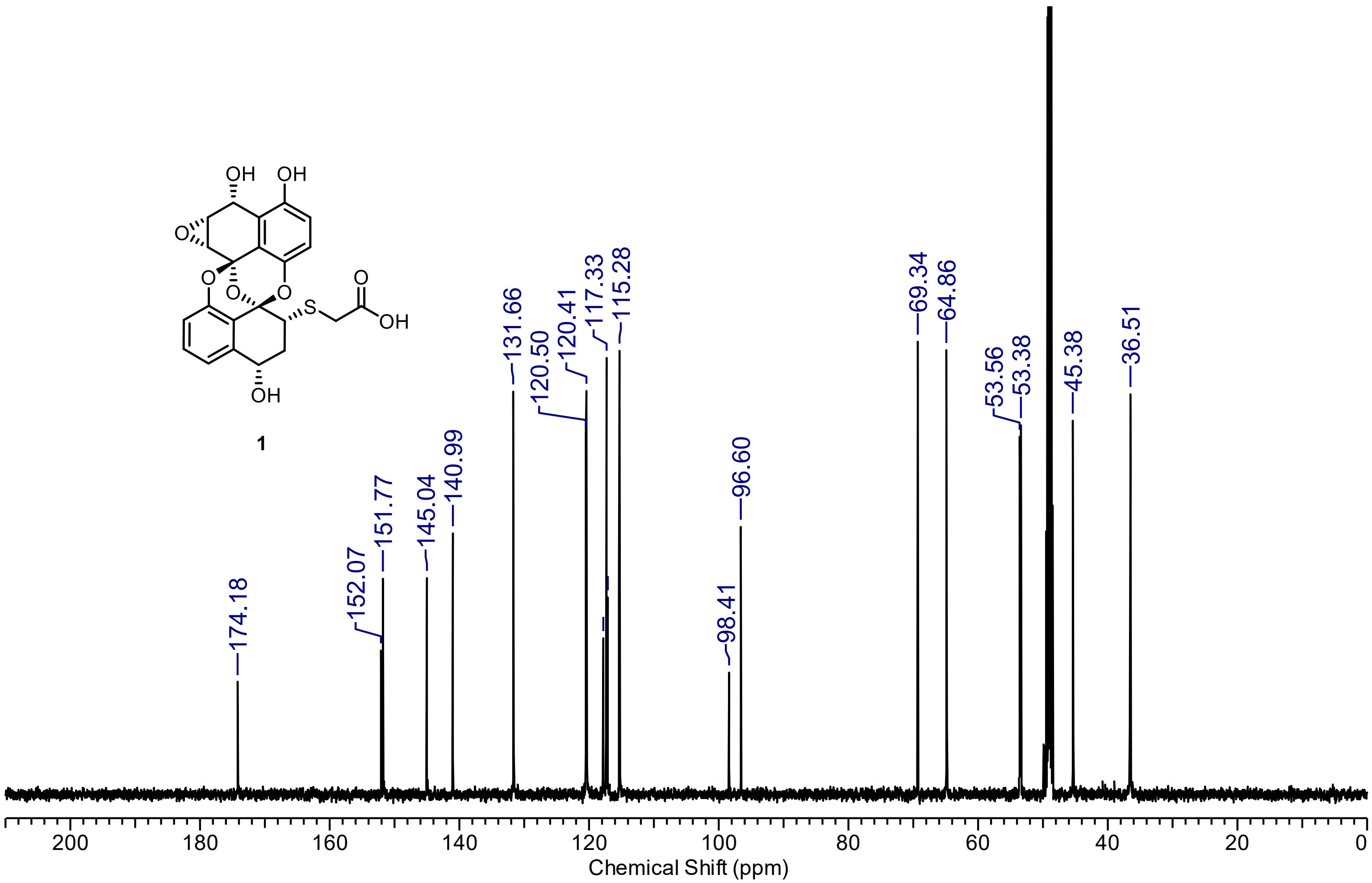

Figure S6. ^{13}C NMR spectrum of sulphopreussomerin A (**1**) (CD_3OD, 125 MHz)

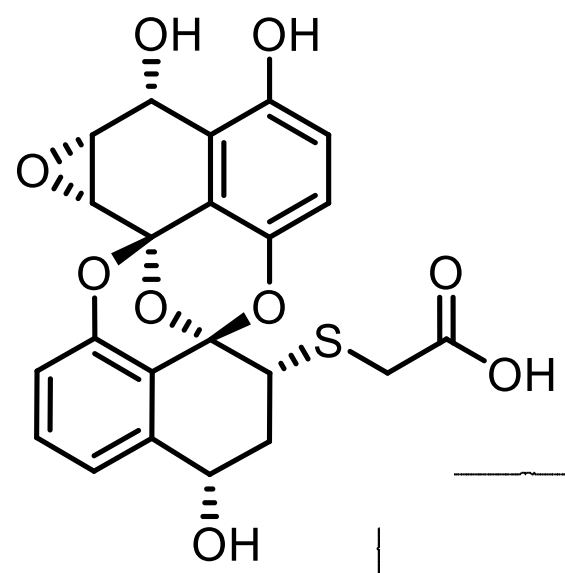

1

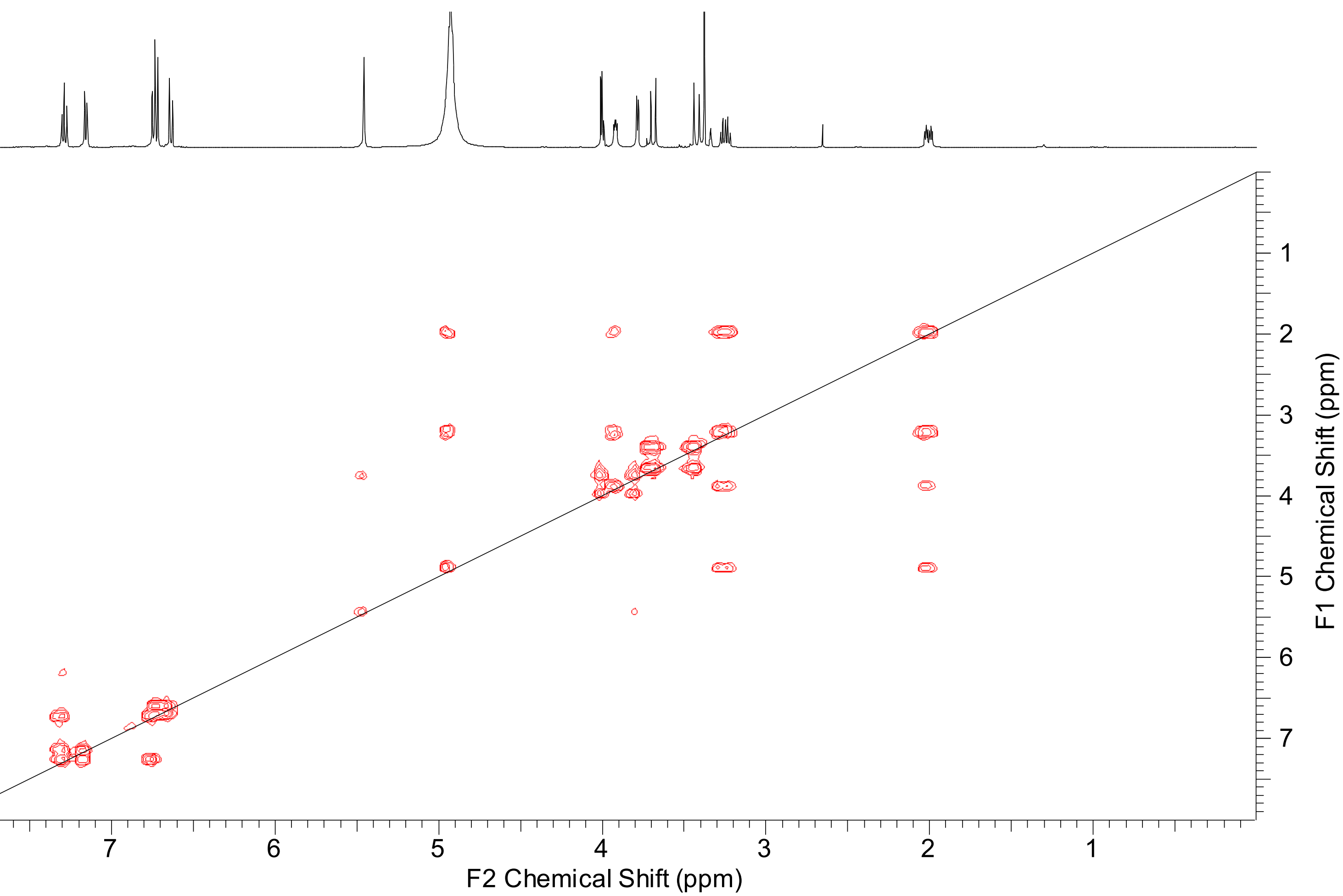

Figure S7. COSY spectrum of sulphopreussomerin A (**1**)

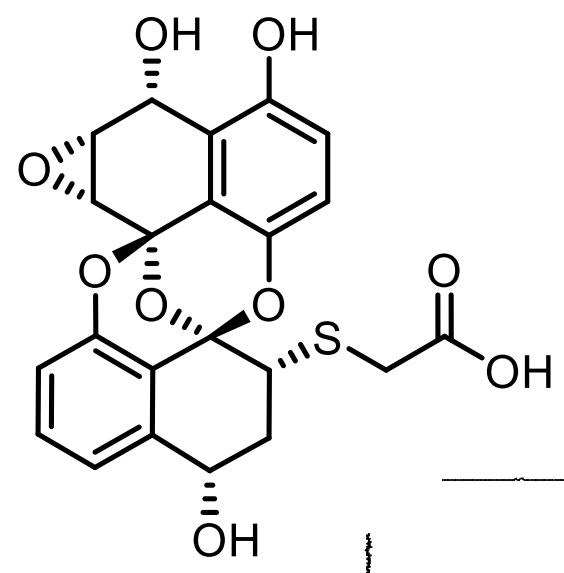

1

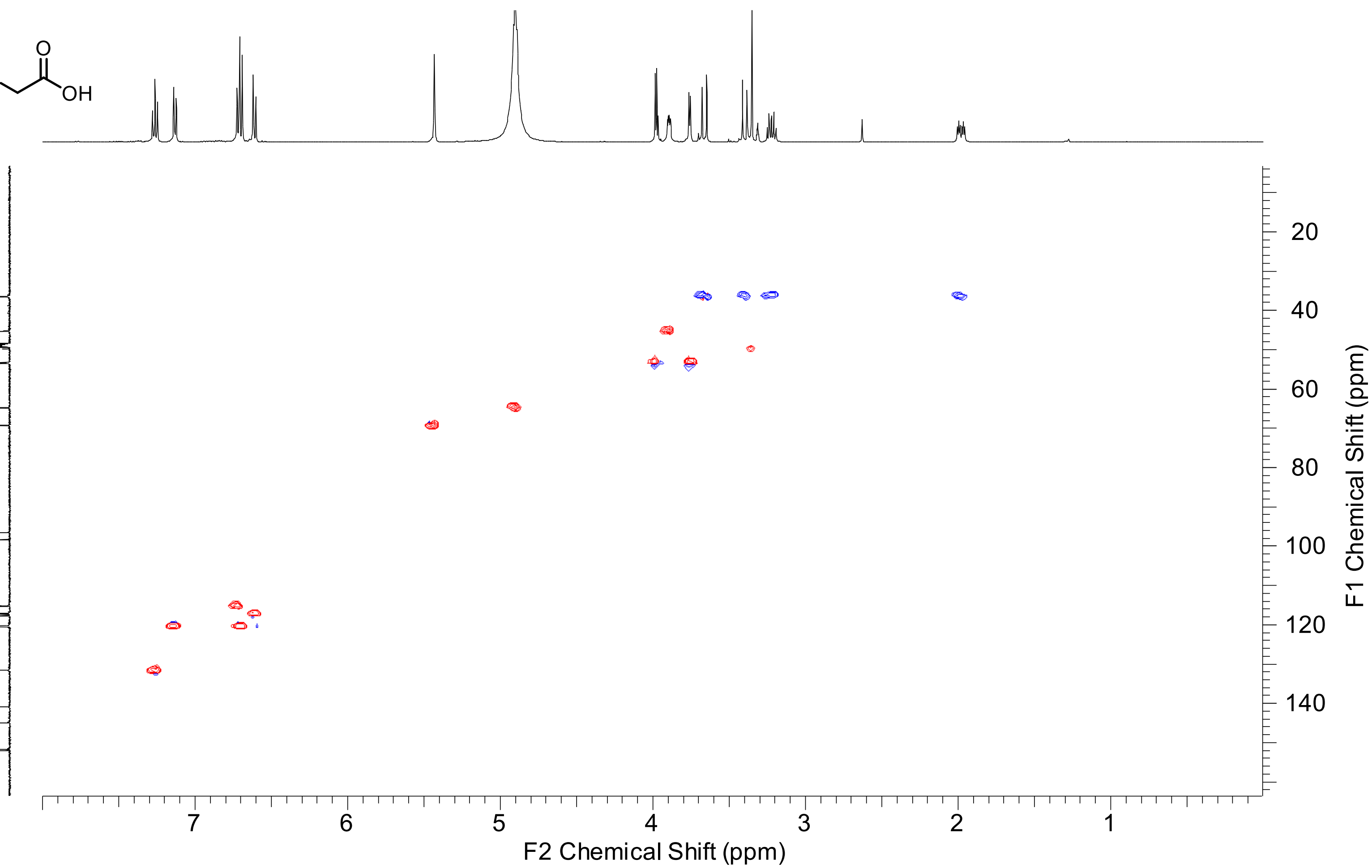

Figure S8. HSQC spectrum of sulphopreussomerin A (**1**)

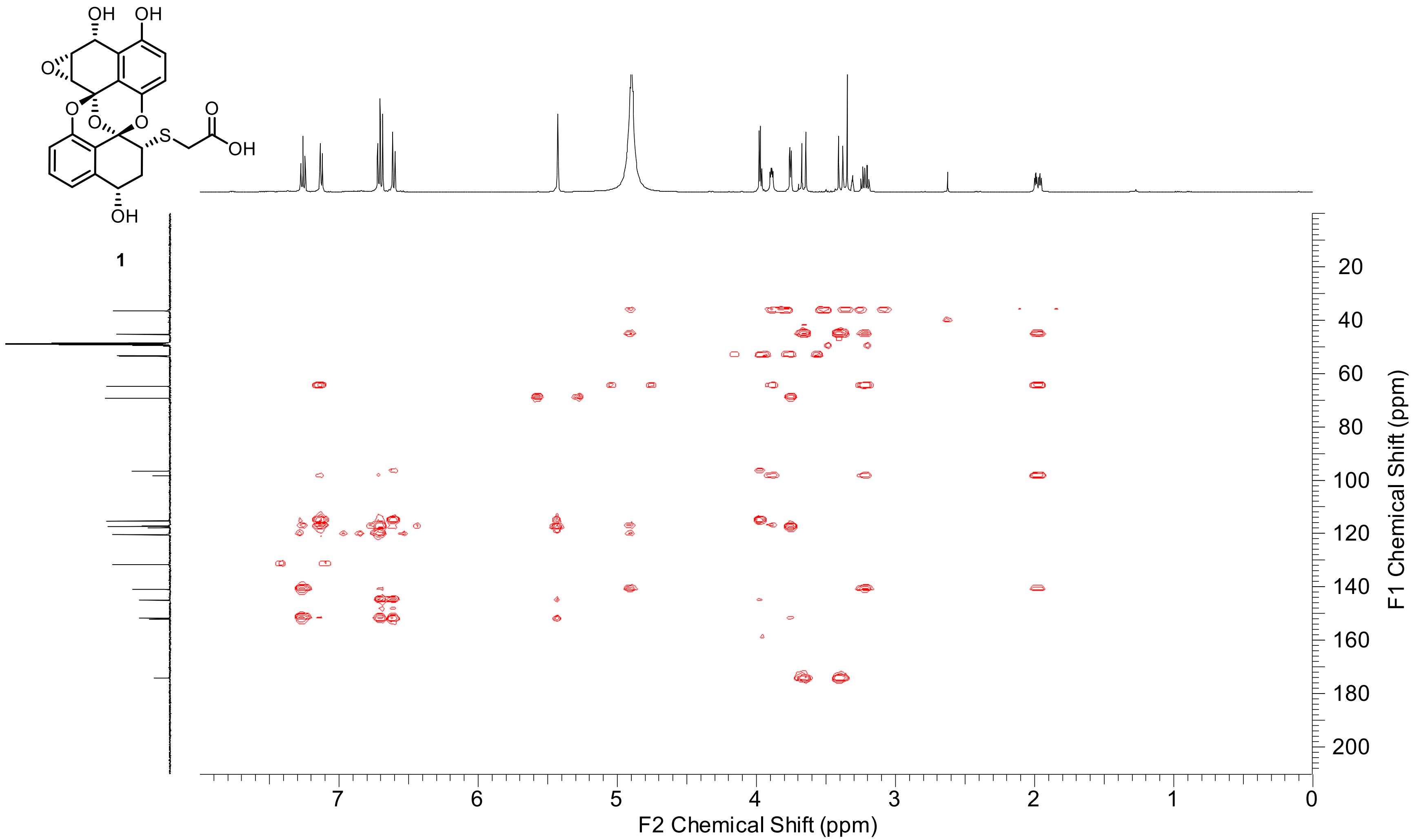

Figure S9. HMBC spectrum of sulphopreussomerin A (**1**)

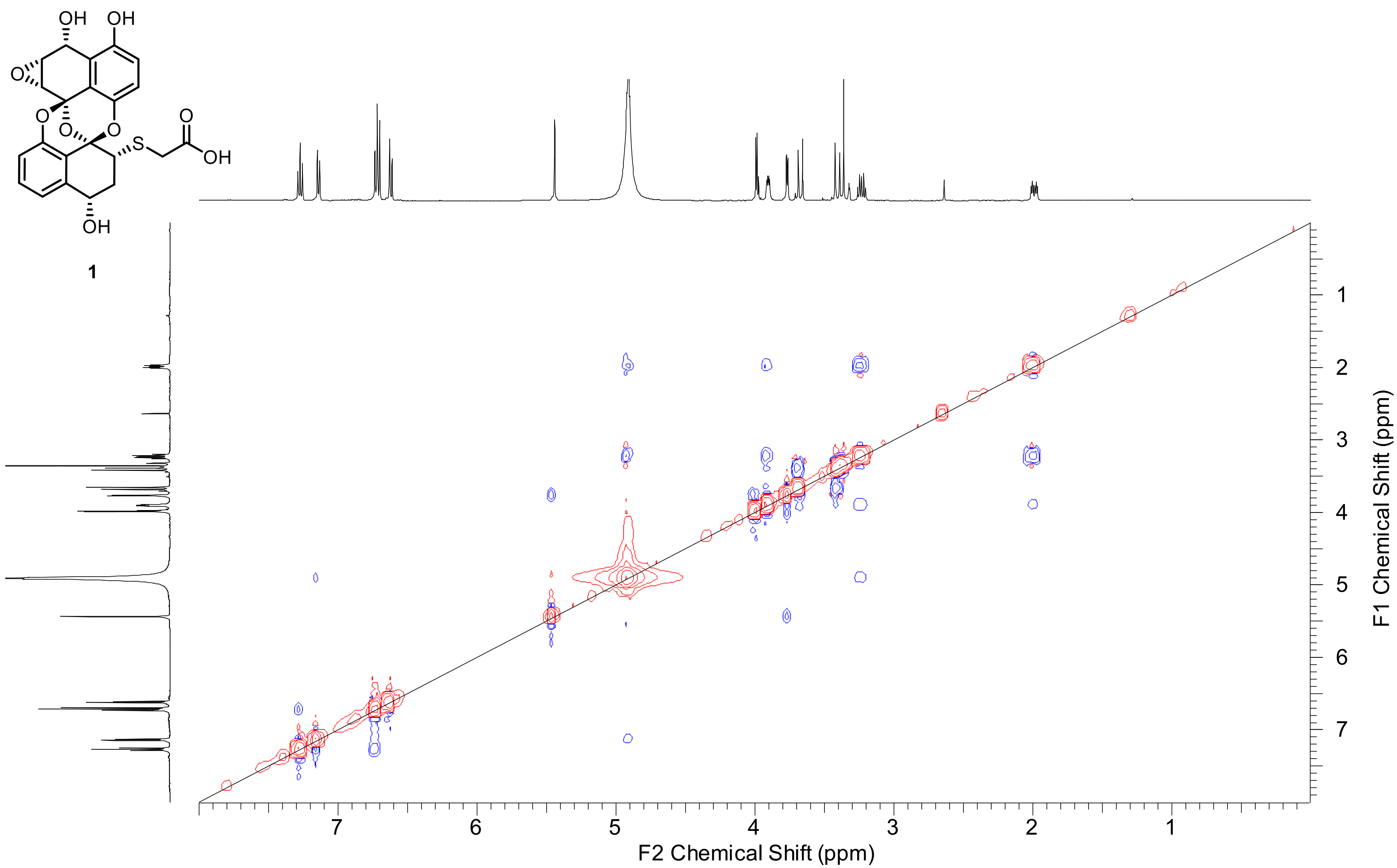

Figure S10. NOESY spectrum of sulphopreussomerin A (**1**)

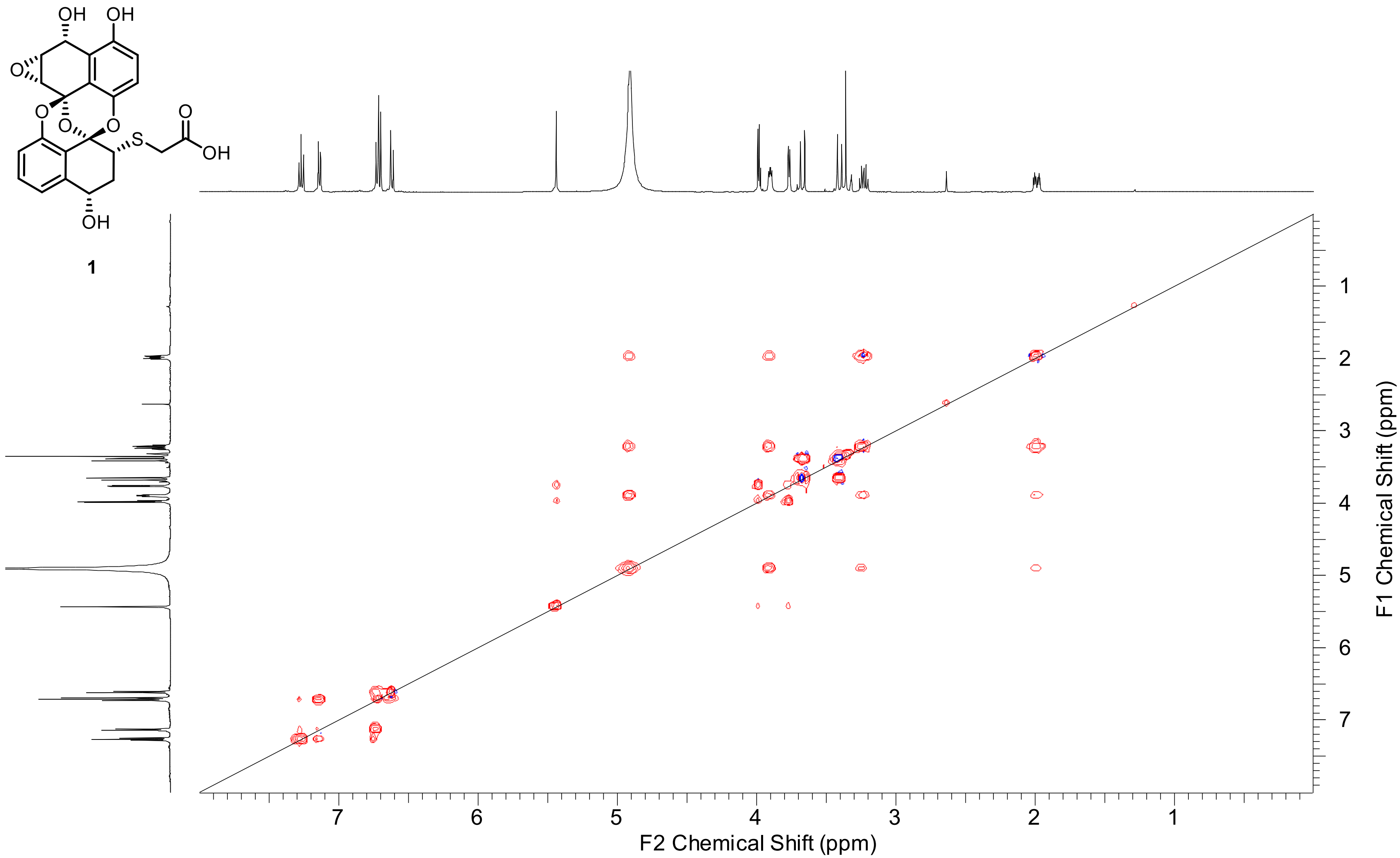

Figure S11. TOCSY spectrum of sulphopreussomerin A (**1**)

3'S 3'R

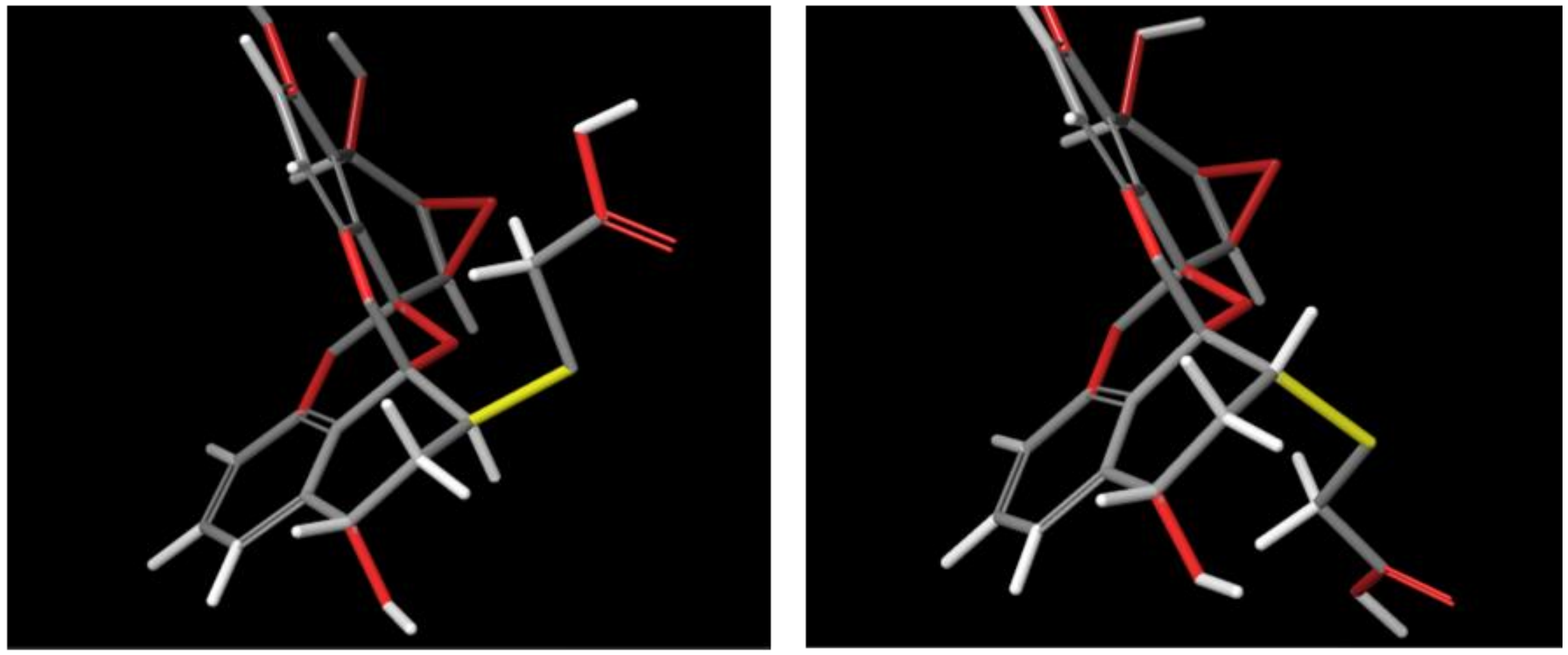

	3'R	*3'S*	*Exp. coupling constant*	*Predicted dihedral angle**
	Dihedral angle according Maestro			
H_1-H_{2a}	38°	40°	4.9 Hz	≈51°, 125°
H_1-H_{2b}	78°	78°	4.9 Hz	≈51°, 125°
H_{2a}-H_3	50°	175°	7.1 Hz	≈42°, 133°
H_{2b}-H_3	65°	66°	3.8 Hz	≈56°, 120°

* Determined using https://www.stenutz.eu/conf/haasnoot.php

Figure S12. Maestro-minimized structures of epimer-b (3′*S*, top left) and epimer-a (3′*R*, top right) sulphopreussomerin A (**1**) using MacroModel and the MMFFs force field. (Bottom) Comparison table of calculated dihedral angles versus experimental coupling constants (*J* in Hz).

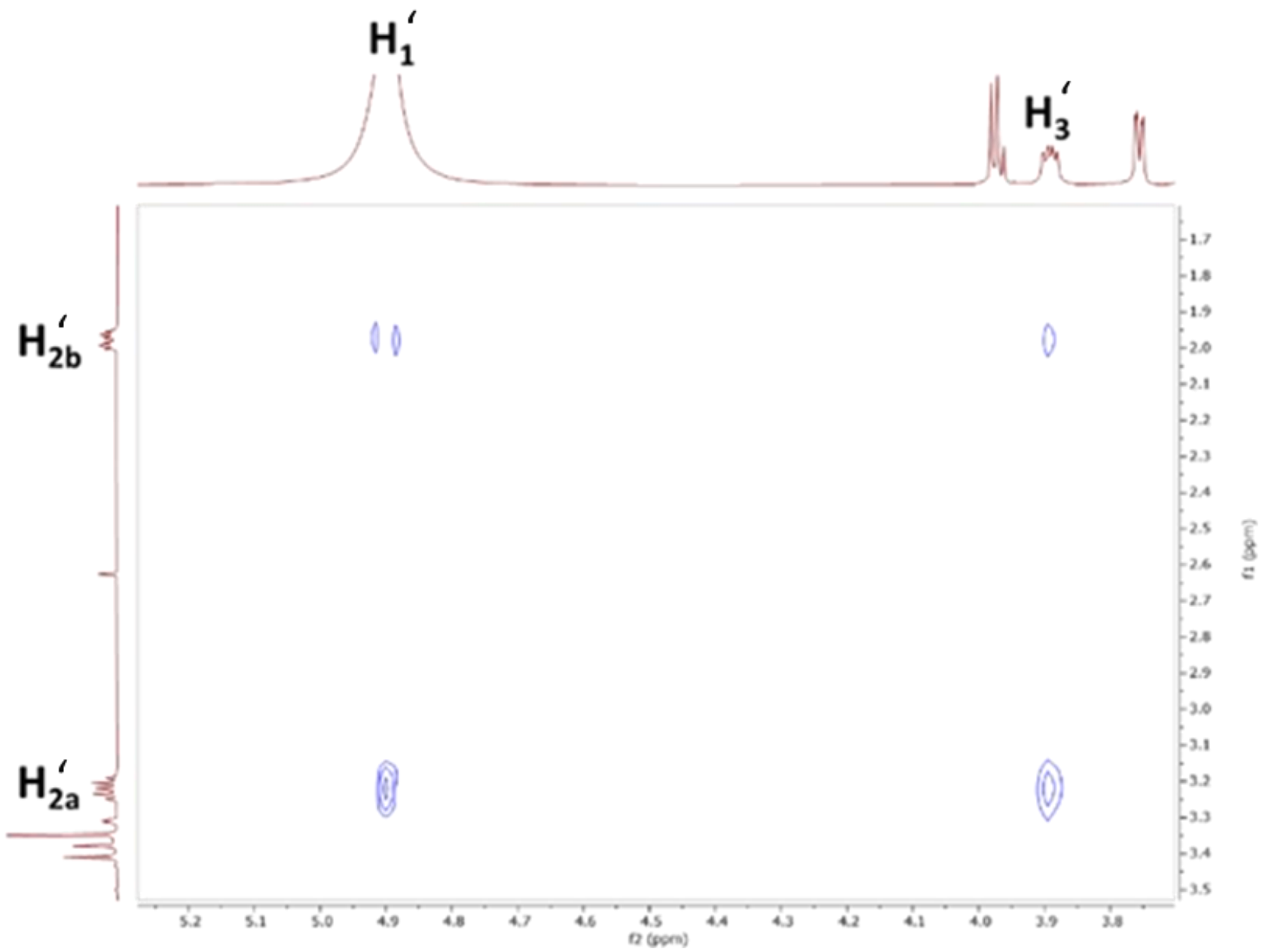

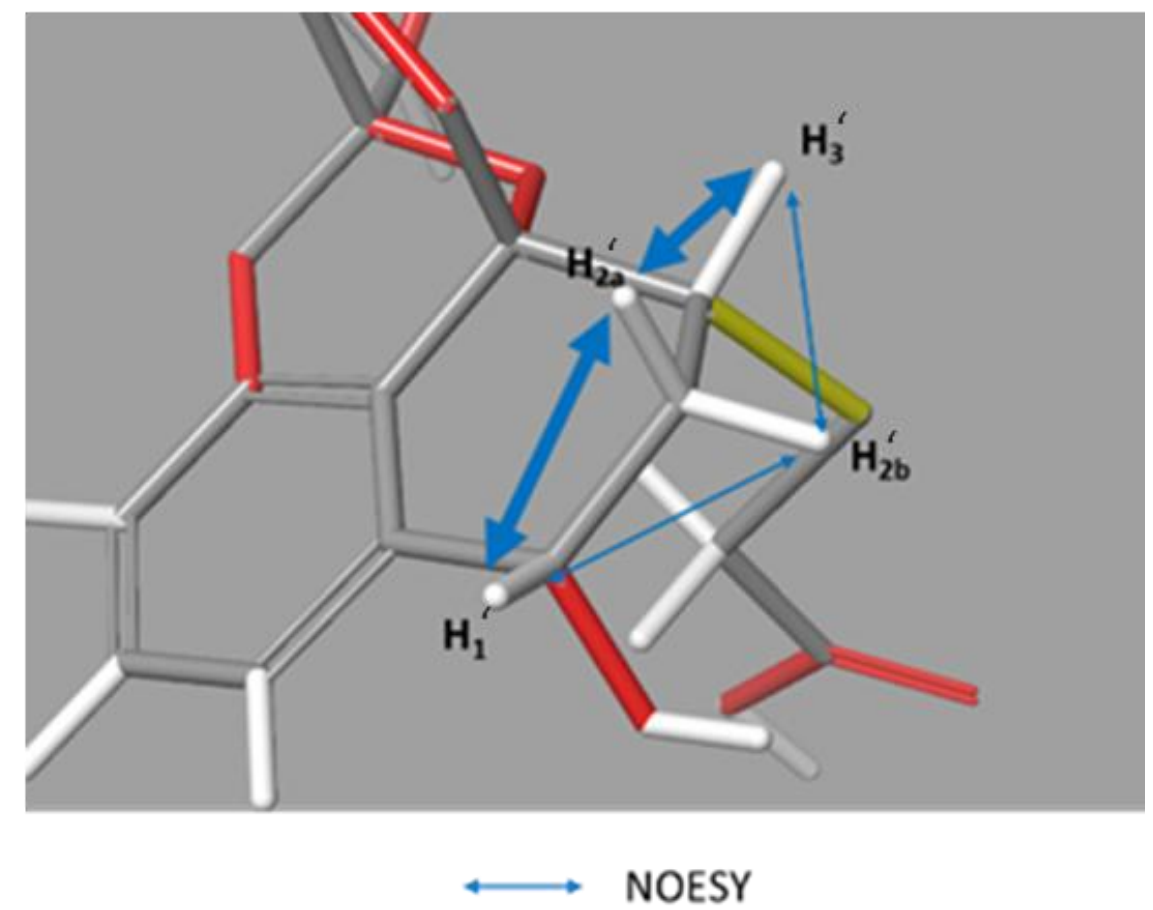

Figure S13. Expansion of the NOESY spectrum (left) of sulphopreussomerin A (**1**), showing the same pattern between H3′-H2′a-H2′b and H1′-H2′a-H2′b, with a more intense signal for H2′a (3.22 ppm) and a less intense signal for H2′b (1.98 ppm), supporting a common orientation of H1′ and H3′. This observation is consistent with the Maestro-minimized 3′*R* structure (epimer-a) of sulphopreussomerin A (**1**) (right).

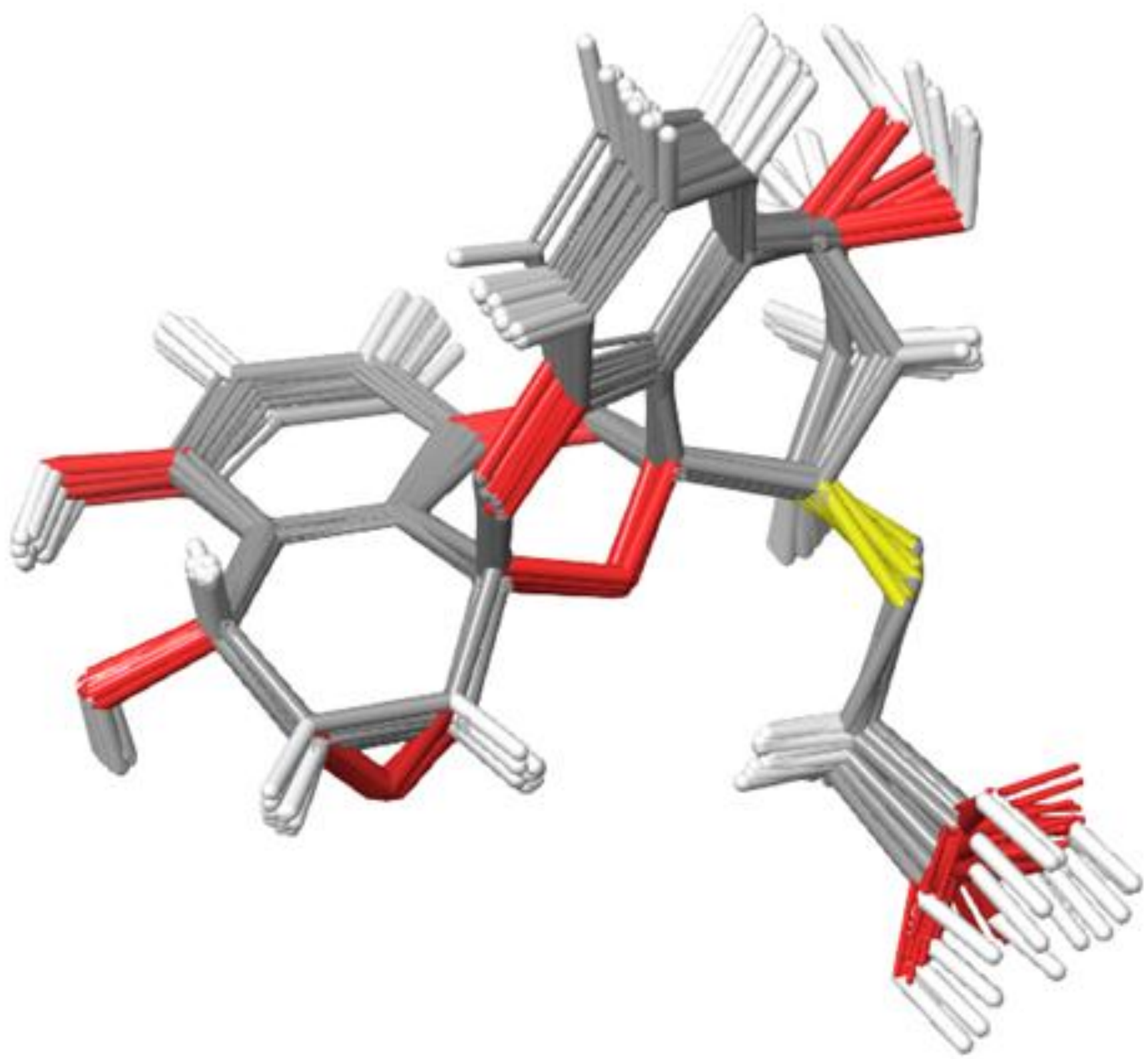

Figure S14. Overlaid 3D structures for 1*R*, 2*R*, 3*R*, 4*S*, 1′*S*, 3′*R*, 4′*S*-Sulphopreussomerin A conformers.

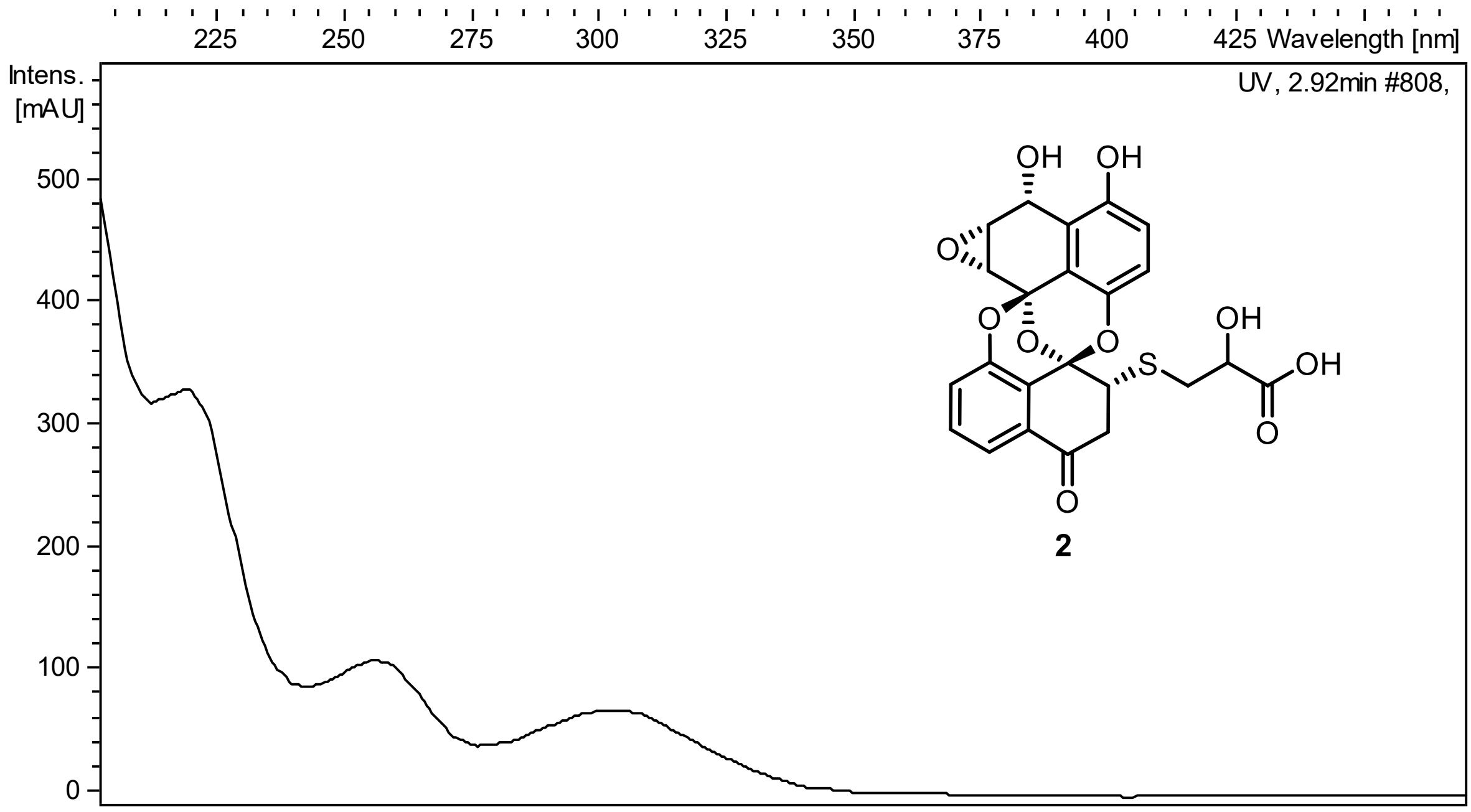

Figure S15. DAD UV-vis spectrum of sulphopreussomerin B (**2**) in methanol (MeOH)

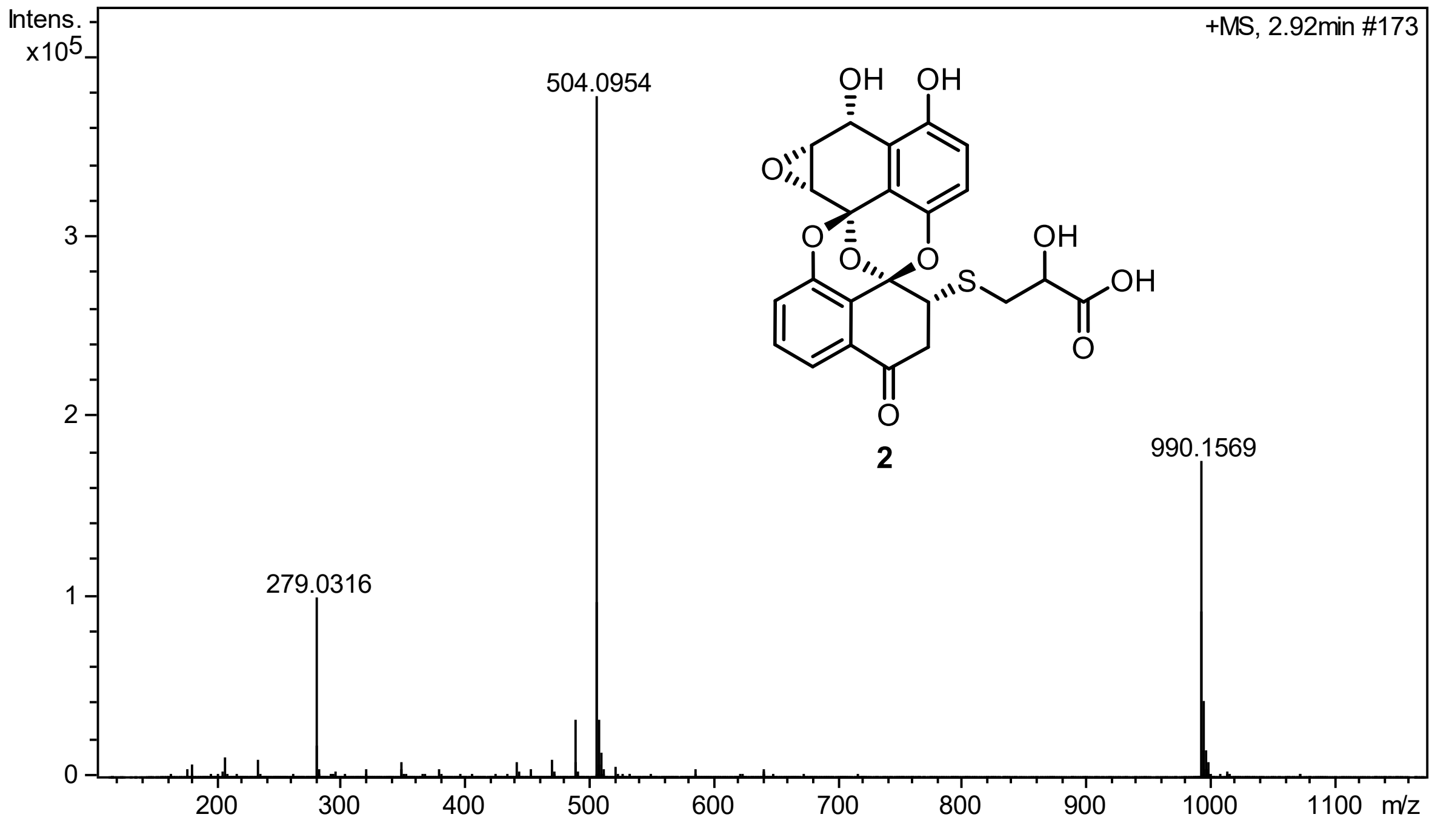

Figure S16. HR ESI-TOF MS spectrum of sulphopreussomerin B (**2**)

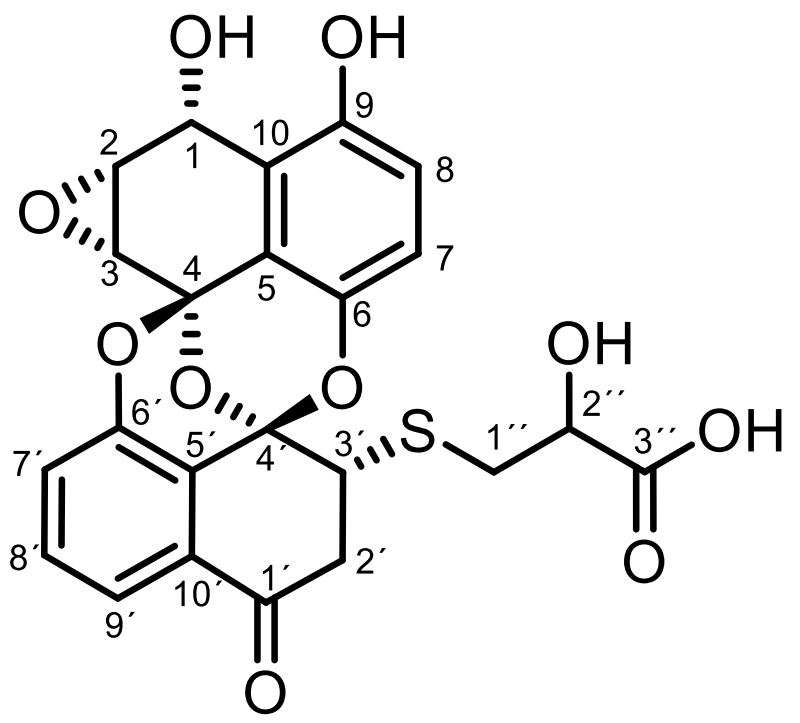

Table S3. NMR spectroscopic data of sulphopreussomerin B (**2**)

**Pos.**	***δ*C, type**	***δ*H, mult. (*J* in Hz)**	**COSY**	**HMBC (H to C)**	**NOESY**
1	69.1, CH	5.51, s	2	3, 4, 5, 6, 9, 10	2
2	53.7, CH	3.87, dd (4.6, 1.1)	3, 1	1, 9, 10	1
3	53.3, CH	4.09, d (4.6)	2	1, 4, 5, 6	
4	96.9, C				
5	115.0, C				
6	144.3, C				
7	117.7, CH	6.77, d (8.9)		4, 5, 6, 9	
8	121.0, CH	6.81, d (8.9)		1, 6, 5, 10	
9	152.5, C				
10	118.0, C				
1′	195.0, C				
2′	42.9, CH_2	a: 3.07, dd (18.5, 2.0)	2′, 3′	1′, 3′, 4′, 10′	3′
		b: 3.77, dd (18.5, 5.0)	2′, 3′		
3′	48.9, CH	4.33, dd (5.0, 2.0)	2′	1′, 2′, 4′, 5′, 1″, 6′	2′, 1″
4′	97.1, C				
5′	122.1, C				
6′	152.1, C				
7′	122.9, CH	7.19, dd (7.8, 0.9)	8′	6′, 9′, 4′	8′
8′	132.4, CH	7.48, t (7.8)	7′, 9′	1′, 6′, 10′	9′
9′	121.1, CH	7.57, dd (7.8, 0.9)	8′	1′, 5′, 7′, 4′, 6′	
10′	131.4, C				
1″	38.1, CH_2	a: 3.06, dd (13.8, 6.2)	1″, 2″	3′, 2″, 3″	3′
		b: 3.31, dd (13.8, 4.2)	1″, 2″		
2″	72.1, CH	4.41, dd (6.2, 4.2)	1″	1″, 3″	1″
3″	175.7, C				

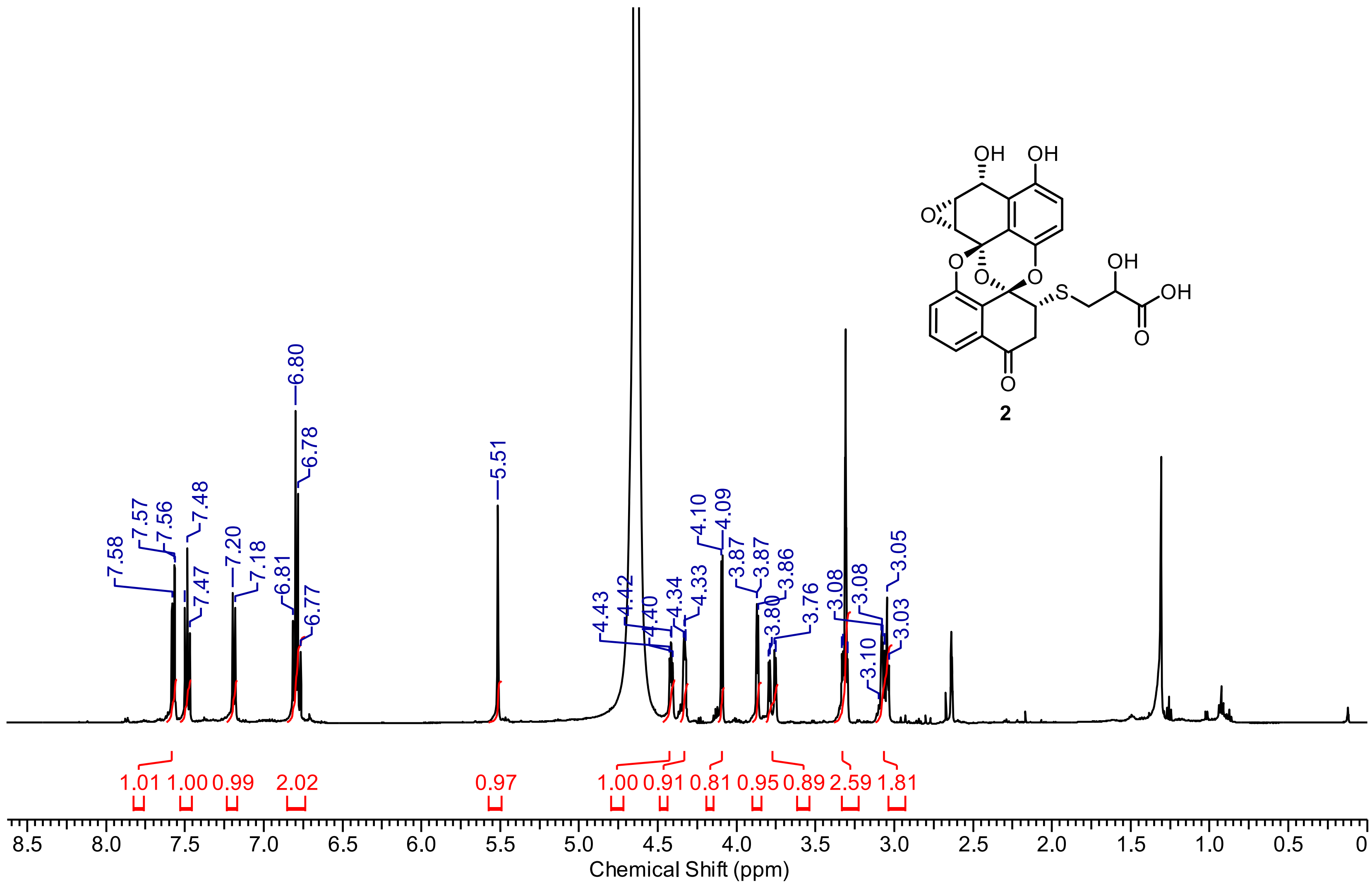

Figure S17a. ^{1}H NMR spectrum of sulphopreussomerin B (**2**) (CD_3OD/DMSO-d_6, 4:1, 500 MHz)

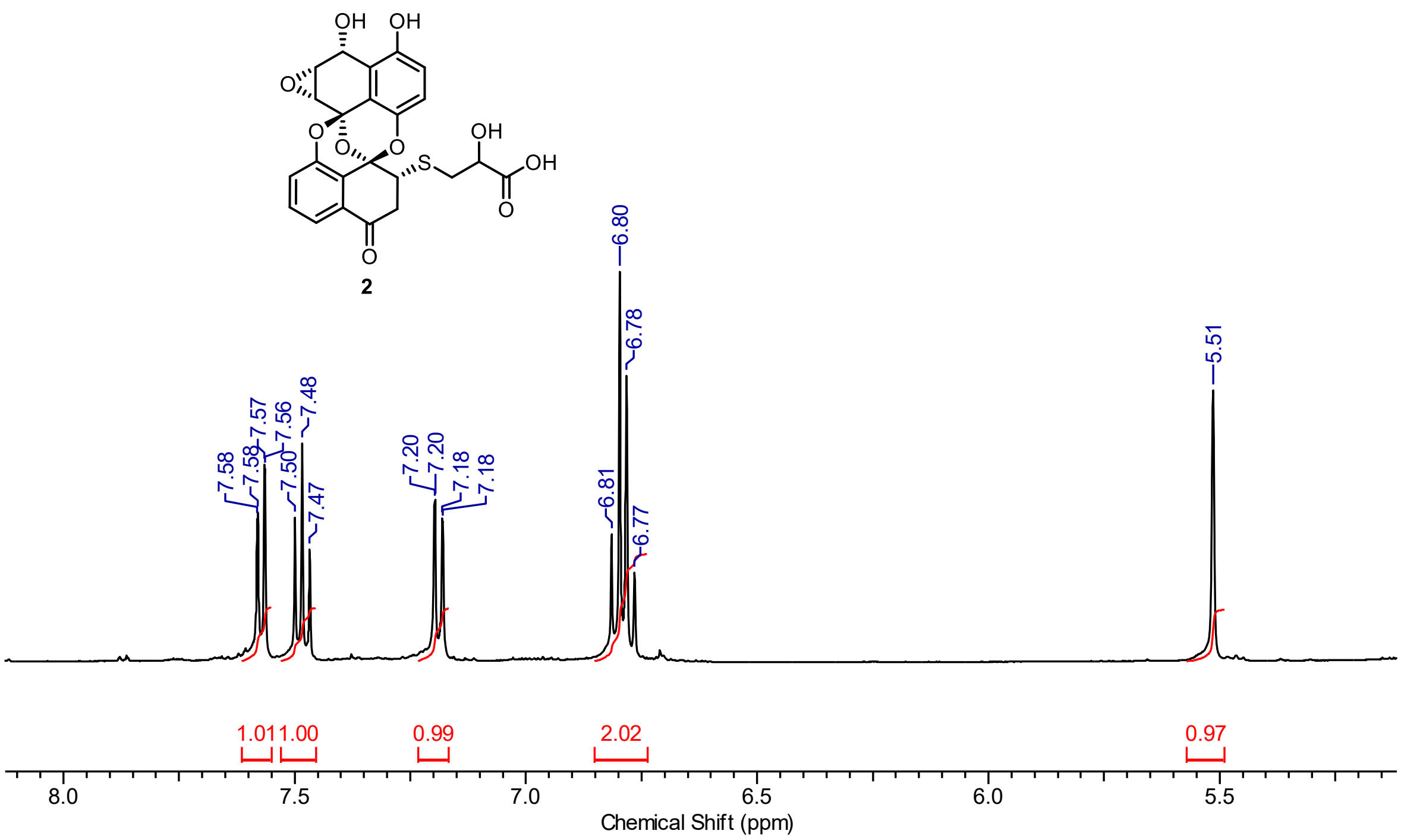

Figure S17b. Expansion of ^{1}H NMR spectrum of sulphopreussomerin B (**2**) (CD_3OD/DMSO-d_6, 4:1, 500 MHz)

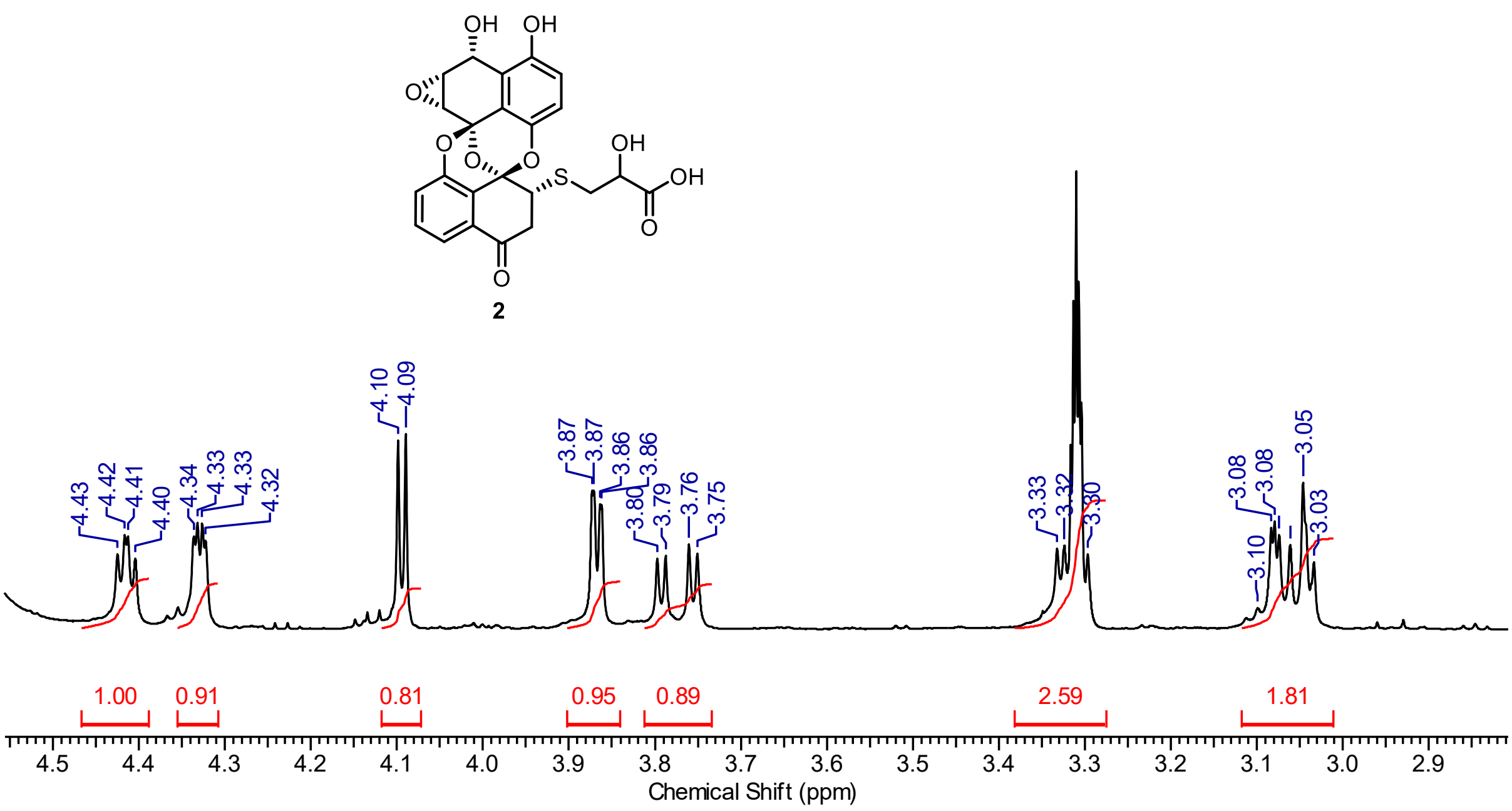

Figure S17c. Expansion of ^{1}H NMR spectrum of sulphopreussomerin B (**2**) (CD_3OD/DMSO-d_6, 4:1, 500 MHz)

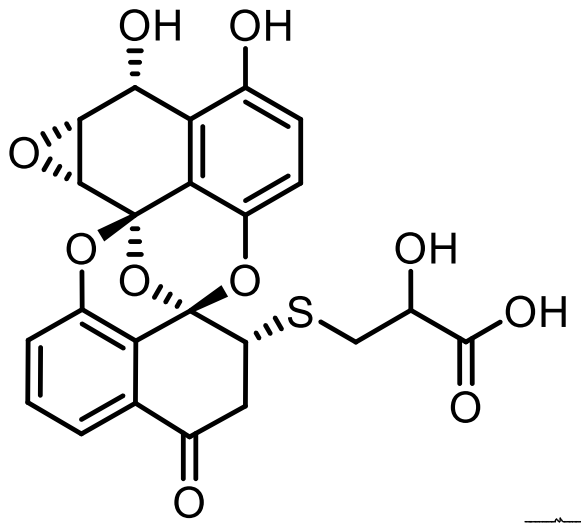

2

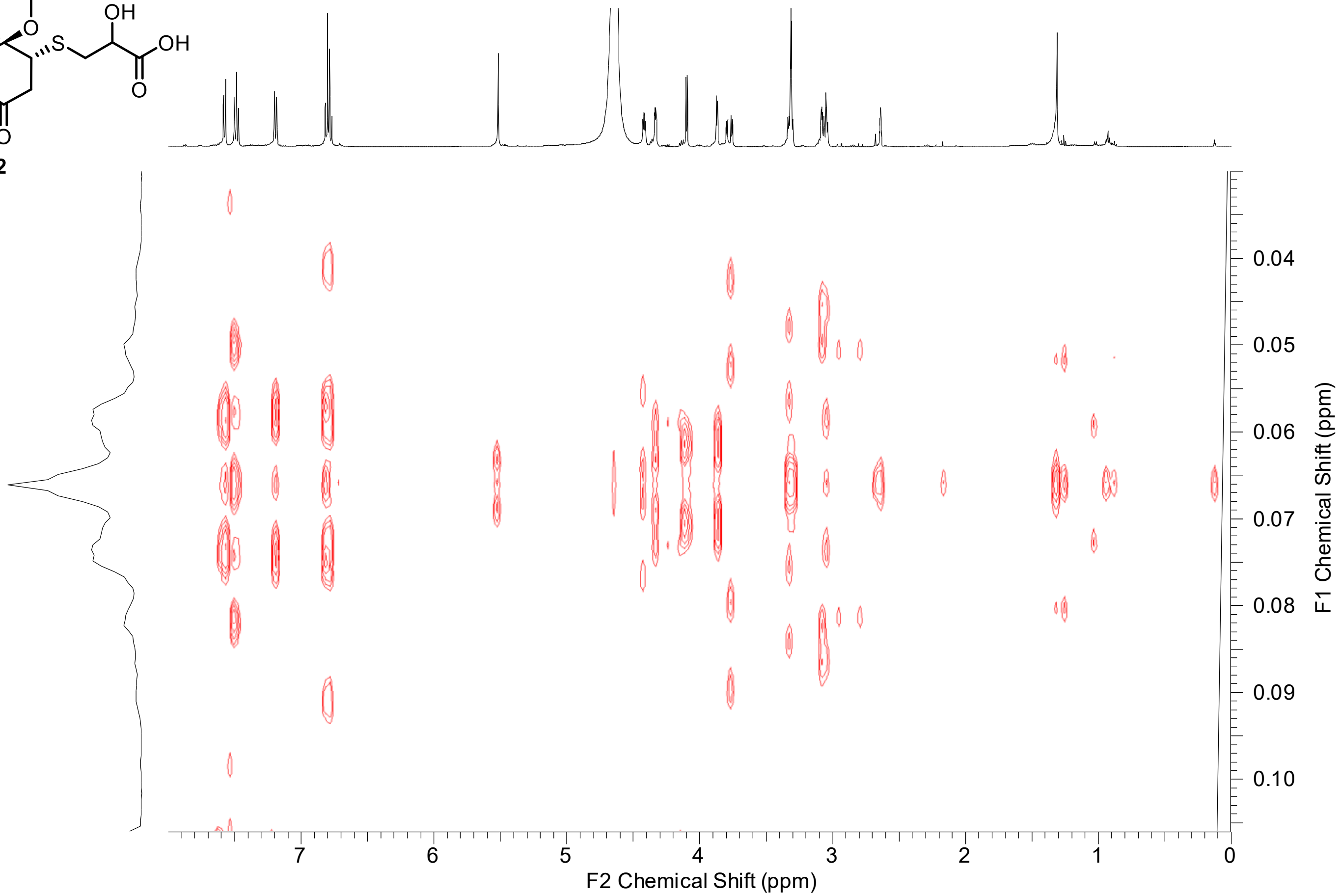

Figure S18. *J*-Resolved spectrum of sulphopreussomerin B (**2**) (CD_3OD/DMSO-d_6, 4:1, 500 MHz)

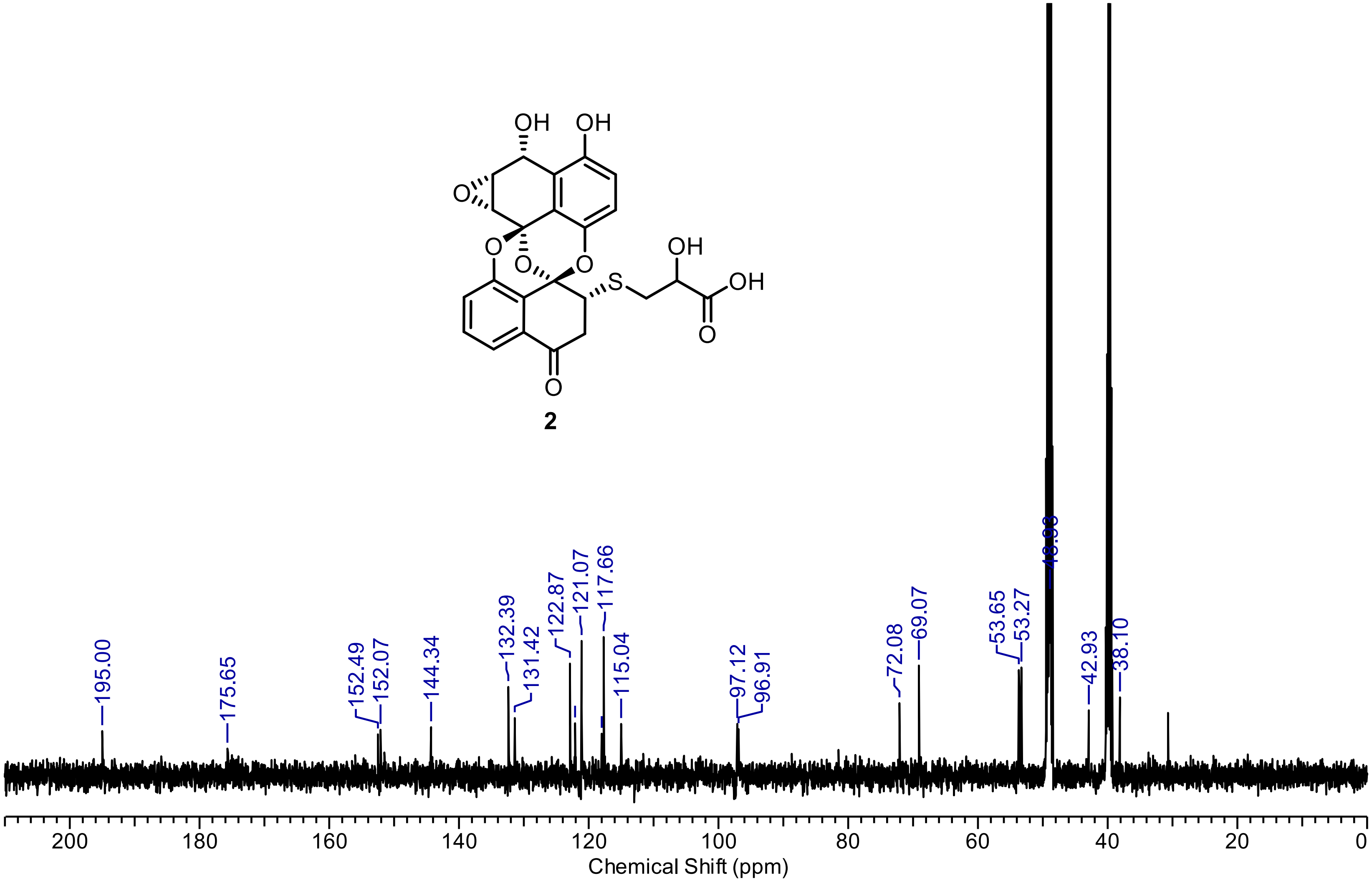

Figure S19. ^{13}C NMR spectrum of sulphopreussomerin B (**2**) (CD_3OD/DMSO-d_6, 4:1, 125 MHz)

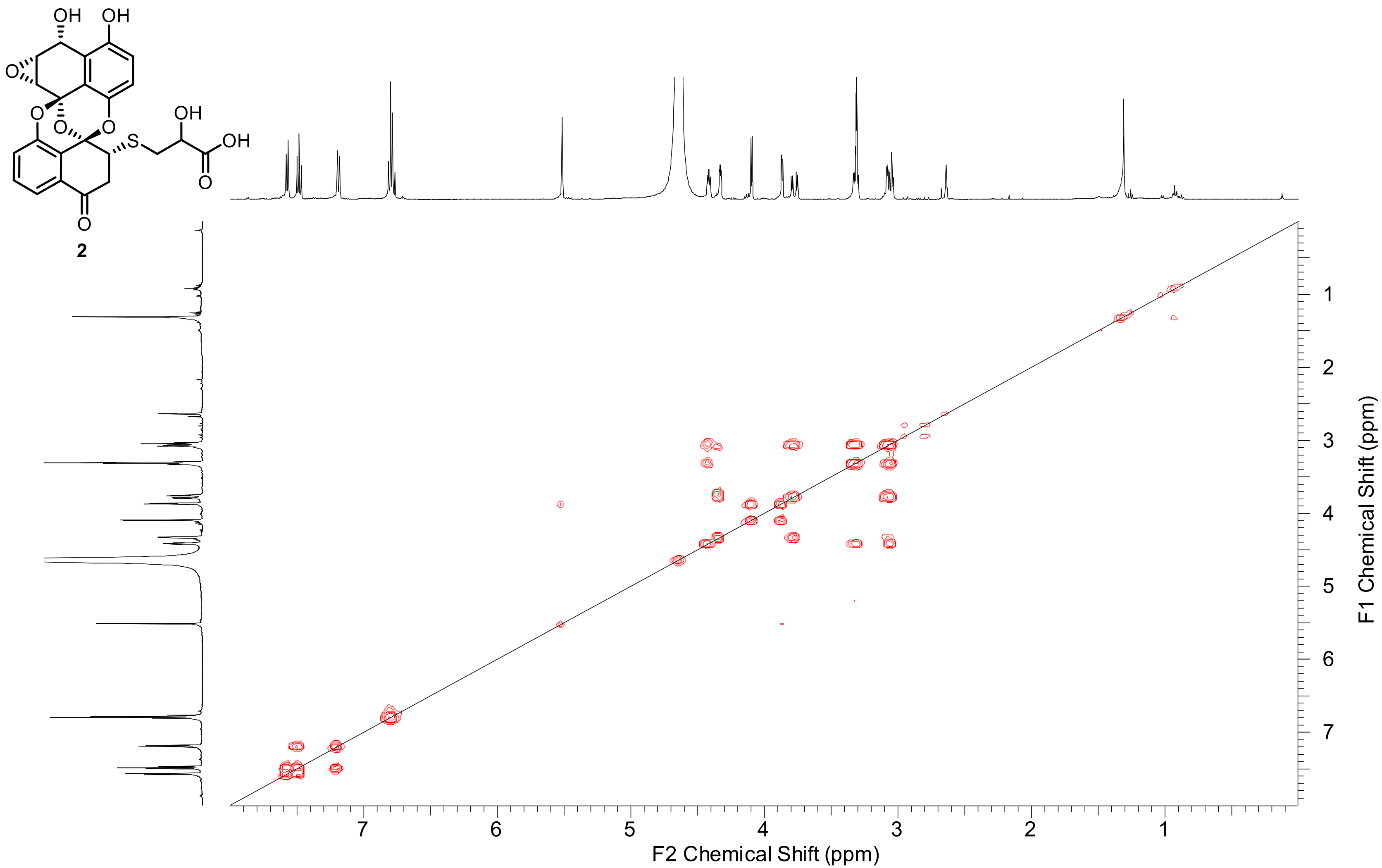

Figure S20. COSY spectrum of sulphopreussomerin B (**2**)

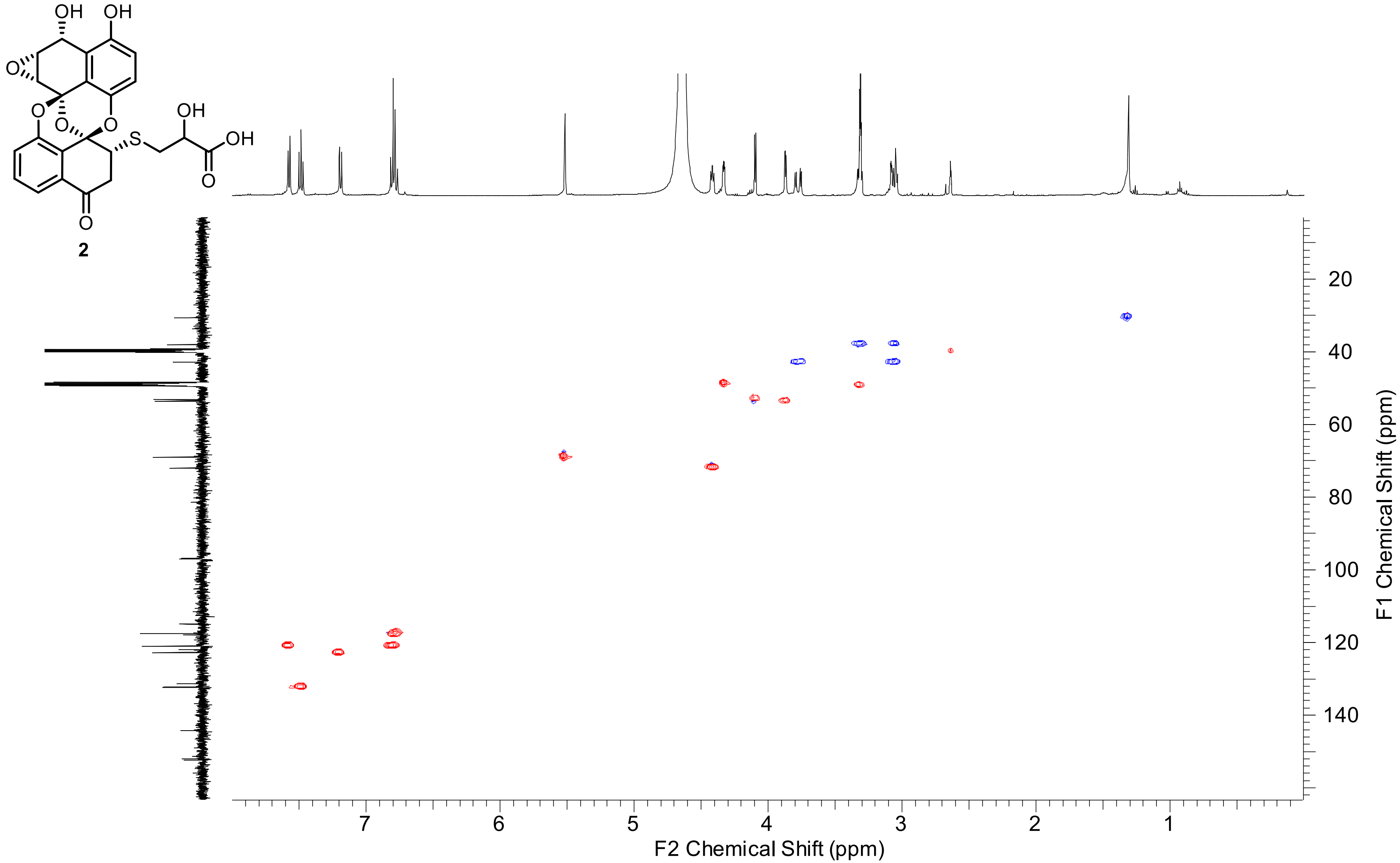

Figure S21. HSQC spectrum of sulphopreussomerin B (**2**)

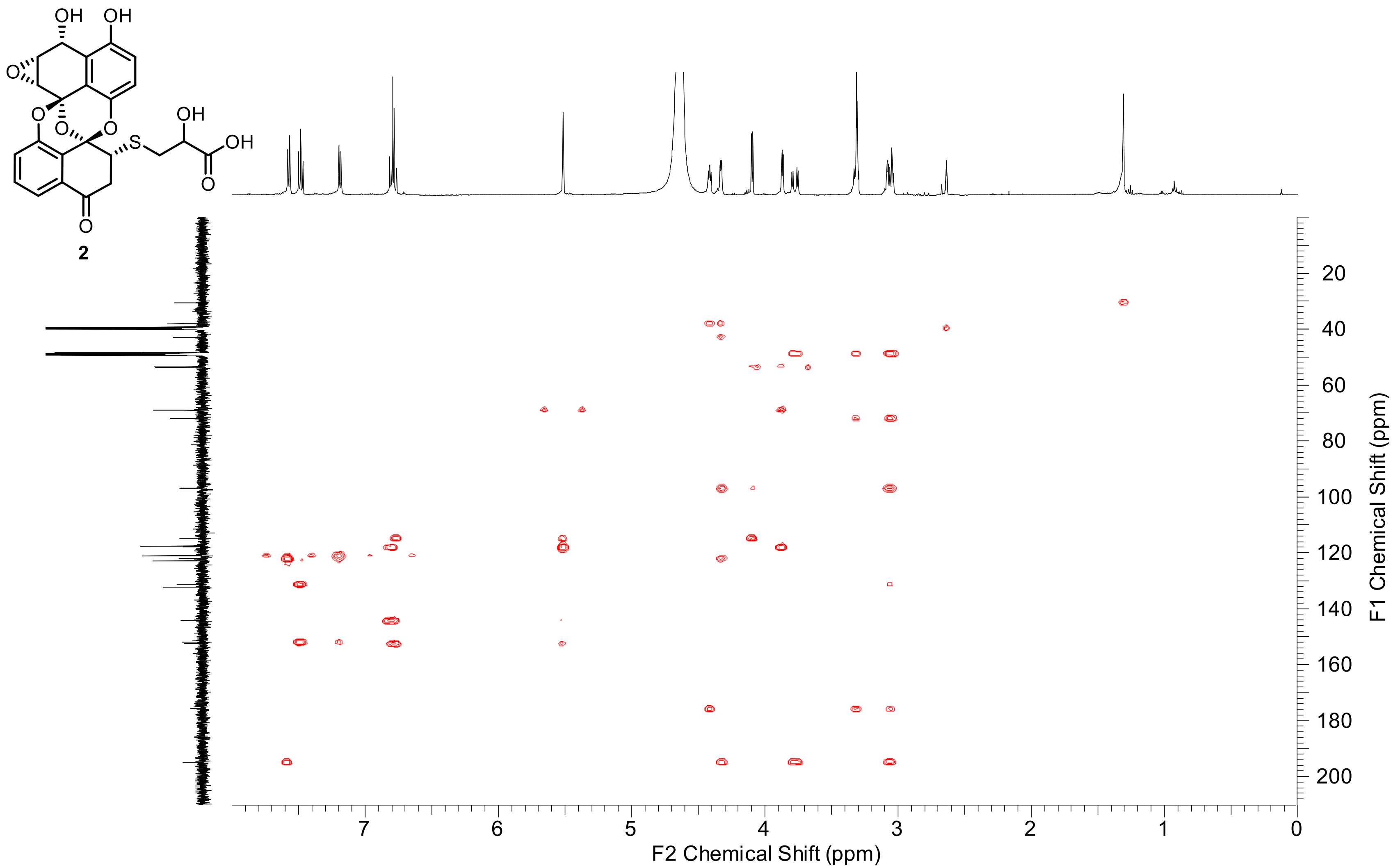

Figure S22. HMBC spectrum of sulphopreussomerin B (**2**)

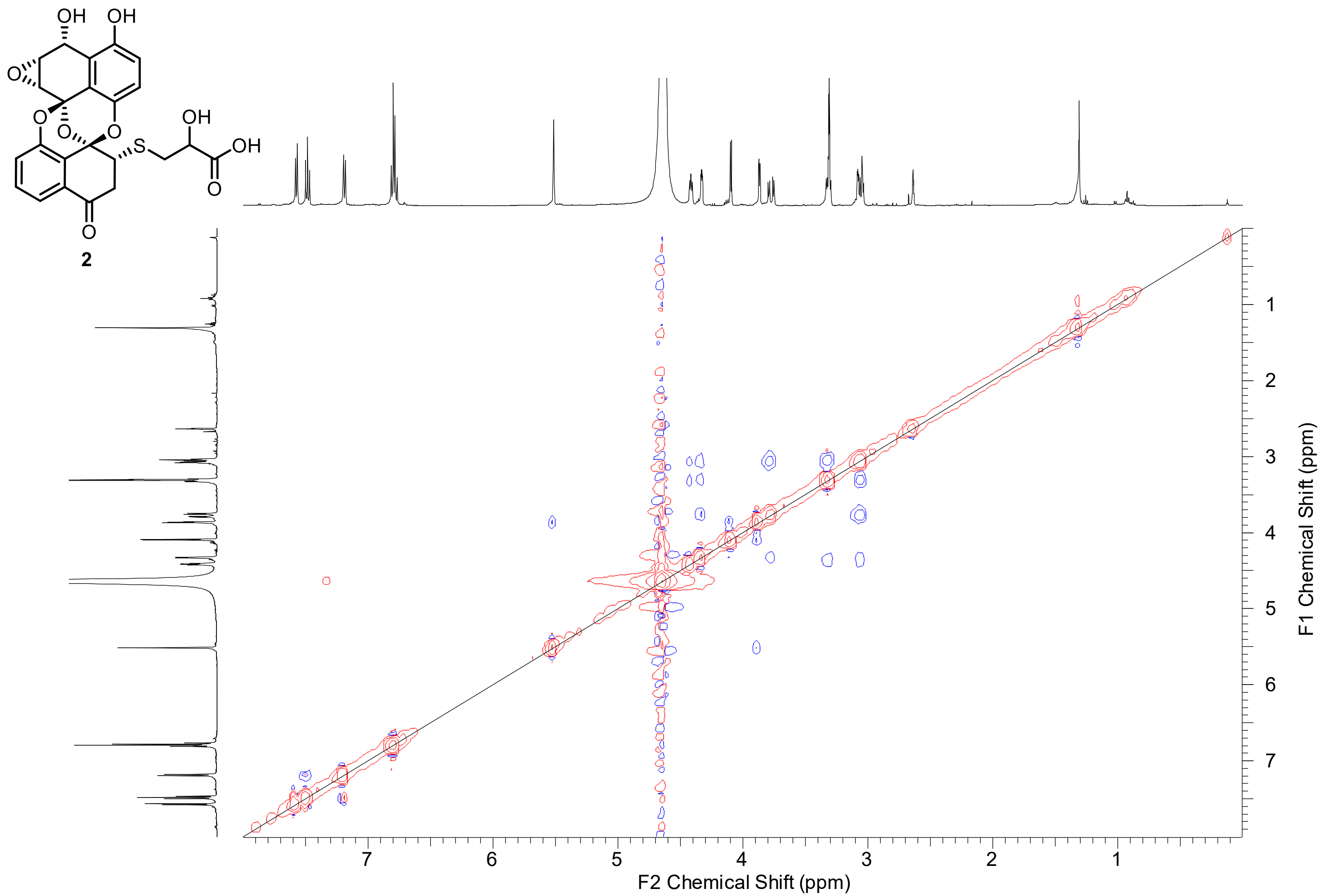

2

Figure S23. NOESY spectrum sulphopreussomerin B (**2**)

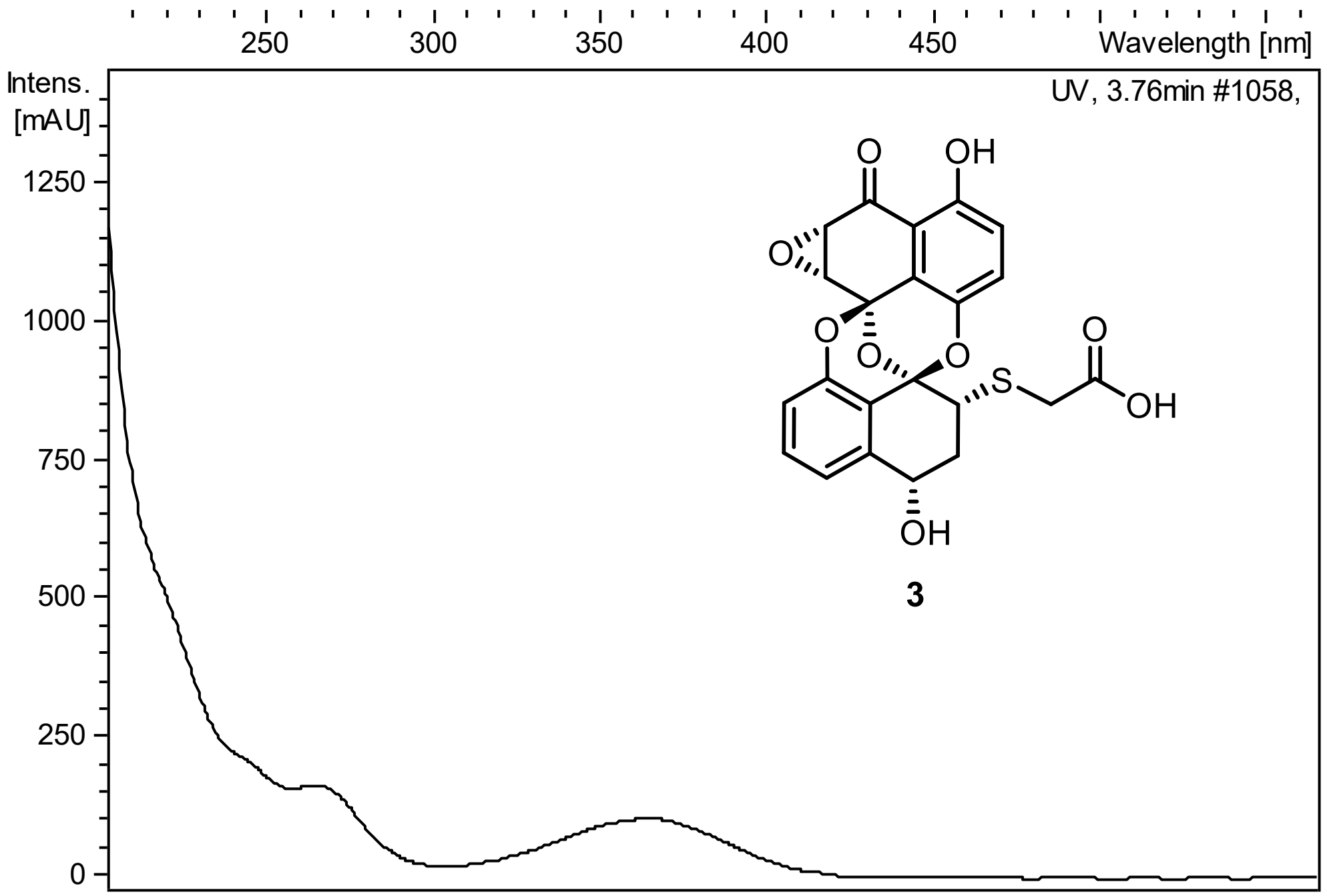

Figure S24. DAD UV-vis spectrum of sulphopreussomerin C (**3**) in methanol (MeOH)

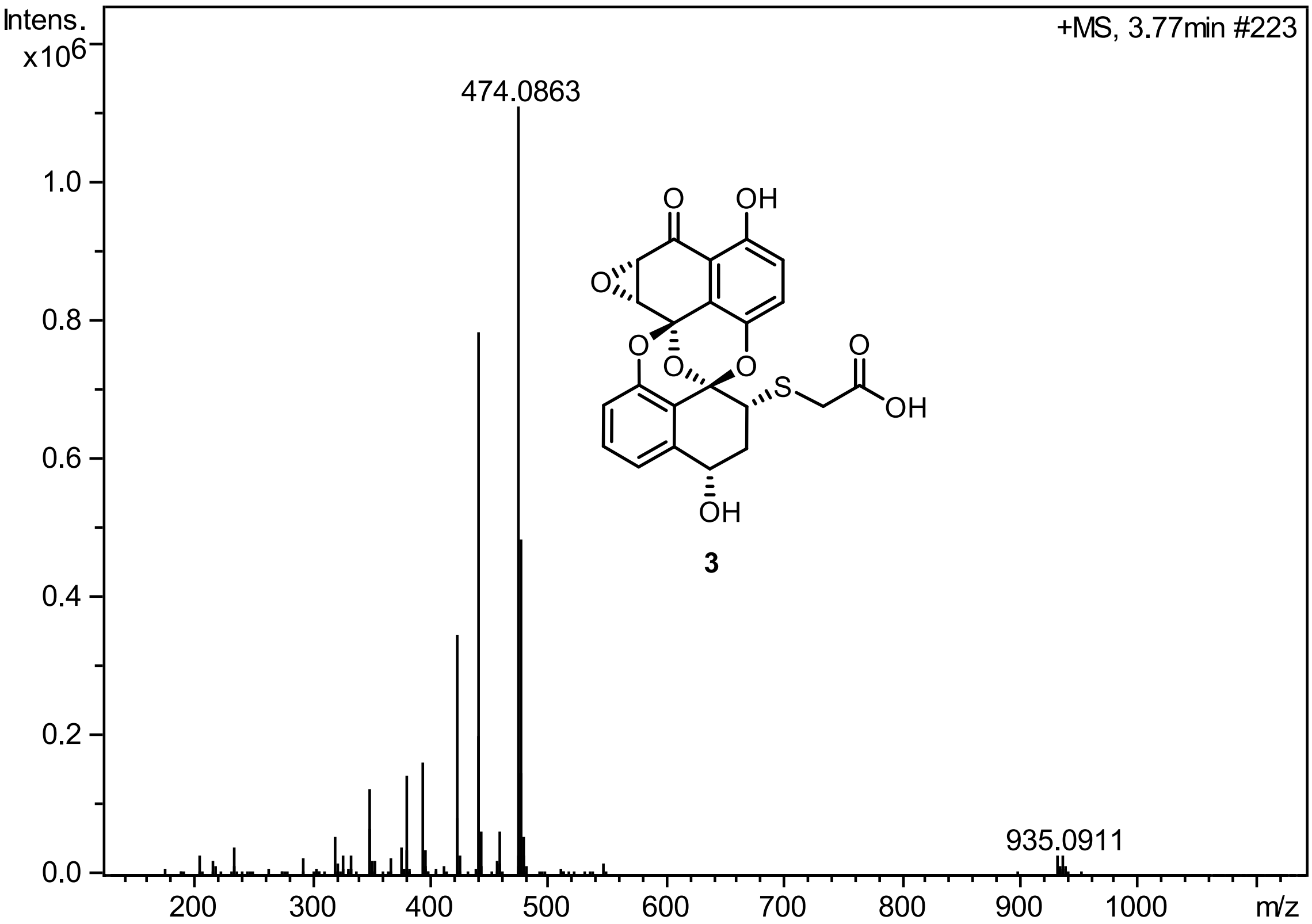

Figure S25a. HR ESI-TOF MS spectrum of sulphopreussomerin C (**3**)

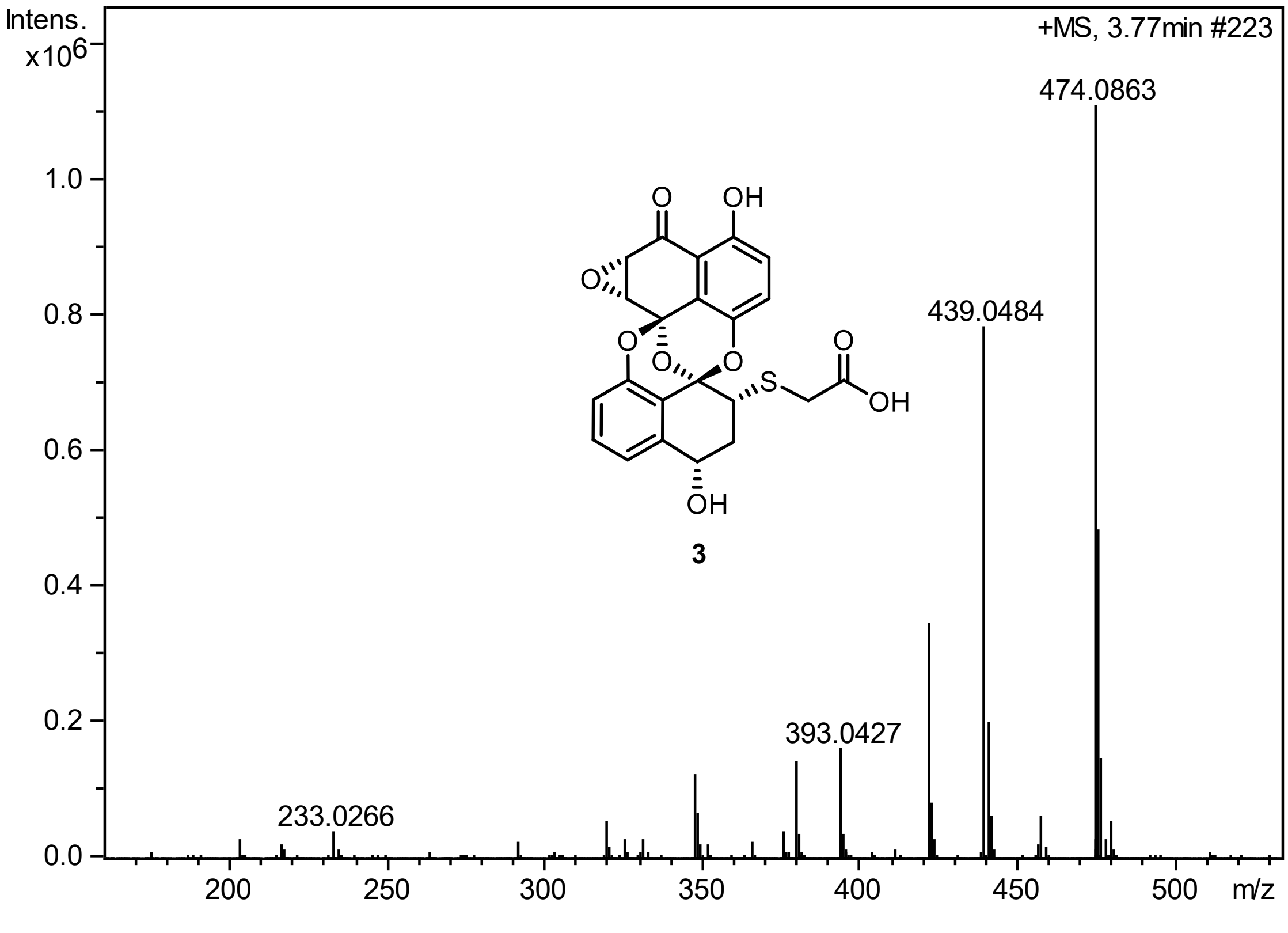

Figure S25b. Expansion of HR ESI-TOF MS spectrum of sulphopreussomerin C (**3**)

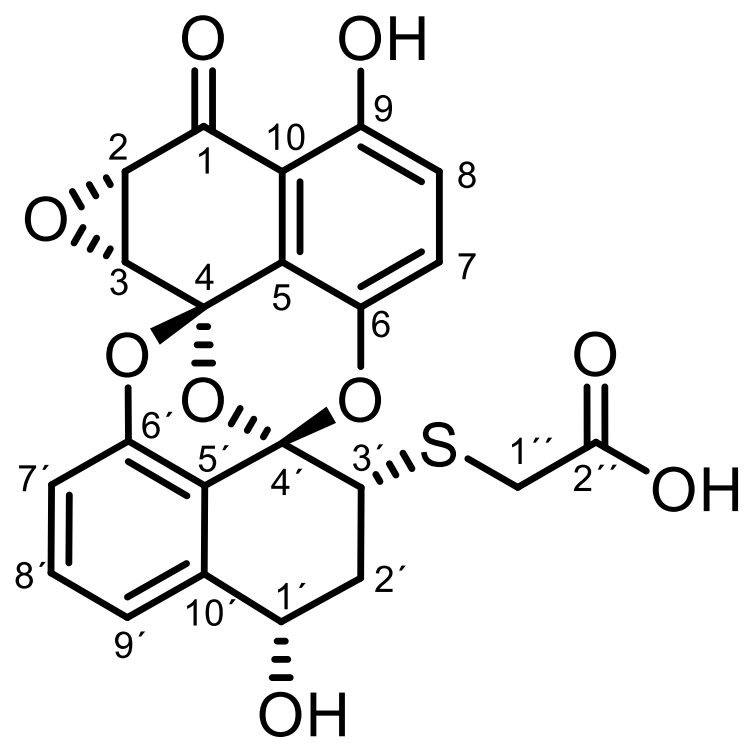

Table S4. NMR spectroscopic data of sulphopreussomerin C (**3**)

Pos.	δ_C, type	δ_H, mult. (J in Hz)	COSY	HMBC (H to C)	NOESY
1	196.0, C				
2	54.4, CH	4.57, d (4.2)	3	3, 4, 10	3
3	53.3, CH	4.04, d (4.2)	2	1, 5	2
4	95.2, C				
5	112.6, C				
6	145.1, C				
7	122.3, CH	7.05, d (9.2)	8	5, 6, 9	
8	126.5, CH	7.18, d (9.2)	7	6, 9, 10	
9	155.2, C				
10	118.0, C				
1′	64.2, CH	4.94, dd (7.1, 3.7)	2′	2′, 3′, 5′, 10′	2′, 9′
2′	36.5, CH_2	a: 3.26, dt (14.8, 7.1)<br>b: 2.03, dt (14.8, 3.7)	1′, 3′	1′, 3′, 4′, 10′	1′, 3′
3′	44.9, CH	4.05, dd (7.1, 3.7)	2′	1′, 4′, 1″	2′, 1″
4′	99.6, C				
5′	116.8, C				
6′	150.8, C				
7′	115.3, CH	6.87, d (7.9)	8′	5′, 6′, 9′	8′
8′	132.2, CH	7.41, t (7.9)	7′, 9′	6′, 10′	7′, 9′
9′	121.4, CH	7.26, d (7.9)	8′	1′, 5′, 7′	1′, 8′
10′	141.9, C				
1″	36.9, CH_2	a: 3.71, d (15.3)<br>b: 3.51, d (15.3)		3′, 2″	3′
2″	173.2, C				

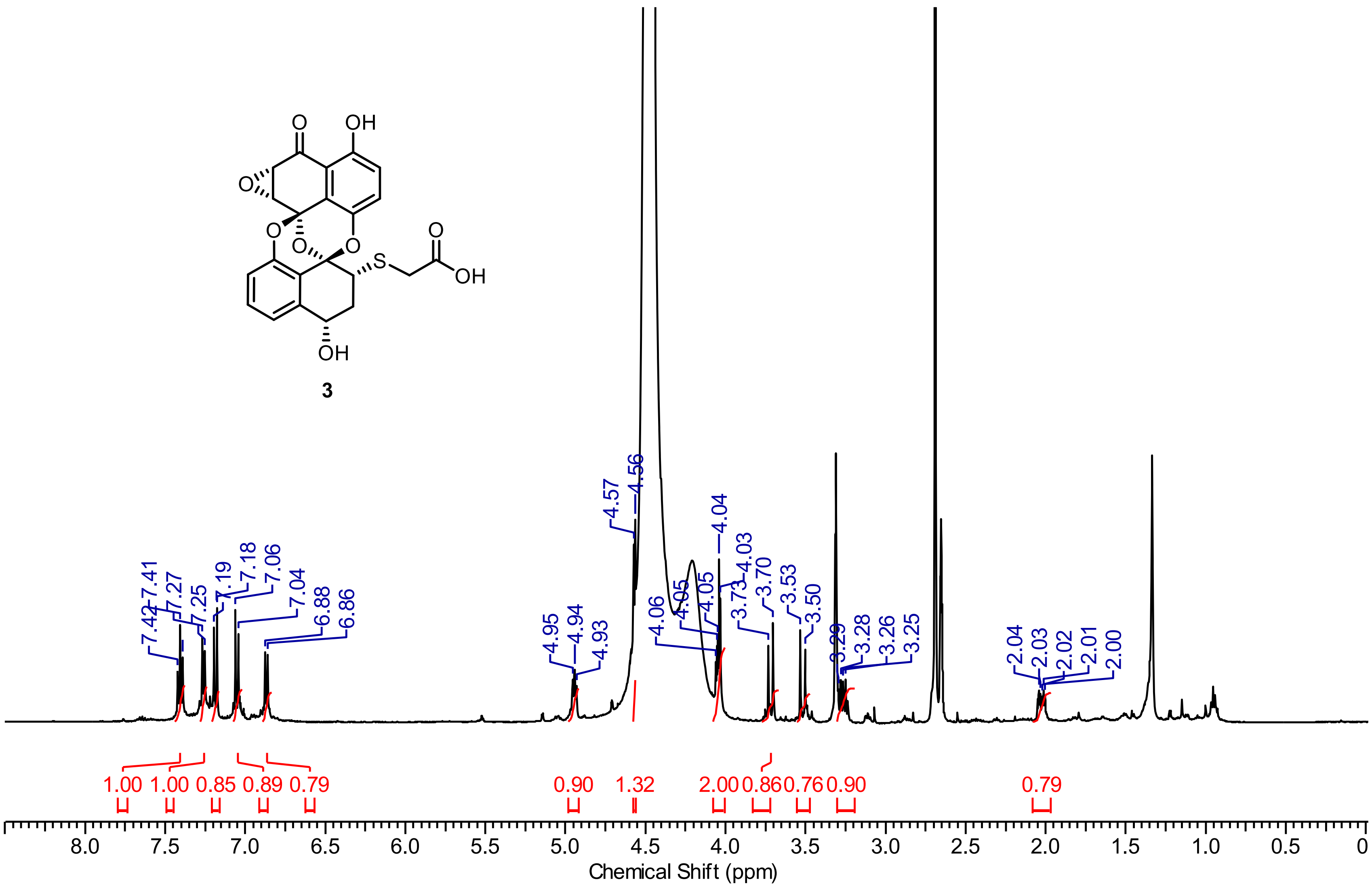

Figure S26a. ^{1}H NMR spectrum of sulphopreussomerin C (**3**) **(**CD_3OD/DMSO-d_6, 3:2, 500 MHz)

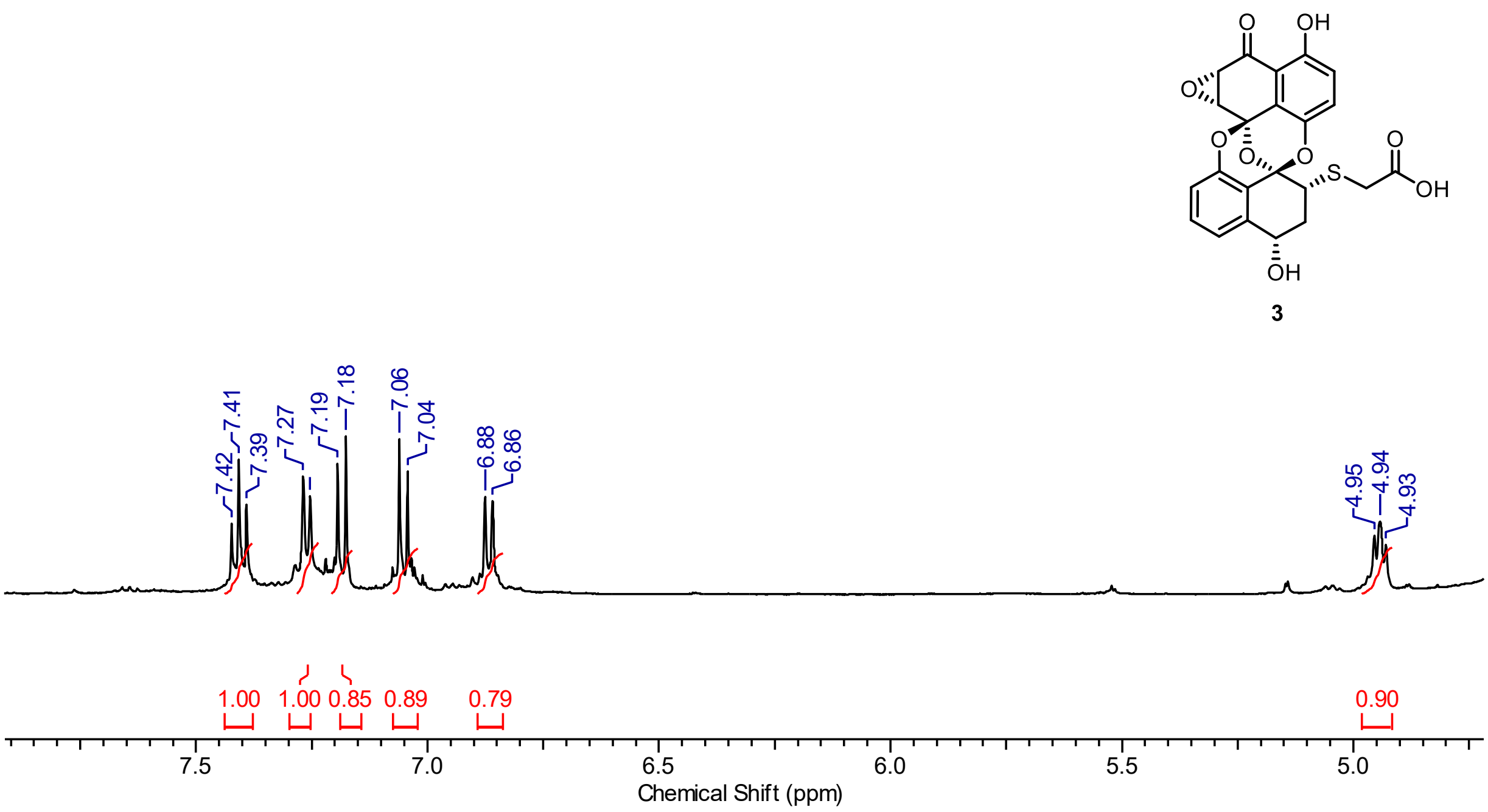

Figure S26b. Expansion of ^{1}H NMR spectrum of sulphopreussomerin C (**3**) **(**CD_3OD/DMSO-d_6, 3:2, 500 MHz)

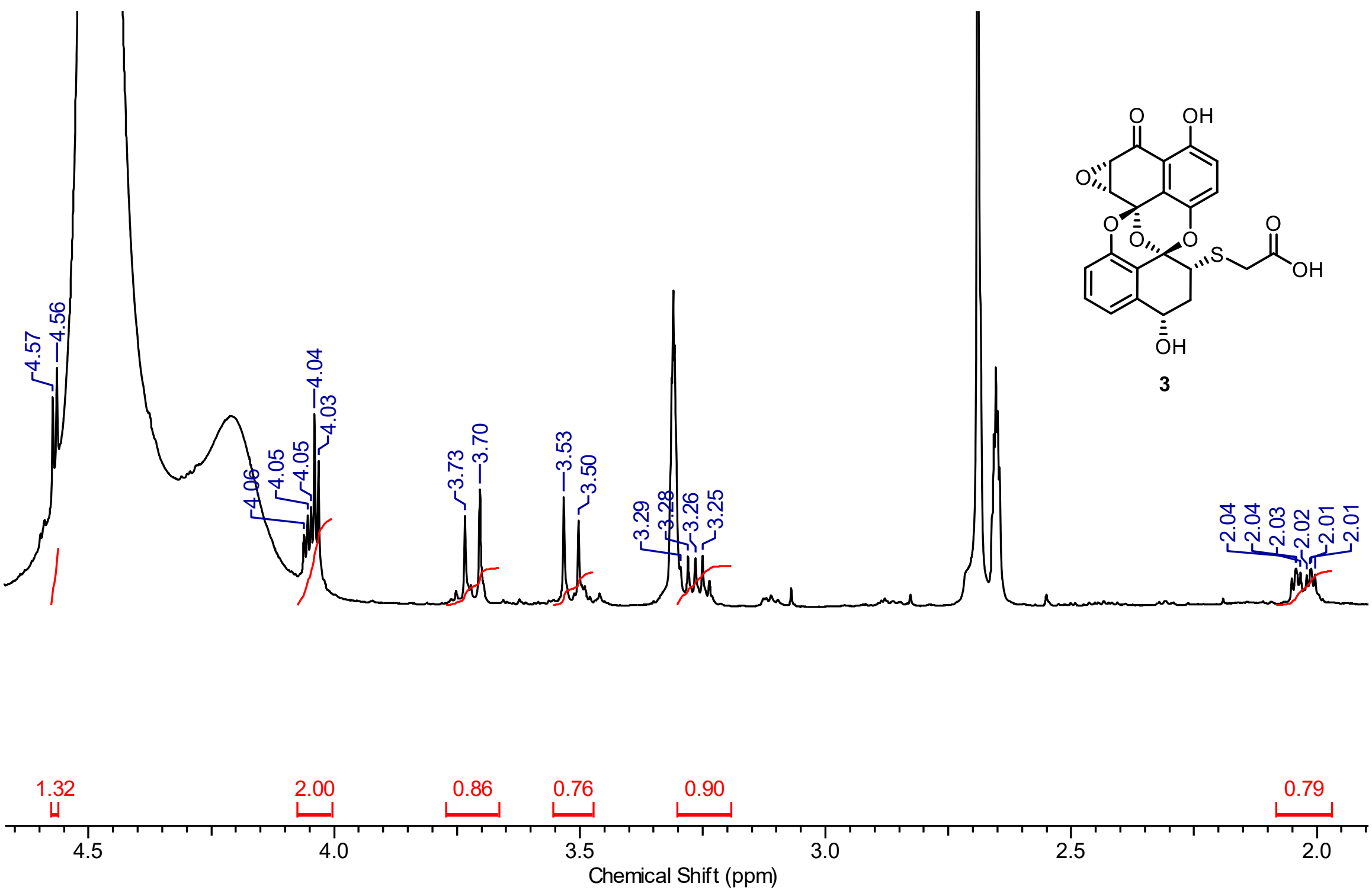

Figure S26c. Expansion of ^{1}H NMR spectrum of sulphopreussomerin C (**3**) **(**CD_3OD/DMSO-d_6, 3:2, 500 MHz

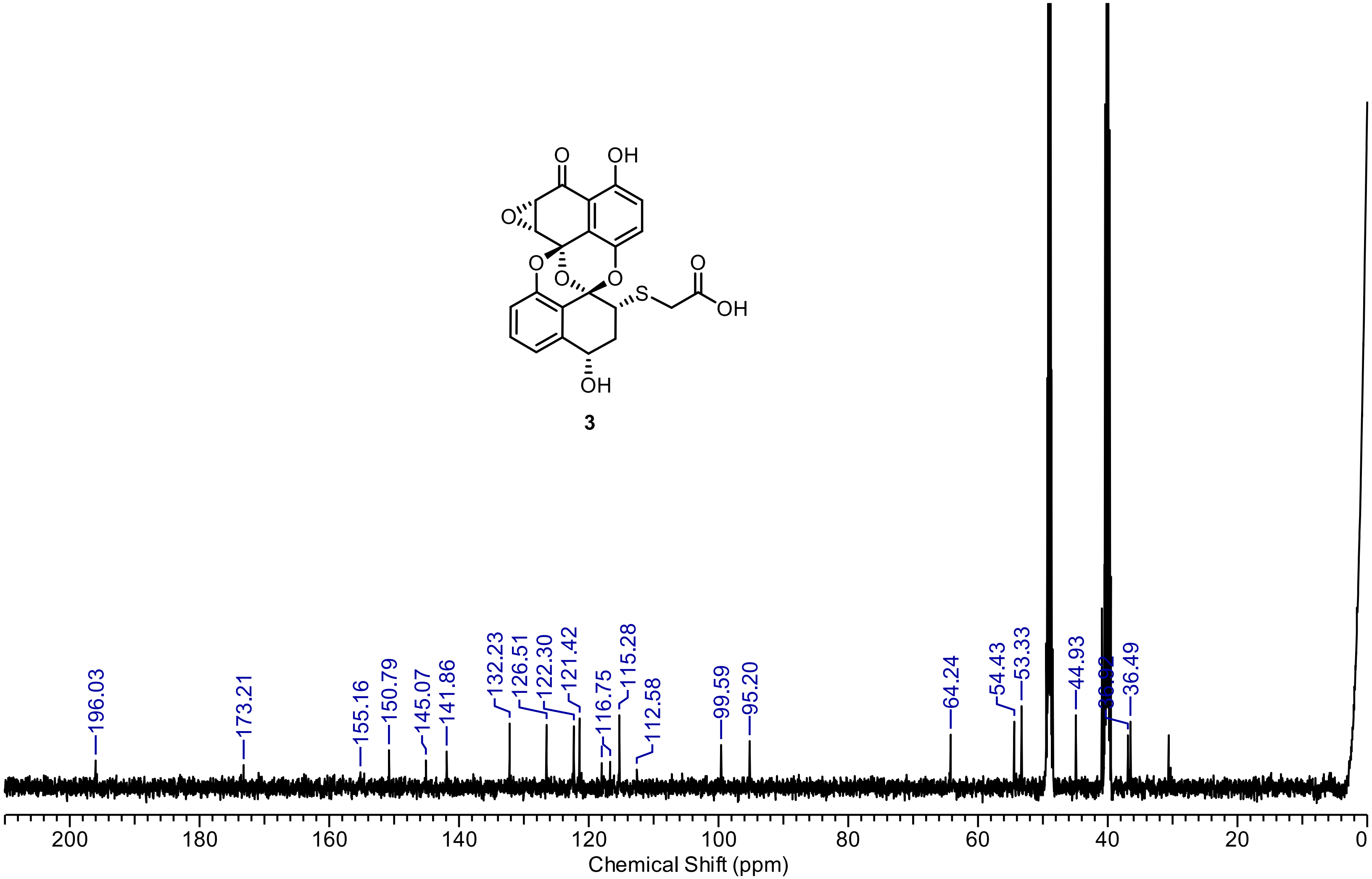

Figure S27. ^{13}C NMR spectrum of sulphopreussomerin C (**3**) (CD_3OD/DMSO-d_6, 3:2, 125 MHz)

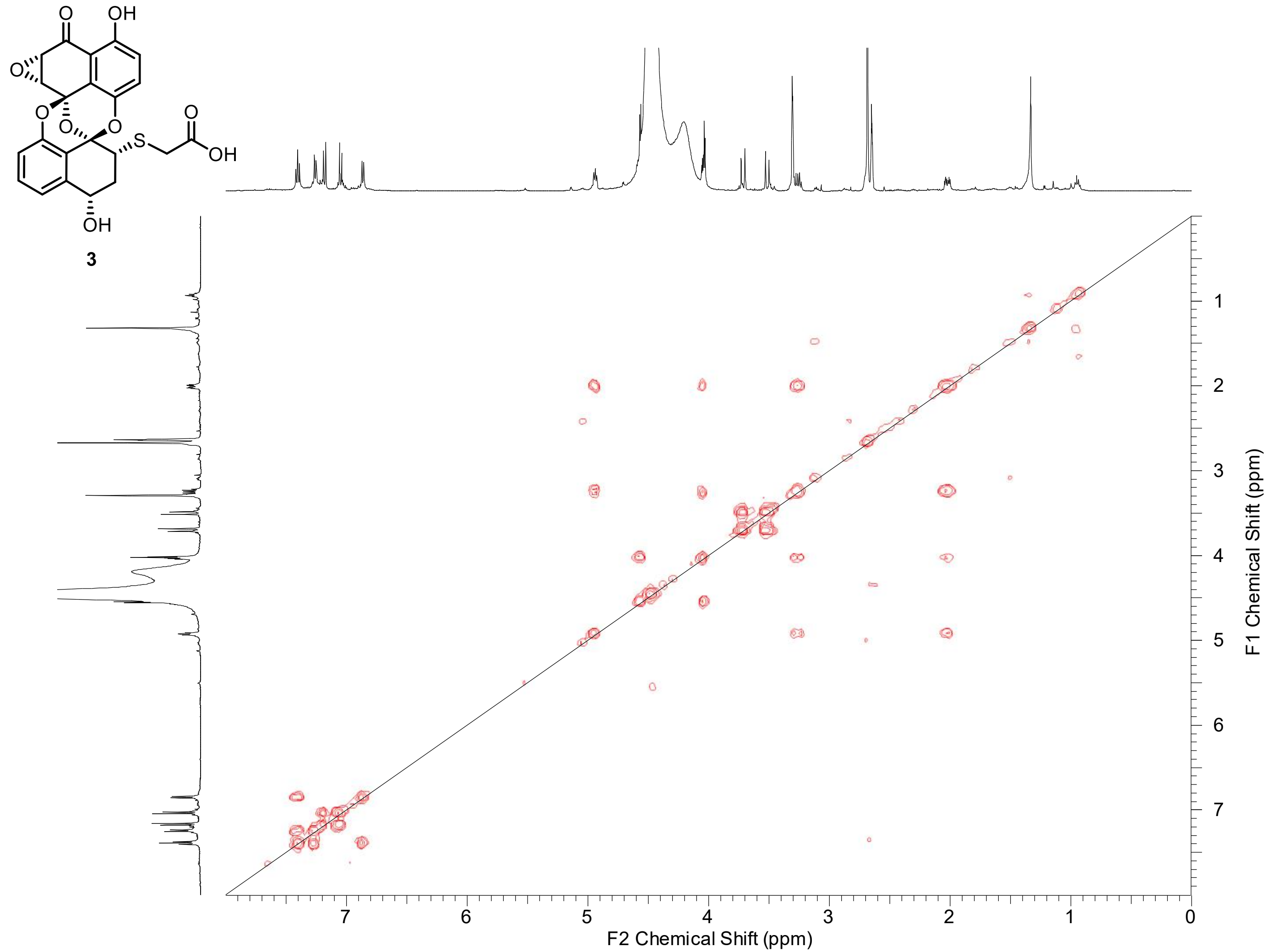

Figure S28. COSY spectrum of sulphopreussomerin C (**3**)

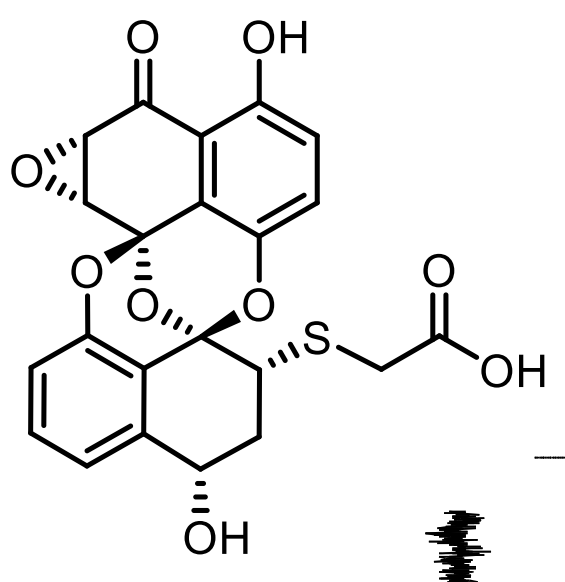

3

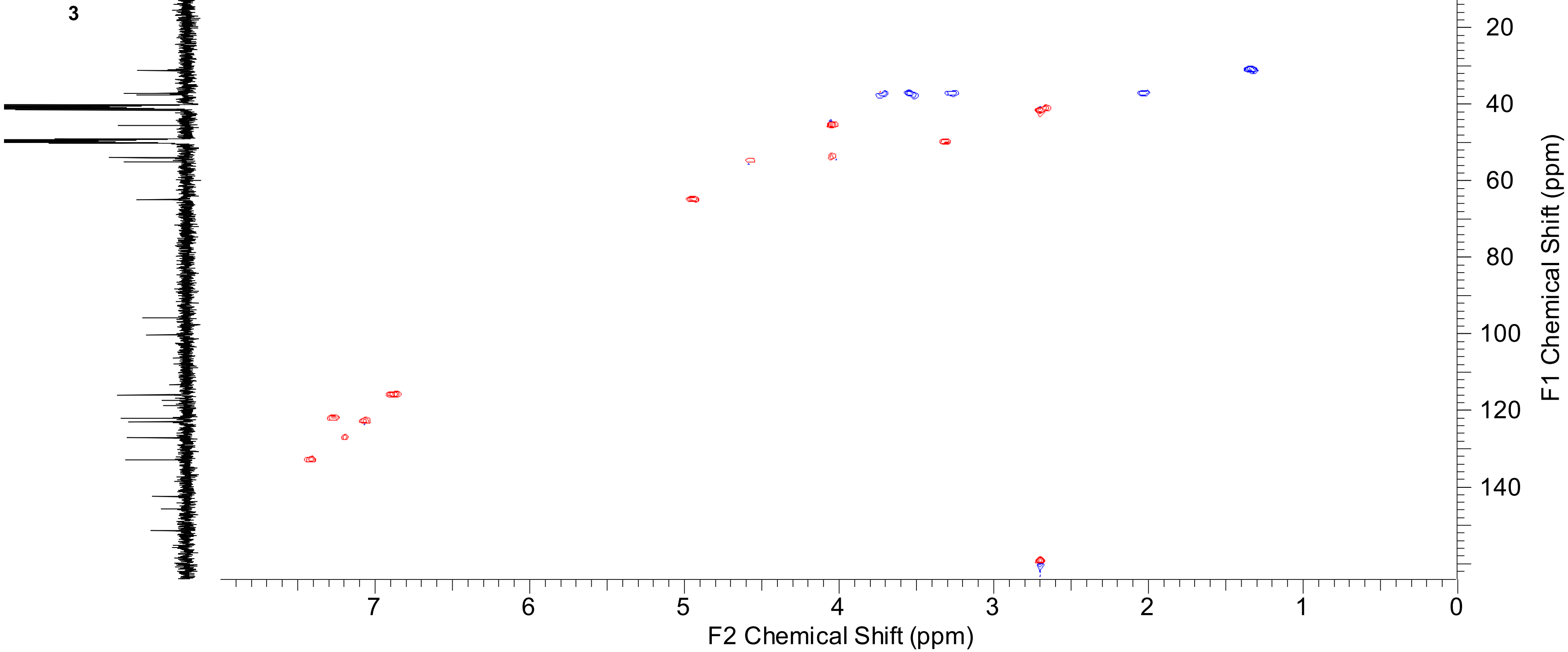

Figure S29. HSQC spectrum of sulphopreussomerin C (**3**)

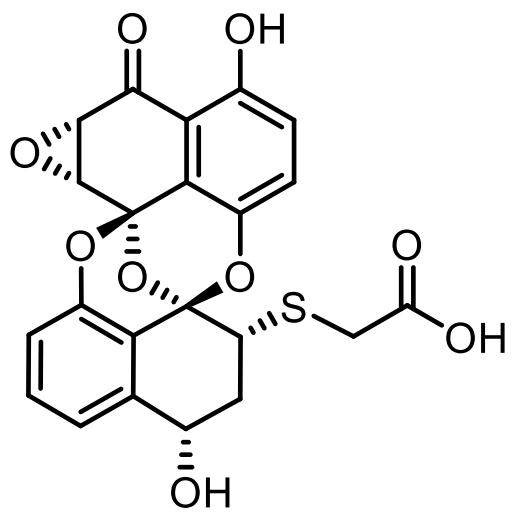

3

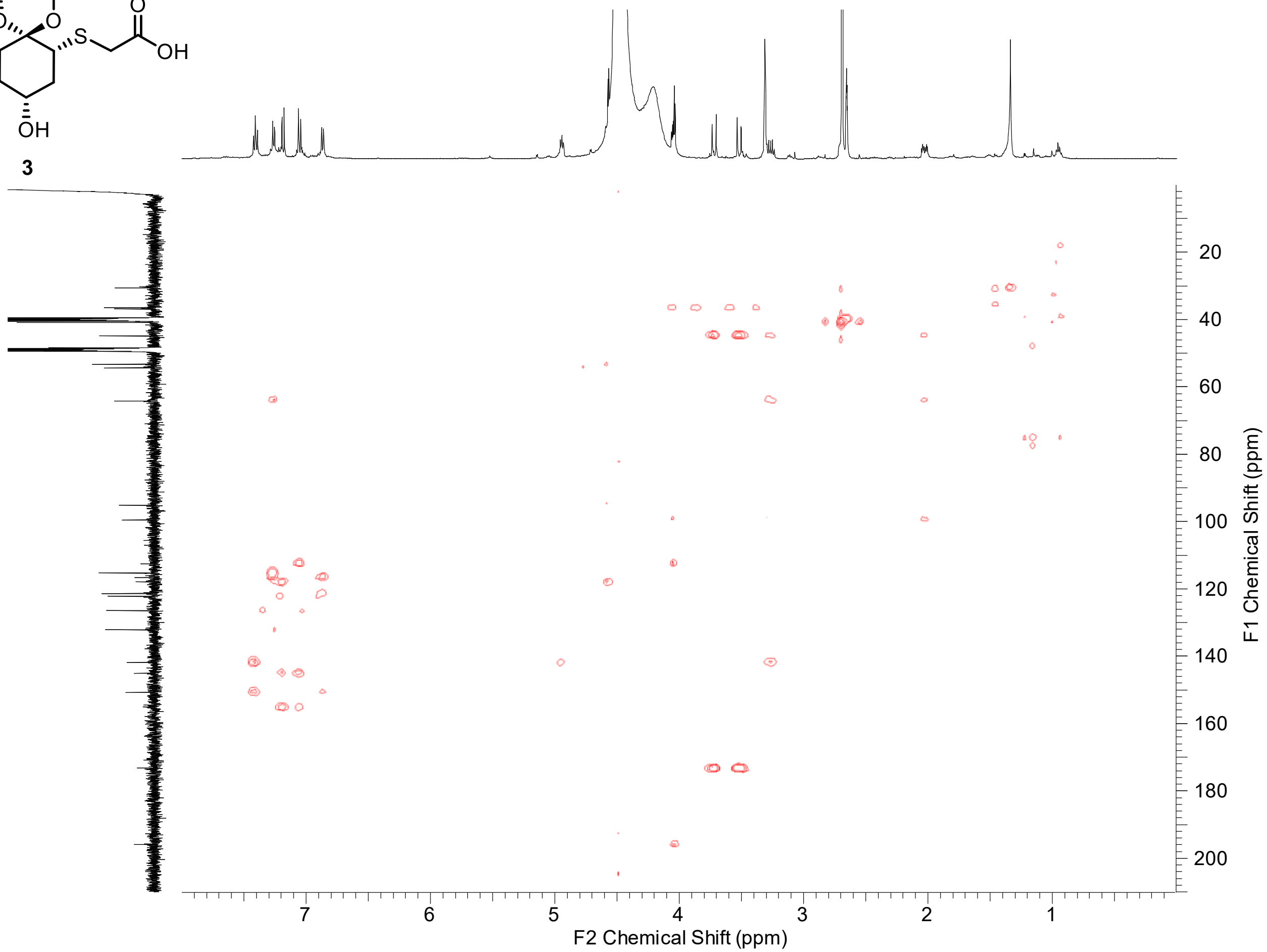

Figure S30. HMBC spectrum of sulphopreussomerin C (**3**)

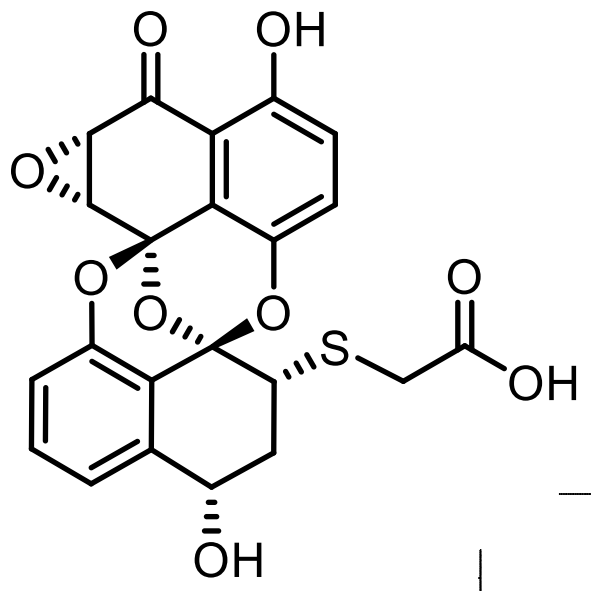

3

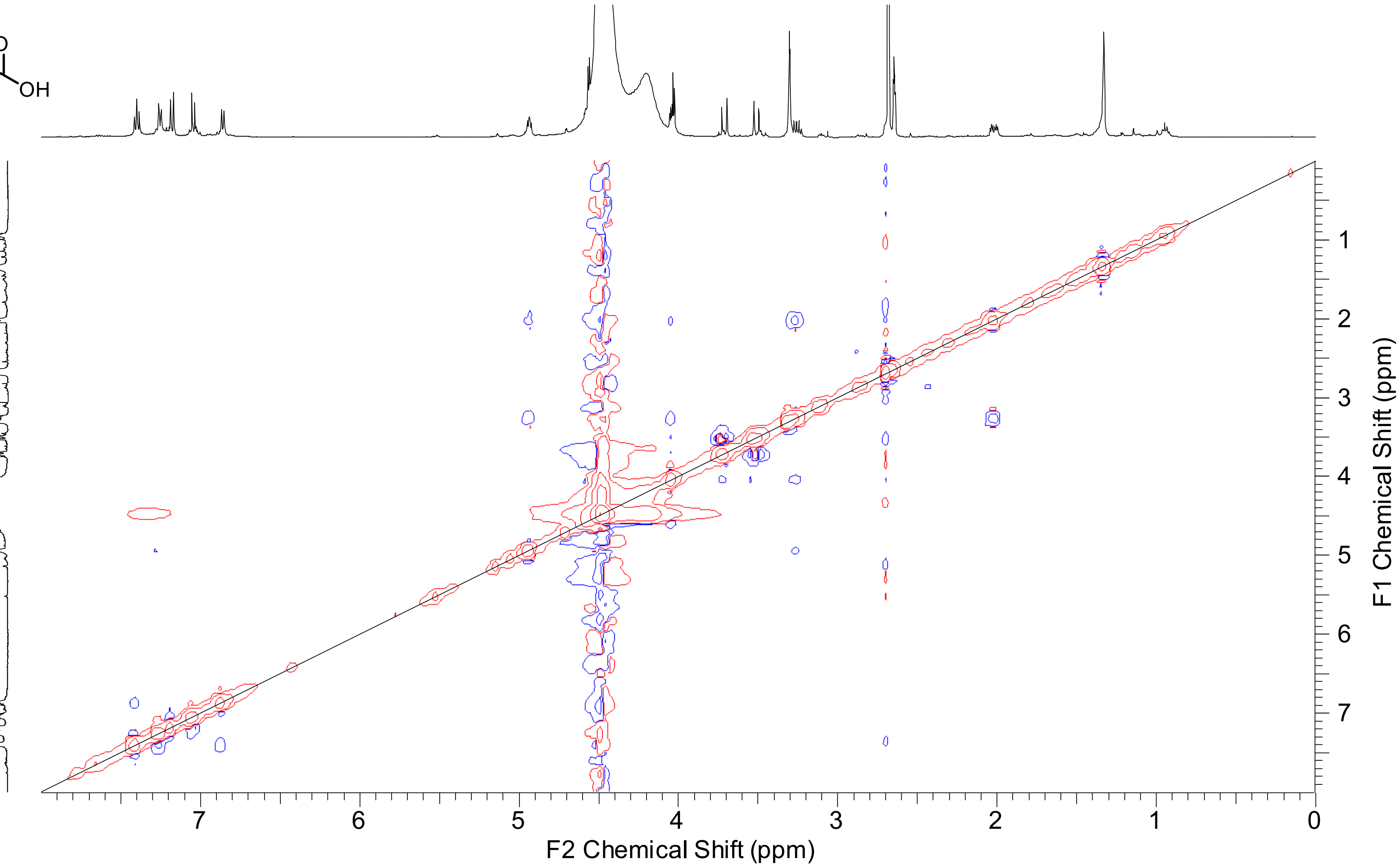

Figure S31. NOESY spectrum of sulphopreussomerin C (**3**)

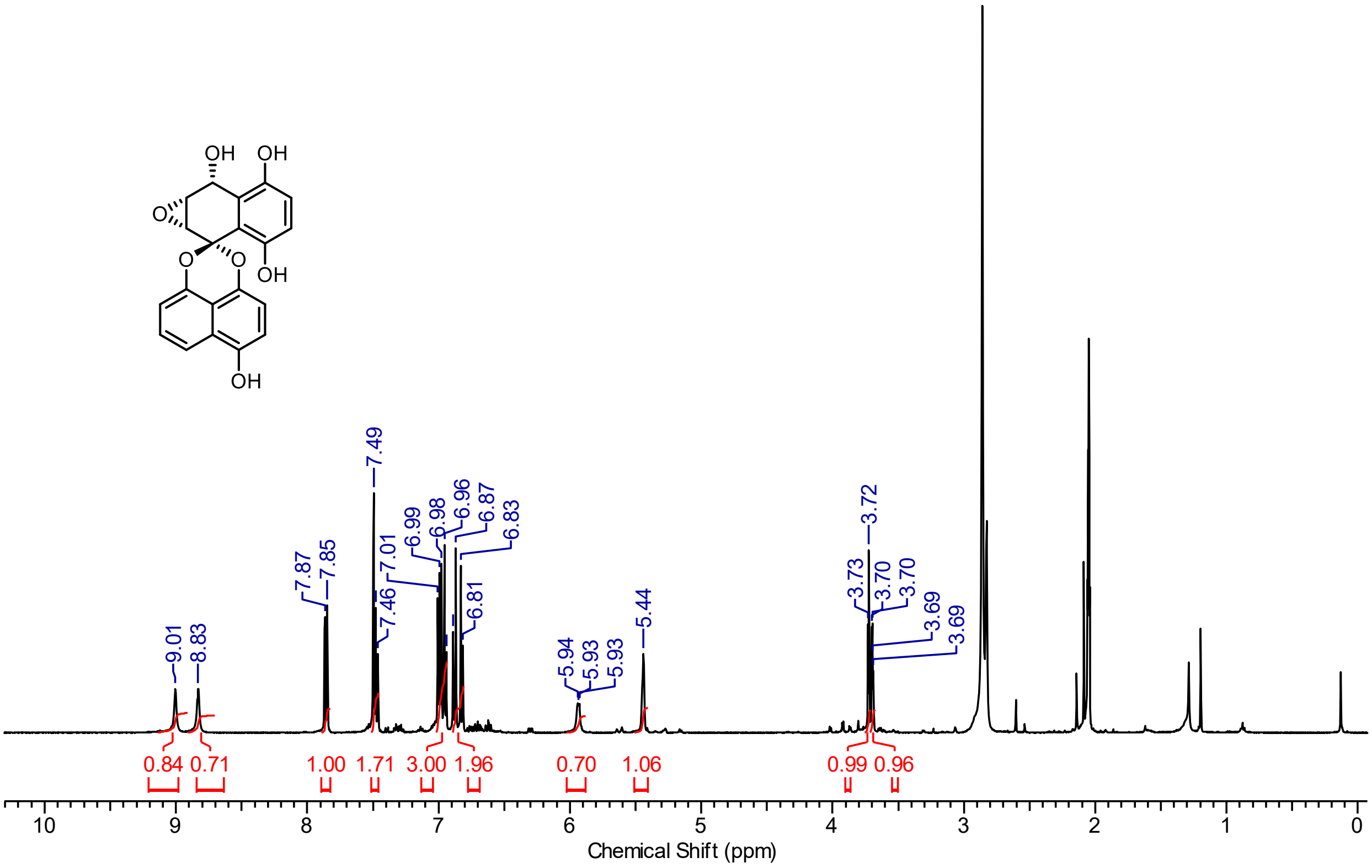

Figure S32. ^{1}H NMR spectrum of ascochytatin (**4**) ($(CD_3)_2CO$, 500 MHz)

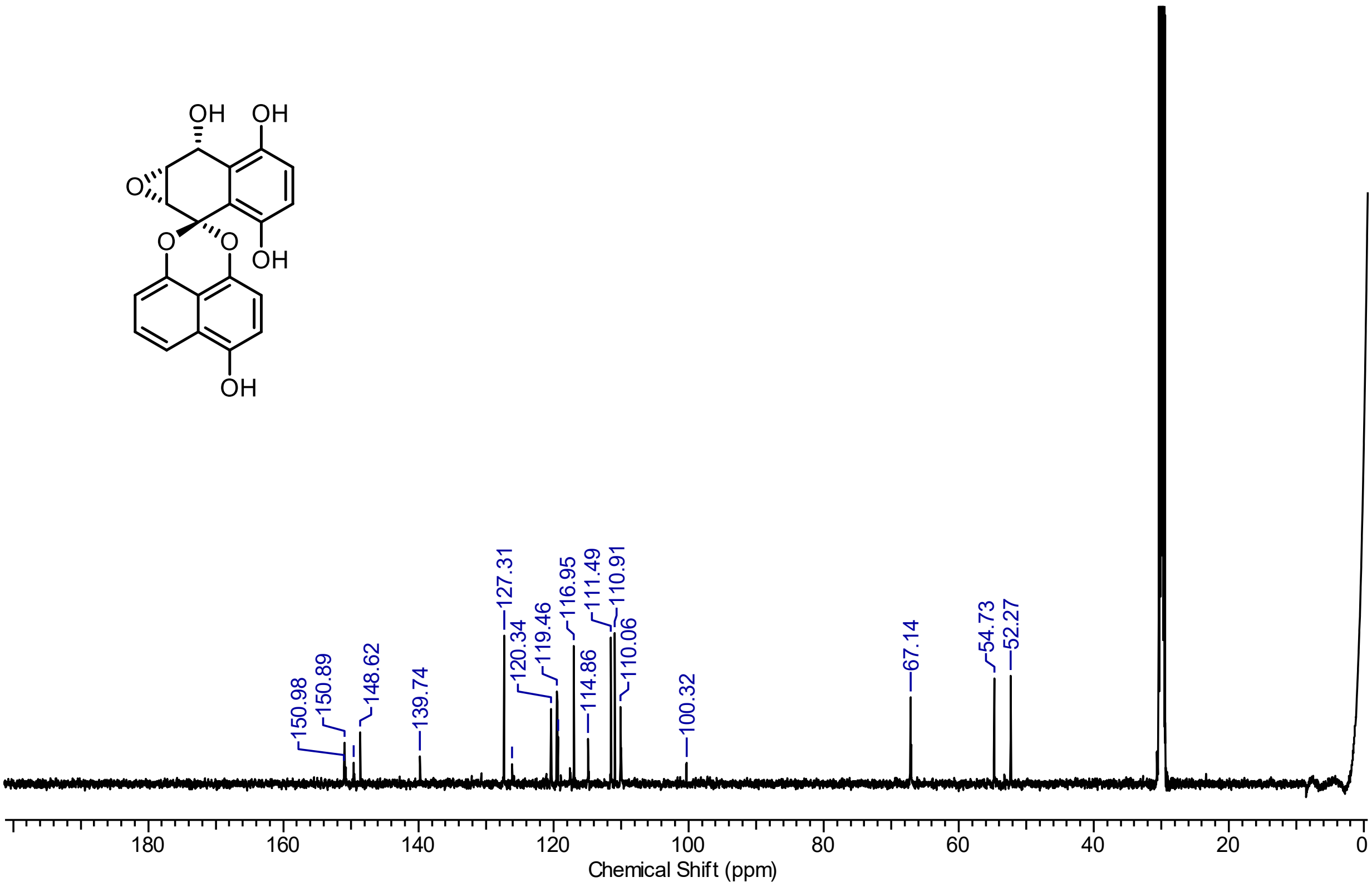

Figure S33. ^{13}C NMR spectrum of ascochytatin (**4**) ($(CD_3)_2CO$, 125 MHz)

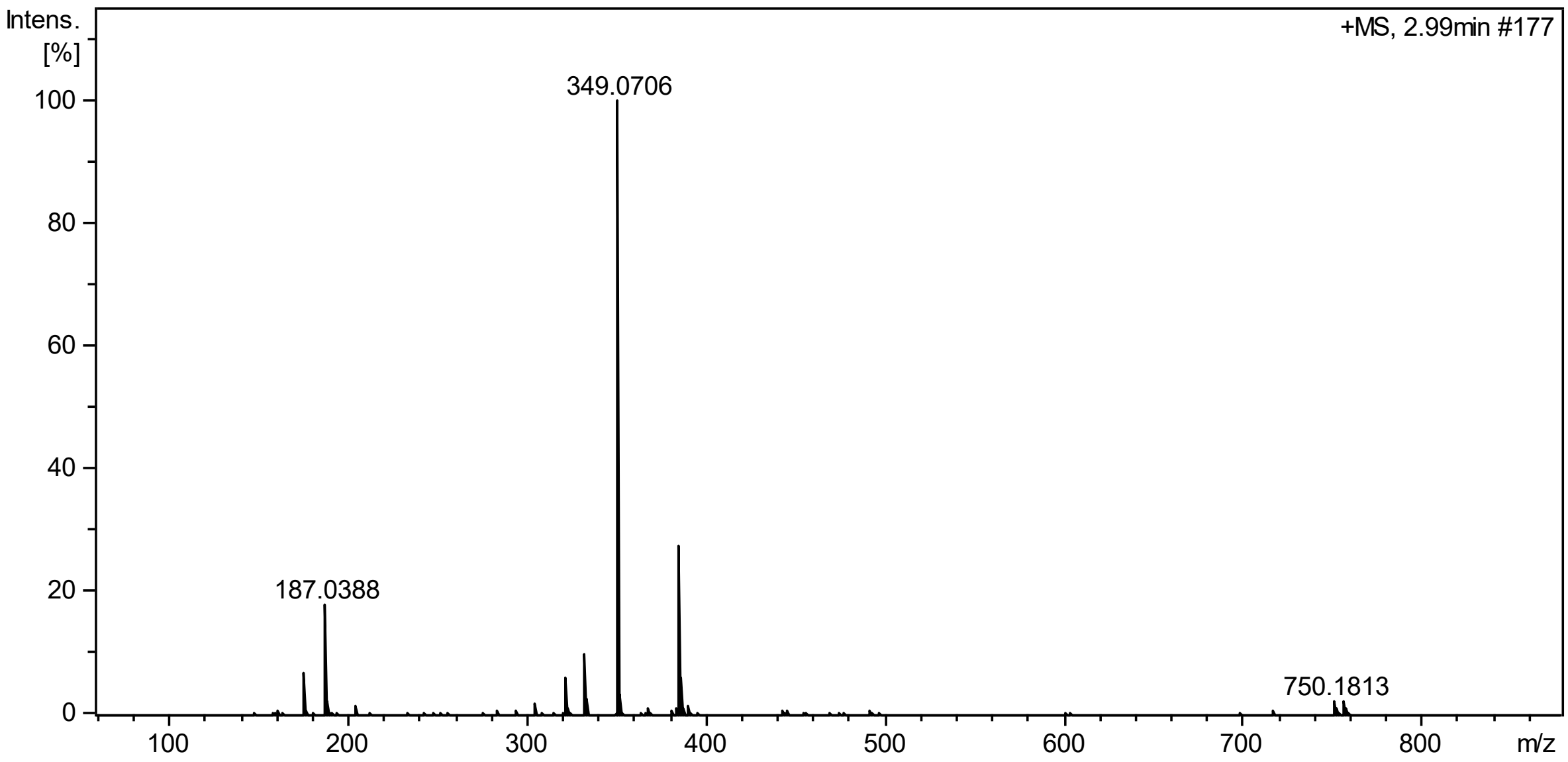

Figure S34. HR ESI-TOF MS spectrum of ascochytatin (**4**)

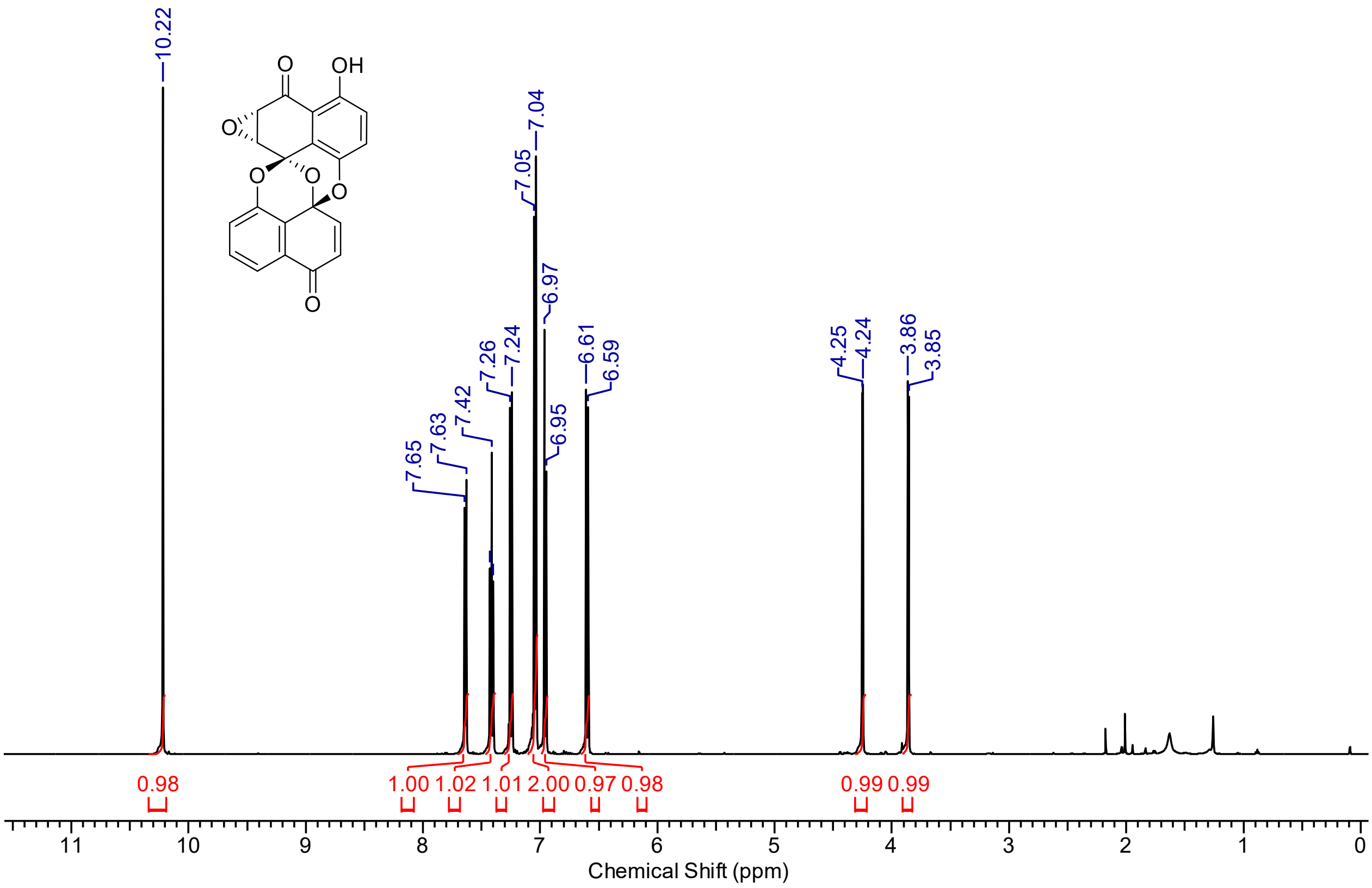

Figure S35. ^{1}H NMR spectrum of preussomerin G (**5**) ($CDCl_3$, 500 MHz)

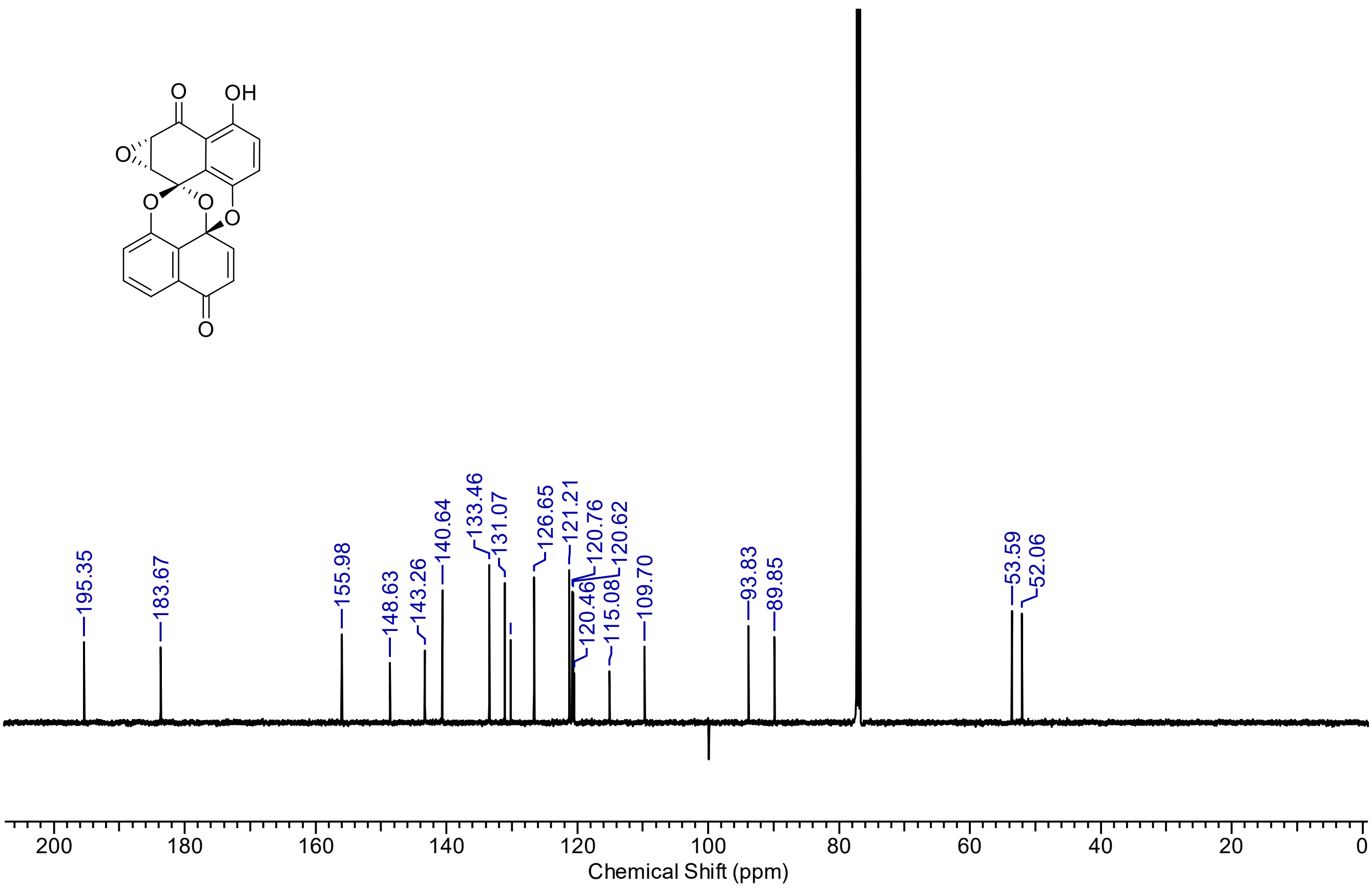

Figure S36. ^{13}C NMR spectrum of preussomerin G (**5**) ($CDCl_3$, 125 MHz)

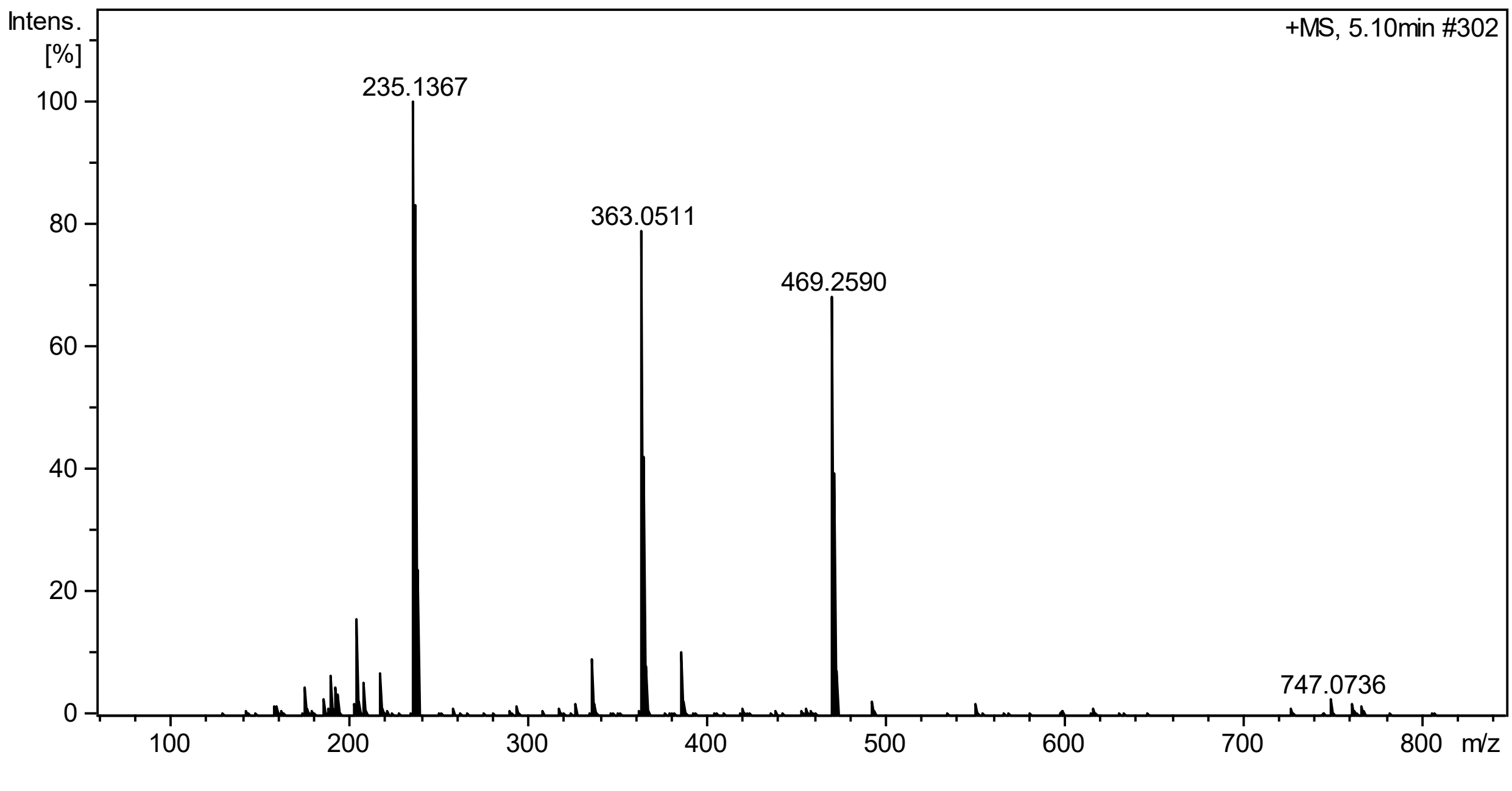

Figure S37. HR ESI-TOF MS spectrum of preussomerin G (**5**)

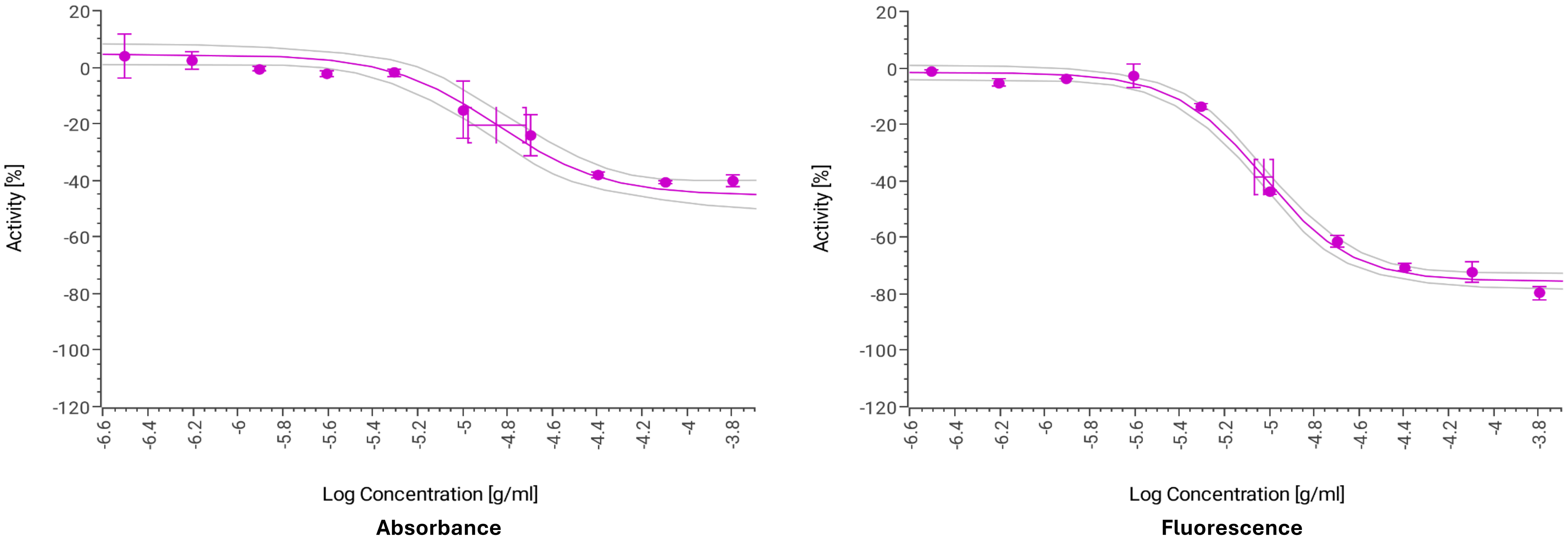

Figure S38. Dose-response curves of sulphopreussomerin A (**1**) against *Z. tritici* based on absorbance and fluorescence in vitro assays

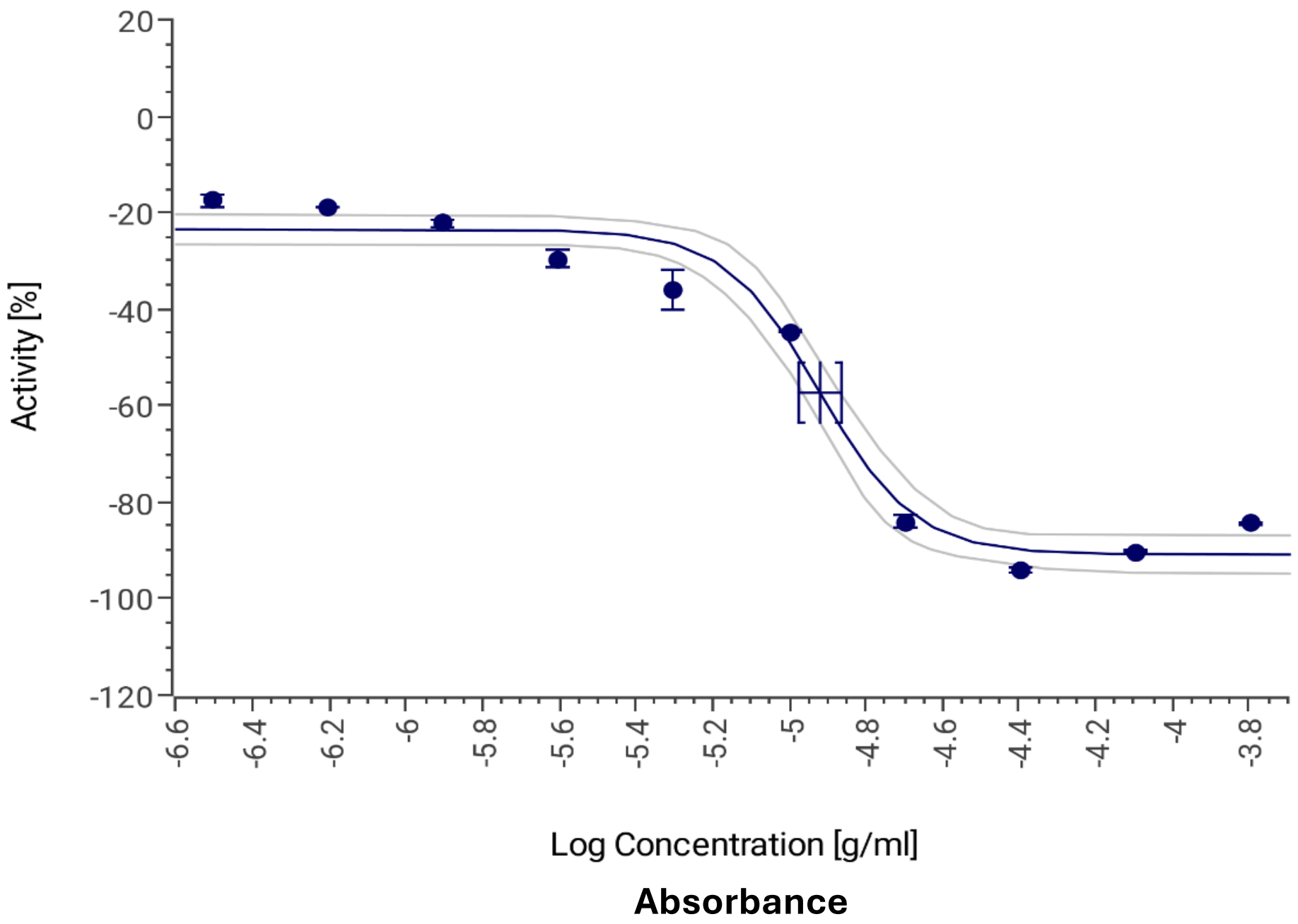

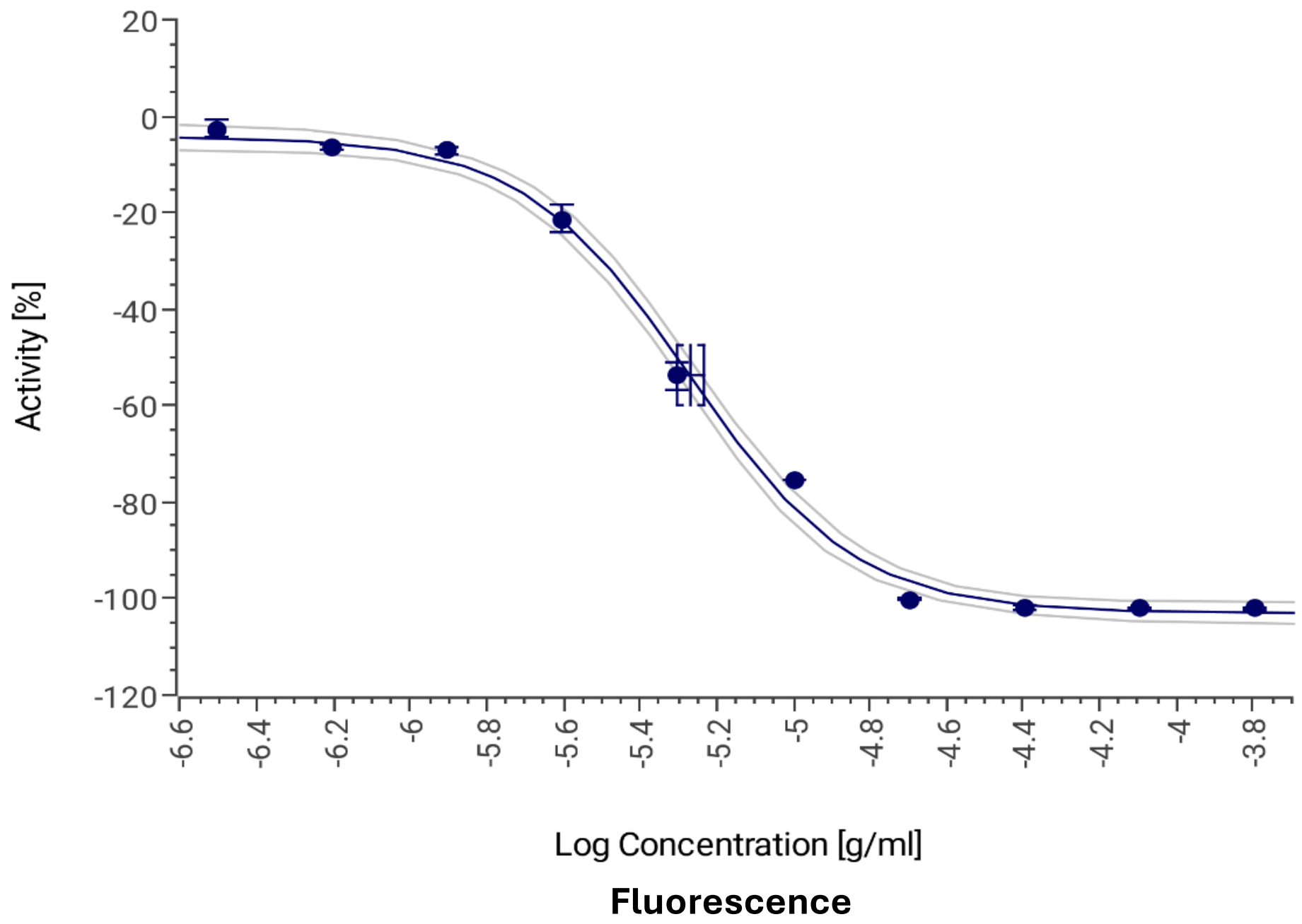

Figure S39. Dose-response curve of ascochytatin (**4**) against *Z. tritici* based on absorbance and fluorescence in vitro assays

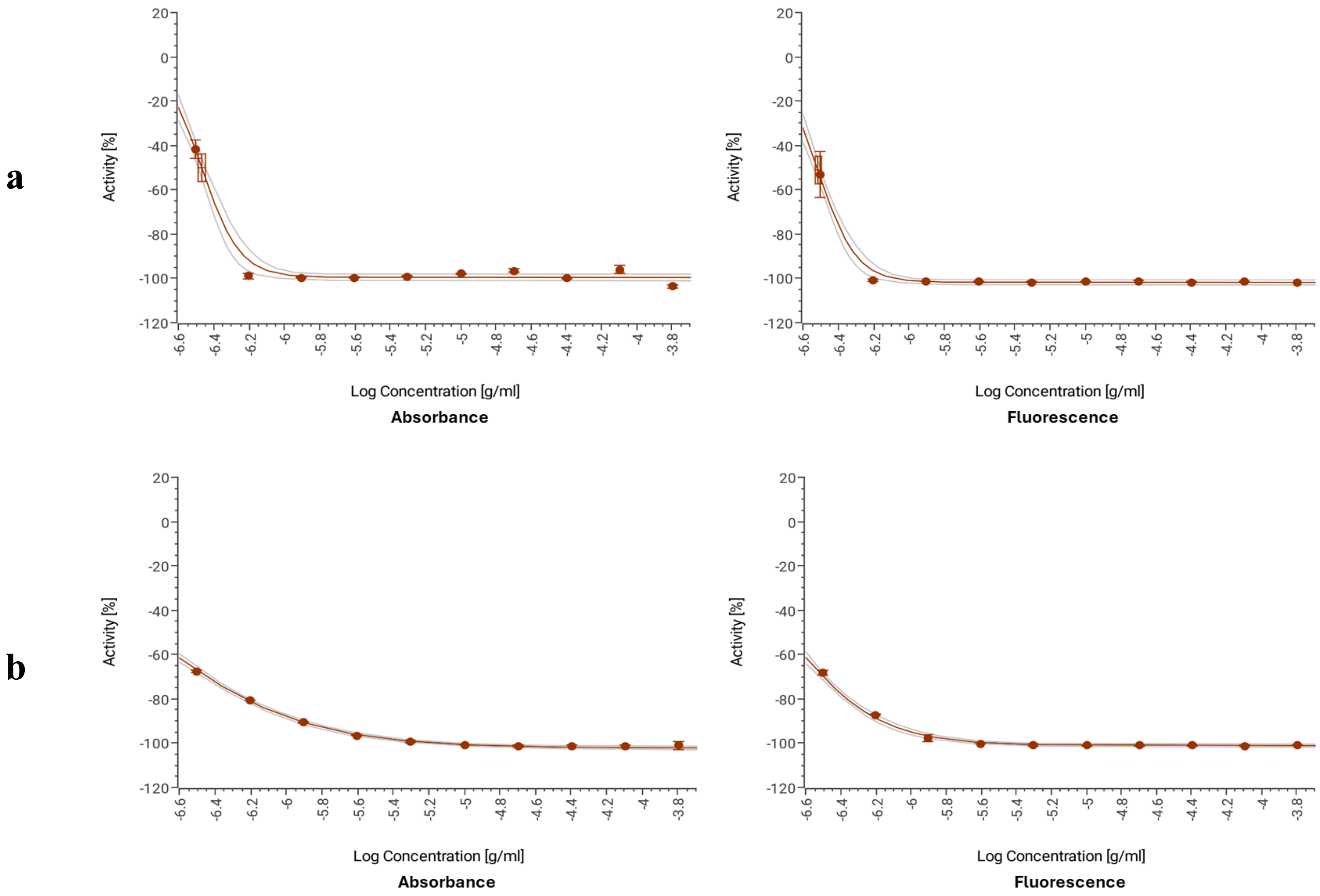

Figure S40. Dose-response curves of preussomerin G (**5**) against a) *Z. tritici* and b) *F. proliferatum* based on absorbance and fluorescence in vitro assays

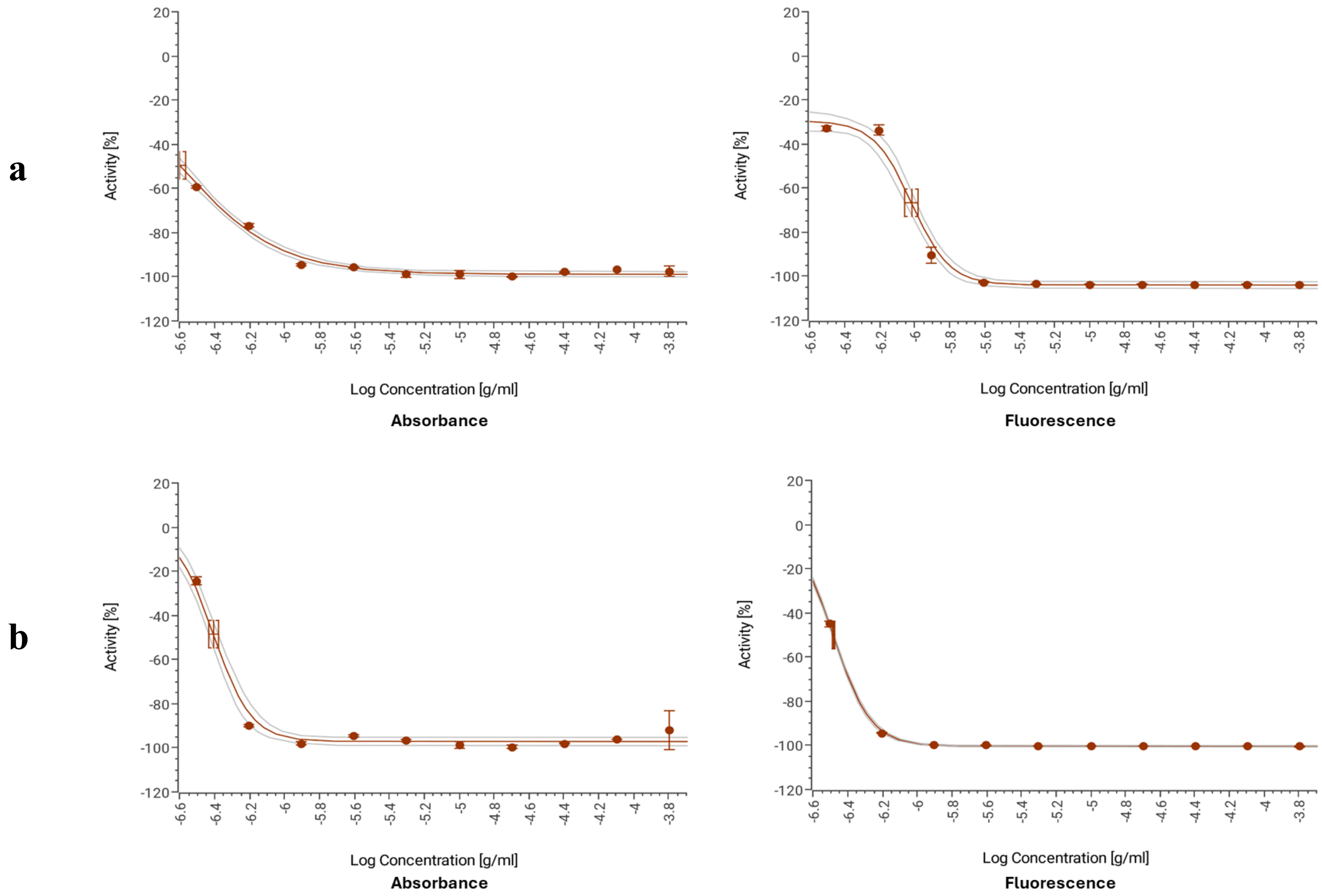

Figure S41. Dose-response curves of preussomerin G (**5**) against a) *C. acutatum* and b) *M. grisea* based on absorbance and fluorescence in vitro assays

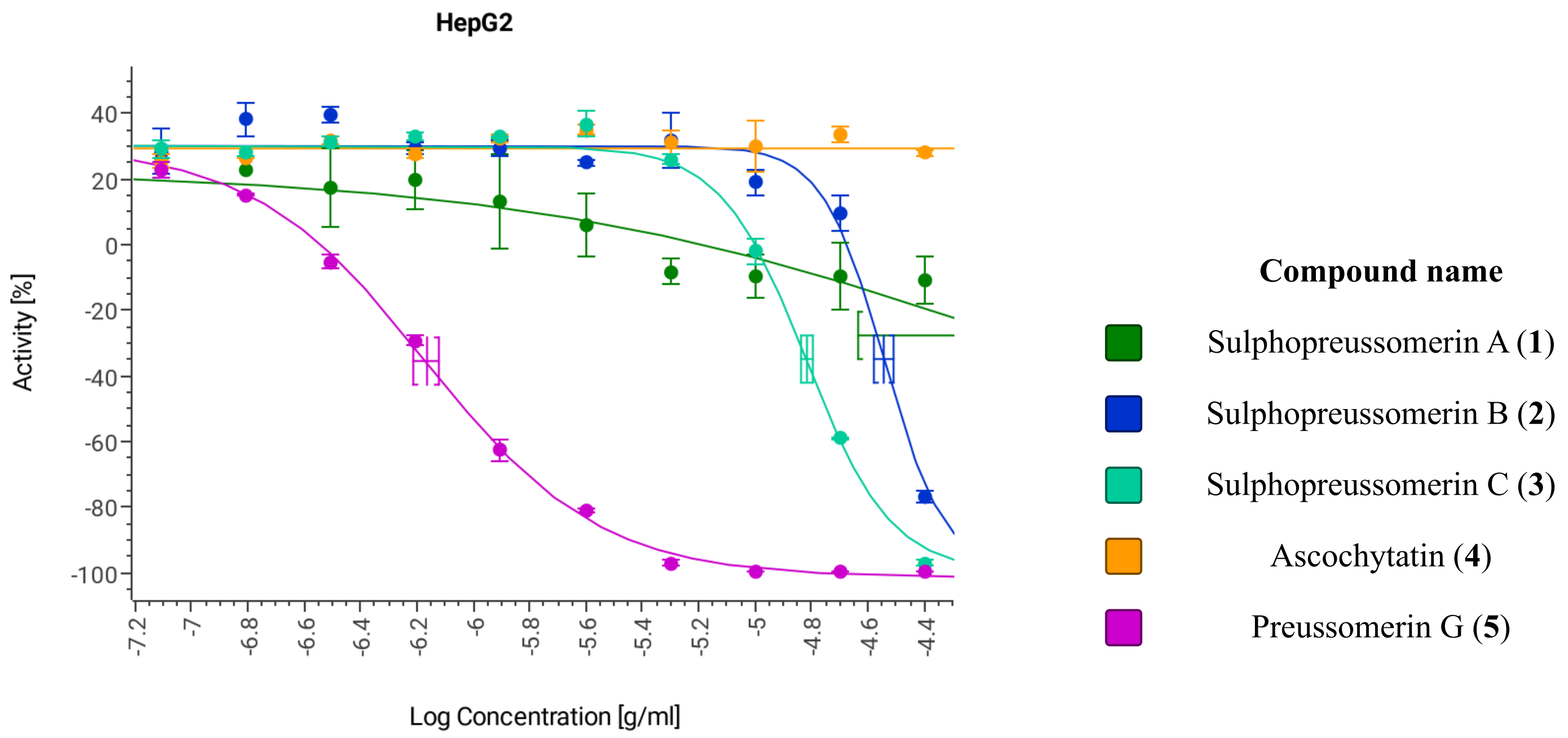

Figure S42. Dose-response curves of compounds **1-5** against the Hep-G2 human cell line in vitro assays

Table S5. Sequence of the ITS region of the fungus *Pyrenochaetopsis indica*

rDNA-ITS region (5′→3′, contig length = 1054bp)						
1	AAGTAAAAGT	CGTAACAAGG	TTTCCGTAGG	TGAACCTGCG	GAAGGATCAT	TATCAAAACA
61	GCGGCCTTCG	GGCCTCGCTG	CACCCTTGTC	TTTTGCGCAC	CGTATTGTTT	CCTCGGCGGG
121	CTTGCCTGCC	GGTTGGACAT	CATAAAACCT	TTTTGTAGTT	GCAATCAGCG	TCAGAAAAAC
181	AATAATTATT	ACAACTTTCA	ACAACGGATC	TCTTGGTTCT	GGCATCGATG	AAGAACGCAG
241	CGAAATGCGA	AAAGTAGTGT	GAATTGCAGA	ATTCAGTGAA	TCATCGAATC	TTTGAACGCA
301	CATTGCGCCC	CTTGGTATTC	CATGGGGCAT	GCCTGTTCGA	GCGTCATTTT	GTACCCTCAA
361	GCACTGCTTG	GTGTTGGGCG	TTTGTCCTGC	AGAGGACTCG	CCTGAAAGCG	ATTGGCGGCC
421	AACGTAGTCG	TGGCAGAGCG	CAGCACAATC	TCGCGTCTCT	CCCCTCTGCG	TCGGCGTCCA
481	TAAGCCTACA	ATCTCAACGT	TTGACCTCGG	ATCAGGTAGG	GATACCCGCT	GAACTTAAGC
541	ATATCAATAA	GCGGAGGAAA	AGAAACCAAC	AGGGATTGCC	CTAGTAACGG	CGAGTGAAGC
601	GGCATCAGCT	CAAATTTGAA	ATCTGGCTCT	TTCAGAGTCC	GAGTTGTAAT	TTGCAGAGGG
661	CGCTTTGGAG	TCGGCAGCGG	TCCAAGTTCC	TTGGAACAGG	ACGTCACAGA	GGGTGAGAAT
721	CCCGTACGTG	GTCGCTGACC	TACTCCGTGT	AAAGCCCCTT	CGACGAGTCG	AGTTGTTTGG
781	GAATGCAGCT	CTAAATGGGA	GGTAAATTTC	TTCTAAAGCT	AAATACTGGC	CAGAGACCGA
841	TAGCGCACAA	GTAGAGTGAT	CGAAAGATGA	AAAGCACTTT	GGAAAGAGAG	TCAAACAGCA
901	CGTGAAATTG	TTGAAAGGGA	AGCGCTTGCA	GCCAGACTTG	TTTGTAGTTG	CTCATCCGGG
961	CTTTTGCCCG	GTGCATTCTT	CTGCGAACAG	GCCAGCATCA	GTTTGGGCGG	TTGGATAAAG
1021	GCCTCTGTCA	CGTACCTTCT	CTCGGGAAGG	CCTT		

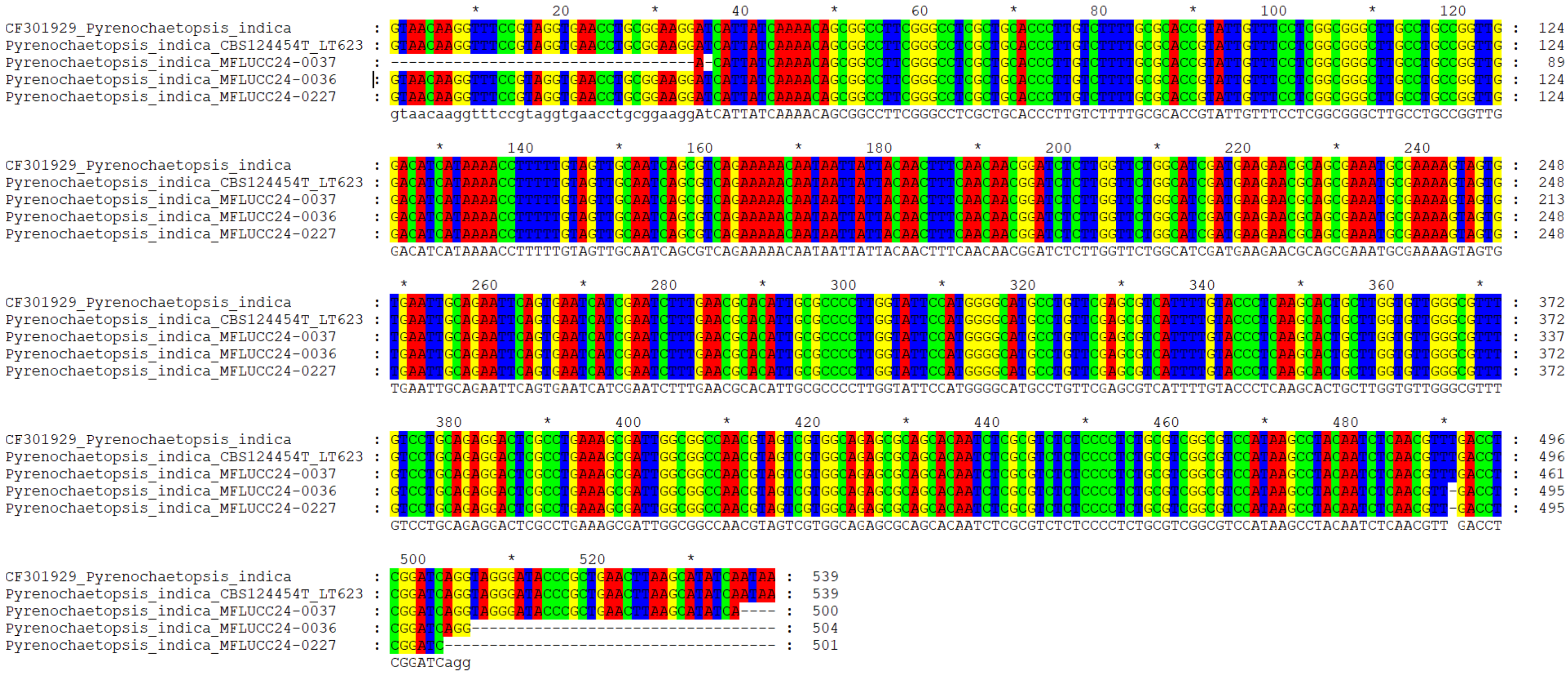

Figure S43. Alignment of the complete ITS1-5.8S-ITS2 region sequences of the producer strain CF-301929, the Ex-type strain of *Pyrenochaetopsis indica* CBS124454*, P. indica* strain MFLUCC 24-0037, *P. indica* strain MFLUCC 24-0036, and *P. indica* strain MFLUCC 24-0227 performed by GeneDoc software
